# Synthesis of 5-Hydrazino-2-cyclopentenone Derivatives by a Gold(I)-Catalyzed Cycloisomerization/Hetero-Diels–Alder/Ring-Opening Tandem Reaction of Enynyl Acetates

**DOI:** 10.1021/acs.joc.3c00310

**Published:** 2023-05-12

**Authors:** Dina Scarpi, Nunzia Favale, Ernesto G. Occhiato

**Affiliations:** Dipartimento di Chimica “U. Schiff”, Università Degli Studi di Firenze, Via Della Lastruccia 13, Sesto Fiorentino (FI) 50019, Italy

## Abstract

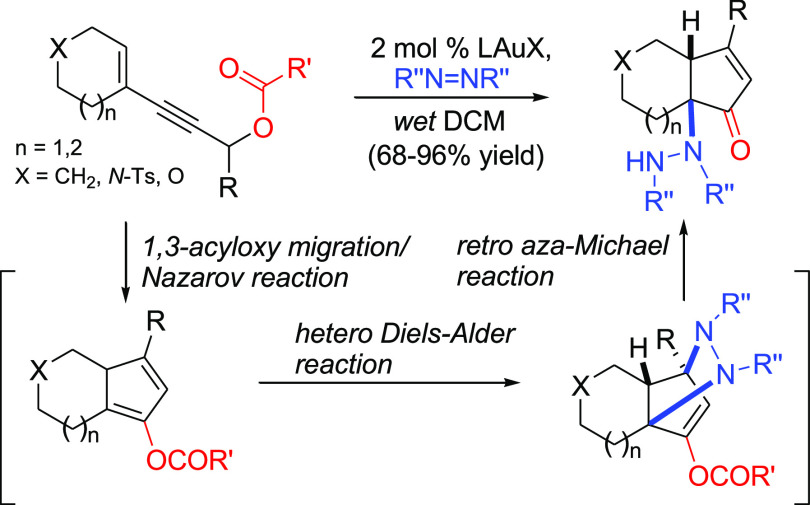

A highly efficient, one-pot synthesis of ring-fused 5-hydrazino-2-cyclopentenone derivatives is achieved by a gold(I)-catalyzed cycloisomerization/hetero-Diels–Alder/ring-opening tandem reaction of suitable enynyl acetates. By mixing the latter with a dialkylazodicarboxylate in the presence of a gold(I) catalyst, the 1,3-acyloxy migration/Nazarov cyclization process leads to dienyl acetate intermediates which are trapped by the heterodienophile present in situ. This provides strained intermediates which undergo highly regioselective ring opening by a retro aza-Michael reaction promoted by traces of water, eventually yielding the target compounds. Six- and seven-membered ring-fused cyclopentenones and piperidine- and tetrahydropyran-fused cyclopentenones bearing a pendant hydrazino functionality on a bridgehead carbon atom can be obtained in high yield (68–96%) by this approach.

## Introduction

**Figure 1:**
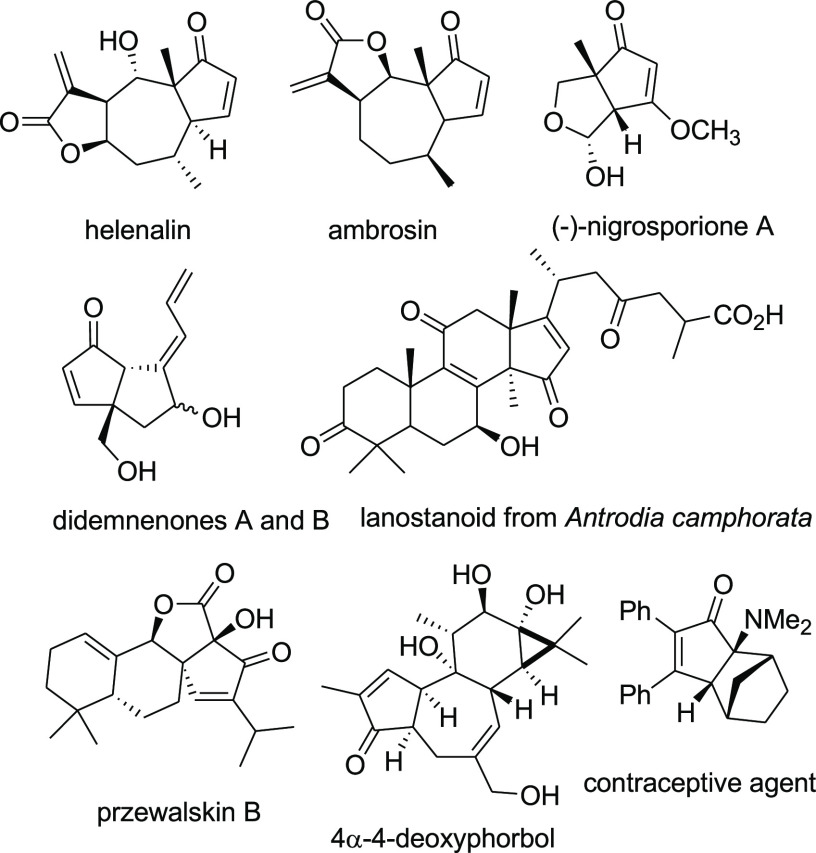
Examples of natural and bioactive compounds containing a ring-fused 2-cyclopentenone moiety.

The 2-cyclopentenone ring is found in a variety of natural and biologically active compounds possessing a high structural diversity, many of which embed a ring-fused cyclopentenone moiety (Figure 1).^1^ The importance of the 2-cyclopentenones is further enhanced by a variety of chemical transformations that can be carried out on them, which explains their popularity not only as benchmark substrates for many chemical transformations but also as starting materials in the synthesis of more complex compounds.^2^ Thus, due to their privileged nature, many methods have been developed to access diversely functionalized 2-cyclopentenones.^1−3^

Among gold-mediated syntheses of 2-cyclopentenones,^1,2,4−8^ those based on gold(I)-catalyzed cycloisomerization of propargyl alcohol derivatives have especially shown their efficacy in providing these valuable compounds.^1,2,9−15^ We have recently contributed to this field with the synthesis of cyclopentenones fused with heterocyclic rings and their transformation into some natural compounds.^16−19^ In his pioneering work on the gold-catalyzed cycloisomerization of enynyl esters to 2-cyclopentenones,^20^ occurring via a 1,3-acyloxy migration/Nazarov cyclization sequence,^21,22^ Zhang showed that the target compounds could be obtained through the hydrolysis of the cyclopentadienyl esters formed in the process when the reaction was carried out in “wet” dichloromethane. Under anhydrous conditions, instead, the cyclopentadienyl esters could be isolated in high yield.^15,20^

**Scheme 1:**
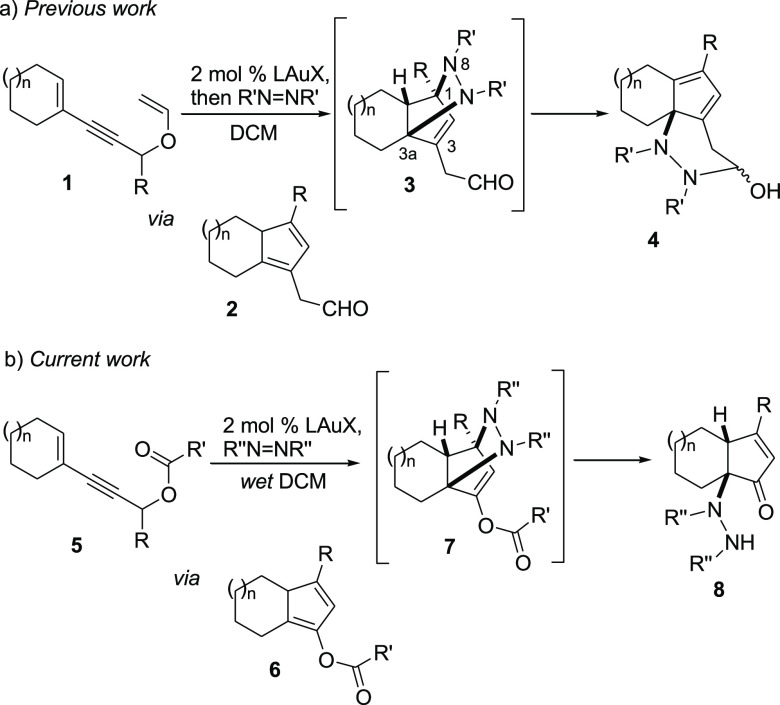
Previous (a) and Current Studies (b) on Cycloisomerization/HDA/Ring-Opening Tandem Reactions

We have recently reported on the synthesis of ring-fused, cyclopentadienyl hydrazine derivatives **4** (Scheme 1a) by a one-pot, cascade process entailing the cycloisomerization of suitably substituted propargyl vinyl ethers **1**,^23−26^ the hetero-Diels–Alder (HDA) reaction of cyclopentadiene intermediates **2** with a dialkylazodicarboxylate, and the acid-catalyzed ring opening of cycloadducts **3**.^27^ Since in the cycloisomerization of the corresponding propargyl esters **5** to 2-cyclopentenones, a cyclopentadienyl ester intermediate (**6**) is formed,^20^ we were interested in evaluating whether the latter could react with a dialkylazodicarboxylate present in the reaction mixture to provide the corresponding 5-hydrazino-2-cyclopentenone derivative **8**(28) bearing an *N*-substituted quaternary center,^29^ through a selective C–N bond cleavage in cycloadduct **7** (Scheme 1b). In our previous work, we had demonstrated that the highly regioselective C_1_–N_8_ bond cleavage occurs in the presence of either the gold(I) catalyst or traces of mineral acids.^27^ In the analogous process carried out on propargyl esters **5**, the stage which is set for the C–N cleavage after the cycloaddition (i.e., intermediate **7**) is different (from **3**), but we nonetheless hoped for a similarly regioselective C–N cleavage by a retro aza-Michael addition upon hydrolysis of the suitably positioned ester group.

## Results and Discussion

We carried out our first experiment by adding a solution of substrate **9** and diethyl azodicarboxylate (DEAD) (1 equiv) in CH_2_Cl_2_ (distilled over CaH_2_) to a solution of the IPrAuNTf_2_ (2 mol %) catalyst in the same solvent (Table 1, entry 1). Monitoring the reaction by thin layer chromatography (TLC), we found that the conversion of the starting material into reaction products was very slow as the former completely disappeared after 3 h. Gratifyingly, after an aqueous work-up, the ^1^H NMR analysis of the crude reaction mixture revealed the presence of desired product **12** and the corresponding *N*-acetylated compound **13** in an approximately 5:1 ratio.^30^ When we carried out the same reaction in undistilled CH_2_Cl_2_ (entry 2),^31^ the consumption of the starting material was still very slow, but after work-up, we recorded a very clean ^1^H NMR spectrum with the signals of product **12** only, which was obtained in 86% yield after chromatography. Better results were obtained by using commercial *t*-Bu_3_PAuNTf_2_ as the catalyst, the reaction being complete in 30 min and providing **12** in 94% yield (entry 3).

**Table 1:**
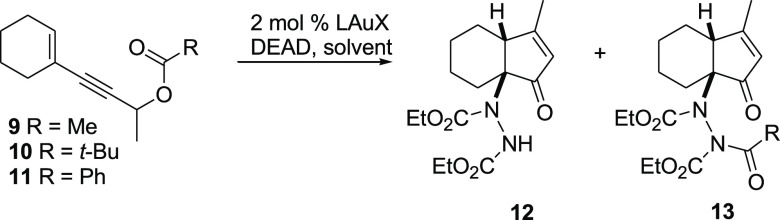
Survey of the Conditions for the Tandem Reaction Leading to 12^a^

entry	catalyst	*R*	solvent	time (min)	**12**^b^	**13**^b^
1	IPrAuNTf_2_	CH_3_	CH_2_Cl_2_^c^	180	84	16
2	IPrAuNTf_2_	CH_3_	CH_2_Cl_2_^d^	180	100 (86)^e^	
3	*t*-Bu_3_PAuNTf_2_	CH_3_	CH_2_Cl_2_^d^	30	100 (94)^e^	
4	*t*-Bu_3_PAuNTf_2_	CH_3_	CH_2_Cl_2_^c^	60	50	50
5	*t*-Bu_3_PAuNTf_2_	CH_3_	“wet” CH_2_Cl_2_^f^	35	100 (79)^e^	
6	*t*-Bu_3_PAuNTf_2_	CH_3_	“wet” CH_2_Cl_2_^g^	30	100 (95)^e^	
7	Ph_3_PAuCl/AgNTf_2_	CH_3_	CH_2_Cl_2_^d^	30	100 (94)^e^	
8	Ph_3_PAuCl/AgOTf	CH_3_	CH_2_Cl_2_^d^	30	100 (86)^e^	
9	Ph_3_PAuCl/AgSbF_6_	CH_3_	CH_2_Cl_2_^d^	30	100 (95)^e^	
10	Ph_3_PAuCl/AgSbF_6_	CH_3_	CH_2_Cl_2_^g^	30	100 (95)^e^	
11	*t*-Bu_3_PAuNTf_2_	CH_3_	toluene^h^	420	100 (91)^e^	
12	*t*-Bu_3_PAuNTf_2_	CH_3_	DCE^h^	190	83 (74)^e^	
13	*t*-Bu_3_PAuNTf_2_	(CH_3_)_3_C	CH_2_Cl_2_^d^	23	100 (90)^e^	
14^i^	*t*-Bu_3_PAuNTf_2_	(CH_3_)_3_C	CH_2_Cl_2_^d^	40^j^	100 (64)^e^	
15	*t*-Bu_3_PAuNTf_2_	(CH_3_)_3_C	CH_2_Cl_2_^c^	30	100 (62)^e^	
16^i^	*t*-Bu_3_PAuNTf_2_	(CH_3_)_3_C	CH_2_Cl_2_^c^	30^j^	100 (80)^e^	
17	Ph_3_PAuCl/AgOTf	Ph	CH_2_Cl_2_^d^	80	100 (54)^e^	
18	*t*-Bu_3_PAuNTf_2_	Ph	CH_2_Cl_2_^d^	70	100 (57)^e^	

a Reactions carried out by adding a 0.1 M solution of the substrate (0.2 mmol) and DEAD (1 equiv) in CH_2_Cl_2_ to a 0.1 M solution of the catalyst in the same solvent.

b Conversion determined by ^1^H NMR of the crude reaction mixture.

c Distilled over CaH_2_ prior to use.

d Undistilled solvent (declared water content of the lot: 0.01%).

e Yield after chromatography.

f Prepared by adding water (0.3% v/v) to the solution of the catalyst in CH_2_Cl_2_ freshly distilled from CaH_2_.

g Prepared by adding water (0.3% v/v) to the solution of the catalyst in undistilled CH_2_Cl_2_.

h Undistilled solvent.

i Reaction carried out by adding DEAD after the cycloisomerization was complete.

j Time taken from the addition of DEAD.

We next carried out the same experiment in CH_2_Cl_2_ freshly distilled from CaH_2_ (entry 4). The starting material was consumed in 60 min, after which we stopped the reaction to obtain a 1:1 mixture of **12** and *N*-acetylated compound **13**. In the next experiment (entry 5), we added water (0.3% v/v) to the reaction mixture, and similarly to the reaction carried out in undistilled CH_2_Cl_2_, the conversion of the starting material was complete in 35 min to provide compound **12** only (79% yield after chromatography).

The last two experiments, together with those reported in entries 1 and 2, show that water is essential to avoid the formation of the unwanted *N*-acetylated compound **13**. Even the amount of water present in the commercial CH_2_Cl_2_ that we used (without prior distillation over CaH_2_) seems sufficient for this (entries 2 and 3).^31^ Moreover, given the quantitative formation of product **12**, the cycloaddition step must be much faster than hydrolysis of the intermediate acetate **6** which would instead lead to the corresponding unfunctionalized 2-cyclopentenone.^20^ Given the unpredictability of the water content in the commercial solvent,^32^ we decided to add a measured amount of water to the reaction medium even using undistilled CH_2_Cl_2_ (entry 6). These conditions did not affect the reaction rate (100% conversion in 30 min) and, expectedly, provided compound **12** only (95% yield after chromatography). These were the conditions which we later used in the evaluation of the scope of the reaction.

A series of experiments with catalysts obtained by premixing Ph_3_PAuCl (2 mol %) and different silver salts (entries 7–9) were also carried out in undistilled CH_2_Cl_2_ to evaluate other catalytic systems, and in all cases, the starting material was quickly consumed (30 min) to form the target compound in very high yield (86–95%) after chromatography. These experiments show that the presence of residual silver cations in solution does not affect the reaction outcome. The experiment with AgSbF_6_ as the silver salt was repeated in “wet” CH_2_Cl_2_ providing the same results as in the undistilled solvent (entry 10). We also tried two other solvents: With toluene (undistilled) (entry 11), the consumption of the starting material was very slow, the starting material being consumed in 7 h, to nonetheless give **12** in 91% yield after chromatography. With dichloroethane (undistilled) (entry 12), the reaction was slow, too, reaching 83% conversion in 3 h.

A series of experiments were carried out with pivaloyl ester **10** (entries 13–16). The first experiment was carried out as usual in undistilled CH_2_Cl_2_, and after 23 min, we stopped the reaction to obtain cyclopentenone **12** in 90% yield after chromatography. We carried out, with this substrate, the reaction in sequence, too, by first mixing the catalyst and the substrate in CH_2_Cl_2_, and when the cycloisomerization was complete (10 min), we added DEAD. The TLC spot corresponding to the cycloisomerization product disappeared in 40 min, and after work-up, cyclopentenone **12** was obtained in 64% yield. Interestingly, with this ester as the substrate, the formation of the *N*-acylated byproduct was not observed when carrying out the reaction in anhydrous CH_2_Cl_2_, as after aqueous work-up, we observed the formation of **12** only (entries 15 and 16). Finally, we also tried benzoyl ester **11** as the substrate (entries 17 and 18), but the results were not as satisfactory as those with the previous esters. The cycloisomerization was complete in 10 min with both catalysts, with the formation, in the TLC plate, of a spot probably corresponding to a reaction intermediate which was completely converted into the product in about 1 h. However, ^1^H NMR of the crude reaction mixture revealed the presence of two unidentified byproducts, and **12** was obtained in moderate yield (54–57%) after chromatography.

**Scheme 2:**
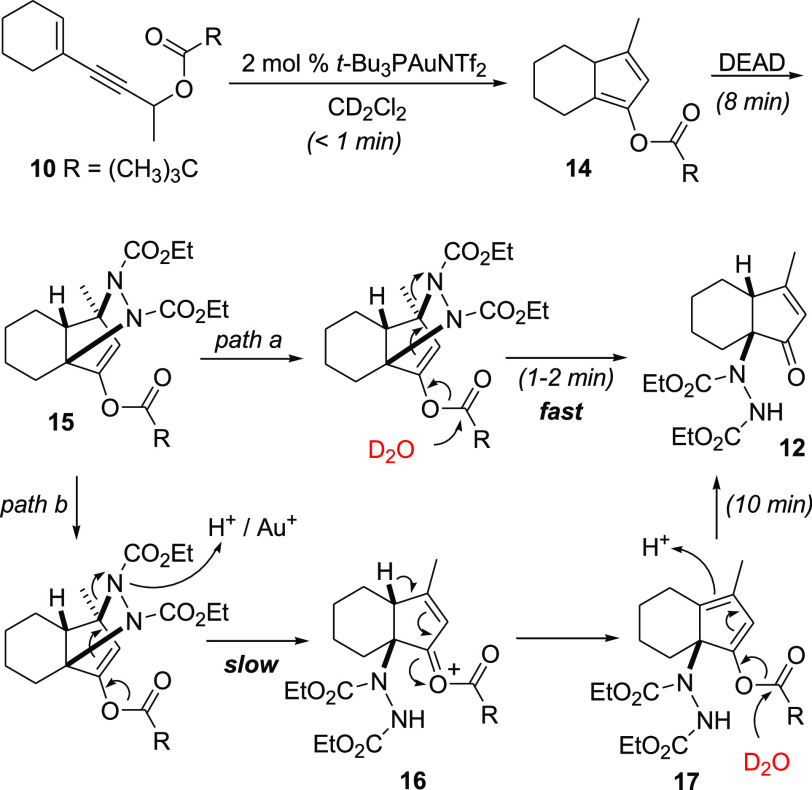
Experiments on Ester 10 in CD_2_Cl_2_ for Direct Monitoring by ^1^H NMR

To have a clear picture of the reaction, we carried out two experiments with pivaloyl ester **10** in CD_2_Cl_2_ (in NMR tubes), monitoring directly by ^1^H NMR (Scheme 2).

We choose **10** to have simpler NMR spectra as this ester does not form the *N*-acylated product in mixture with **12**. In the first experiment (see the Supporting Information), we added the substrate to the solution of the catalyst (2 mol % *t*-Bu_3_PAuNTf_2_) to generate, in less than 1 min, diene **14**,^30^ and to this, we added an excess of DEAD (2 equiv) to initiate the cycloaddition step. The signals of diene **14** completely disappeared after 8 min, and at this point, the signals of two products, in a 3:1 ratio, were visible in the NMR spectrum, i.e., those that we could attribute to cycloadduct **15** (major),^30^ as a single diastereomer, and to compound **17** (minor).^30^ We then added D_2_O (0.3% v/v) which caused the quick transformation (2 min) of cycloadduct **15** into final product **12**, whereas the conversion of minor product **17** into **12** was slower and required 10 min to be completed. After this time, only the signals of our target compound **12** were present in the ^1^H NMR spectrum.

In a similar experiment, we avoided the addition of deuterated water and found that the ratio between compounds **17** and **15** increased during the time, from 1:3 after 8 min to approximately 1.5:1 after 40 min. Thus, in the absence of water, cycloadduct **15** undergoes a slow cleavage of the C–N bond to generate cyclopentadienyl ester **17**, and on the grounds of our previous work with propargyl vinyl ethers,^27^ the ring-opening process leading to **17** could be promoted by the catalyst present, i.e., either by the cationic gold(I) or by the conjugated acid of its counterion (Tf_2_NH). As mentioned, with the substrate we used in the present experiments, we do not observe the formation of the *N*-acylated byproduct under anhydrous conditions. The formation of *N*-acetyl derivative **13** from **9** when working in the absence of water (Table 1, entries 1 and 4) could derive from an intramolecular reaction on either **16** or **17** (when R = Me), whereas with the pivaloyl esters, *N*-acylation is not observed (Table 1, entries 15–16) as it could be impeded by steric hindrance.

Based on the results of the above-mentioned experiments, we may infer that when the reaction is carried out under the optimized conditions, i.e., in the presence of water, the major pathway must involve the hydrolysis of the ester group directly in the cycloadduct **15** as soon as this is formed, which triggers the regioselective cleavage of the C_1_–N_8_ bond by a retro aza-Michael addition driven by the formation of a conjugated system (path a, Scheme 2).

**Scheme 3:**
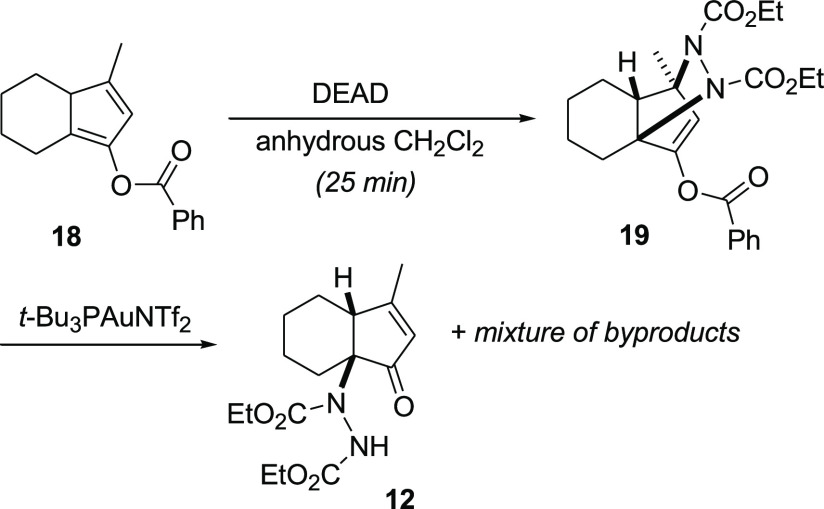
Isolation of the Cycloadduct Intermediate

To gain insights into the role of the gold catalyst in the cycloaddition step and try to isolate the cycloadduct intermediate, we carried out an experiment on known diene **18** (Scheme 3).^15^ By adding DEAD to a solution of **18** in anhydrous CH_2_Cl_2_ and monitoring by TLC, we observed the complete disappearance of the starting material in 25 min, with the formation of cycloadduct **19**,^30^ of which we managed to record an ^1^H NMR spectrum (which showed the presence of a single diastereomer) and an electrospray ionization-mass spectrometry spectrum by directly concentrating a small volume of the reaction mixture.^33^ This experiment thus suggests that the gold(I) catalyst has no role in activating either the diene or the heterodienophile for the cycloaddition step. Instead, the addition of the gold catalyst to the solution of **19** caused the acid-catalyzed C–N ring cleavage to form **12** reasonably according to path b (Scheme 2) but in mixture with unidentified byproducts.

For the evaluation of the scope of the reaction, we screened a few heterodienophiles (DEAD, DIAD, and dibenzyl azodicarboxylate) and propargyl acetates bearing different substituents and distal carbo- and heterocyclic rings (Table 2). In most cases, we observed by TLC the quick disappearance (30 min) of the starting material with the concurrent formation of the desired products (obtained in 76–96% yield after chromatography) when the reaction was carried out in wet dichloromethane (DCM). In two cases only, the reaction was troublesome: (a) When using dibenzyl azodicarboxylate as the heterodienophile (with substrate **9**), we noticed a fast decomposition of the heterodienophile during the reaction. This slowed the cycloaddition step, consequently allowing the hydrolysis of the intermediate acetate before the HDA process and lowering the yield of **29**. (b) With phenyl-substituted substrate **22**, because of a slower cycloaddition step, the hydrolysis of the intermediate acetate occurred in part, too, using both DEAD and diisopropyl azodicarboxylate (DIAD) as heterodienophiles.

**Table 2:**
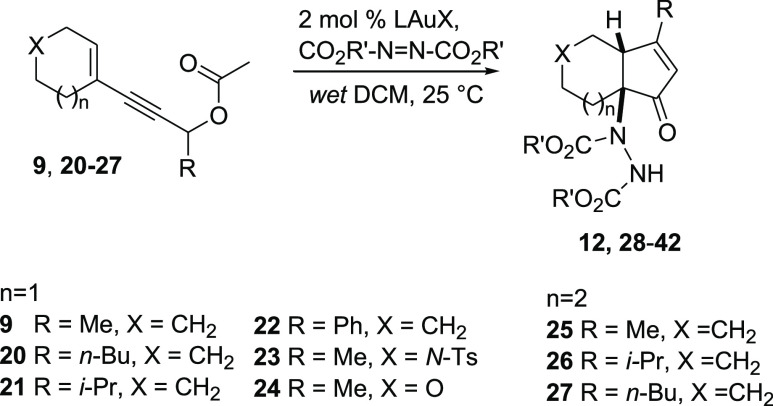

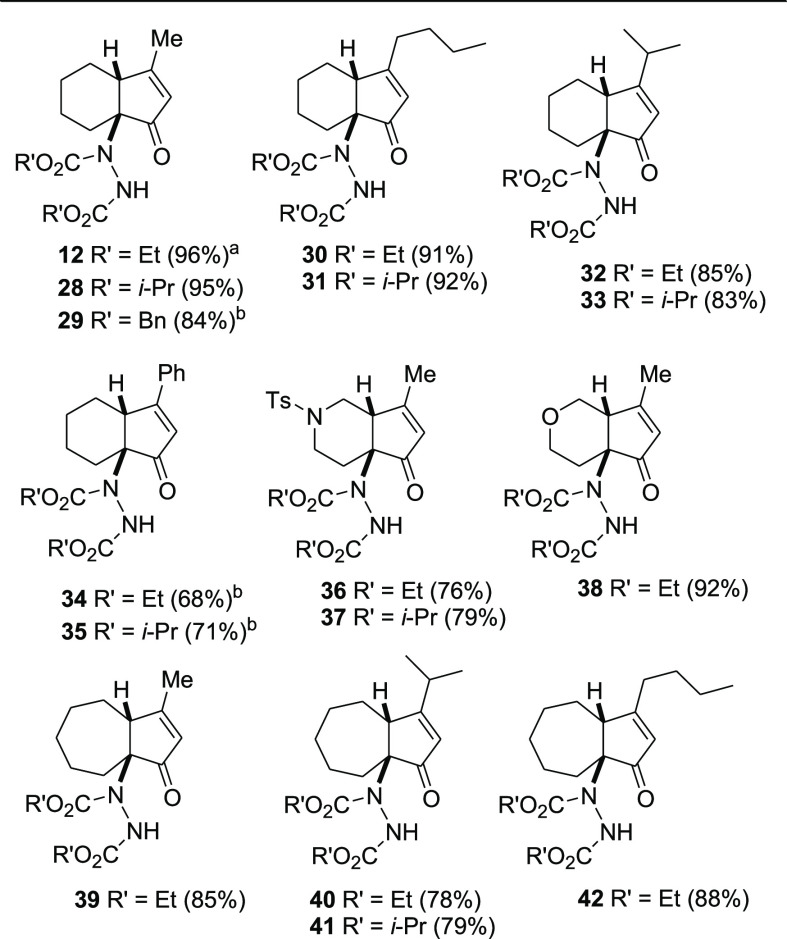
Substrate Scope of the Optimized Process

a Reaction carried out in 1.3 mmol.

b Reaction carried out in undistilled CH_2_Cl_2_ without addition of water.

We found that in these problematic cases, the reaction was best carried out in undistilled CH_2_Cl_2_ without addition of water so that final products **29** (84%) and **34–35** (68–71%) could be obtained in good yield. With substrates bearing a seven-membered ring (**25–27**), the tandem reaction occurred as usual in about 30 min, and the target products (**39–42**) were obtained in very good yield (78–88%). In these cases, however, we observed the formation of a minor diastereomer (12–15%) likely due to a lower facial selectivity in the HDA step, as previously observed with other dienophiles.^26^

To obtain functionalized cyclopentenones fused with a piperidine and a tetrahydropyran ring, the reaction was carried out on substrates **23** and **24**, respectively. With ester **23**, the reaction was carried out with both DEAD and DIAD, providing products **36** and **37** in 76 and 79% yields, respectively. With this substrate, the initial gold-catalyzed rearrangement was slower (about 4 h) than that with the corresponding carbocyclic systems, whereas tetrahydropyran derivative **24** reacted much faster (both rearrangement and cycloaddition/C–N cleavage steps) and, again, with complete facial selectivity to provide **38** in 92% yield.

**Scheme 4:**
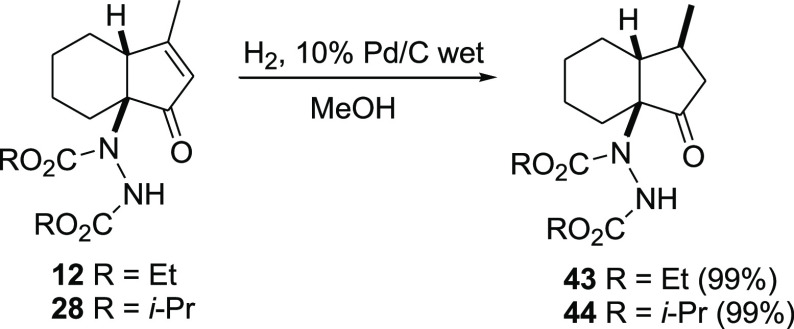
Hydrogenation of Compounds 12 and 28

Finally, in view of the possible use of these cyclopentenones as intermediates in synthesis, we evaluated on two of these compounds (**12** and **28**, Scheme 4) the facial selectivity in reactions involving the α,β-unsaturated ketone moiety.

We choose a simple double bond reduction which was best carried out with both wet Pd/C (10%) as the catalyst in methanol and PtO_2_ in acetic acid, quantitatively providing compounds **43** and **44**, possessing three contiguous stereocenters, with very high facial selectivity. Nuclear Overhauser effect (NOE) studies^34^ revealed that it is the *N*-protected hydrazine appendage that exerted the major hindrance as the addition of hydrogen occurred on the opposite side.

## Conclusions

In conclusion, we have established a robust method for the synthesis of functionalized 2-cyclopentenones by trapping with dialkylazodicarboxylates the dienyl acetate intermediates which are formed in the gold(I)-catalyzed rearrangement of suitable propargyl acetates and the consequent highly regioselective ring opening of the HDA cycloadducts. The presence of the right amount of water is essential to promote the latter step which occurs via a retro aza-Michael reaction and to avoid the formation of the *N*-acylated byproduct. This tandem, one-pot process, which includes a sequence of four reactions (1,3-acyloxy migration, Nazarov cyclization, HDA, and retro aza-Michael addition), provides in high yields (68–96%) unprecedented 5-hydrazino-2-cyclopentenone derivatives with an *N*-substituted quaternary center. Further elaboration of these products and the extension of the methodology to different classes of propargyl esters are currently being evaluated in our laboratories.

## Experimental Section

### General Experimental Methods

Anhydrous solvents were prepared according to the standard techniques. Commercially available reagents were used without further purification. Melting points were recorded on a Büchi B-540 apparatus and are uncorrected. Chromatographic separations were performed under pressure on silica gel (Merck 70–230 mesh) by using flash column techniques; *R*_f_ values refer to TLC carried out on 0.25 mm silica gel plates (F_254_) with the same eluent as that indicated for column chromatography. ^1^H NMR (200 or 400 MHz) and ^13^C{^1^H} NMR (100.4 MHz) spectra were recorded either on Varian Inova (400 MHz) or Mercury (200 or 400 MHz) spectrometers in the specified deuterated solvent at 25 °C. Solvent reference lines were set at 7.26 and 77.00 (CDCl_3_) in ^1^H and ^13^C{^1^H} NMR spectra, respectively. Mass spectra were recorded by direct inlet of a 20 ppm solution in CH_3_OH on an LCQ Fleet Ion Trap LC/MS system (Thermo Fisher Scientific) with an ESI interface in the positive ion mode. Microanalyses were carried out with a Thermo Scientific FlashSmart Elemental Analyzer CHNS/O. Acetates **9**(35) and **22**(12) are known.

### General Procedure for the Synthesis of the Propargyl Acetates

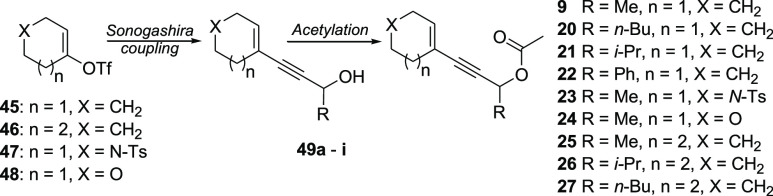



Triflate **45** is commercially available; triflates **46**,^36^**47**,^37^ and **48**(38) were prepared as reported. Propargyl alcohols **49a**,^23^**49b**,^23^**49c**,^26^**49d**,^39^**49g**,^23^**49h**,^26^ and **49i**(26) are known.

#### STEP 1: Sonogashira Coupling

A 3:1 (v/v) solution of anhydrous THF/Et_3_N (6.6 mL, 0.15 M) was added to a round-bottomed flask containing triflates **45**–**48** (1 mmol). The alkynol (1.0–1.1 mmol; 1.0–1.1 equiv), CuI (32 μmol, 3.2 mol %), and (Ph_3_P)_2_PdCl_2_ (16 μmol, 1.6 mol %) were then added under a nitrogen atmosphere, and the reaction mixture was stirred at room temperature for 3 h. Water (25 mL) was then added, and the product was extracted with Et_2_O (3 × 20 mL). The combined organic extracts were washed with brine (50 mL) and dried over anhydrous K_2_CO_3_. After filtration and evaporation of the solvent, the crude reaction mixture was purified by flash chromatography affording the corresponding intermediate propargyl alcohol **49a** – **i**, which was used immediately in the next step.

#### STEP 2: Acetylation

Propargyl alcohol **49a** – **i** (1 mmol) was dissolved in anhydrous DCM (10 mL, 0.1 M), and freshly distilled Et_3_N (3.0 mmol, 3.0 equiv) and a catalytic amount of 4-dimethylaminopyridine (0.05 mmol, 0.05 equiv) were added. After cooling at 0 °C (ice bath), Ac_2_O (2.0 mmol, 2.0 equiv) was dropwise added. After 10 min, the ice bath was removed, and the reaction mixture was left under stirring at 25 °C (external bath) overnight. Aqueous saturated NaHCO_3_ (10 mL) was added, and the product was extracted with DCM (2 × 10 mL); the combined organic extracts were dried over anhydrous K_2_CO_3_. After filtration and evaporation of the solvent, the crude reaction mixture was purified by flash column chromatography to give the pure propargyl acetate which was stored at 4 °C as a solution in the eluent containing 1% Et_3_N. The solution of the propargyl acetate in the eluent was concentrated and dried under *vacuum* just prior to use.

#### 2,2-Dimethylpropionic Acid 3-Cyclohex-1-enyl-1-methylprop-2-ynyl Ester (**10**)

Compound **10** was prepared starting from propargyl alcohol **49a** (207 mg, 1.4 mmol) and following the general acetylation procedure but using pivaloyl chloride (204 μL, 1.7 mmol) as the acylating agent and an excess of Et_3_N (1.9 mL, 14 mmol). Purification by flash chromatography (*n*-hexane/EtOAc, 30:1 + 1% Et_3_N; *R*_f_ = 0.29) afforded pure **10** as a colorless oil (277 mg, 86%). ^1^H NMR (400 MHz, CDCl_3_): δ 6.11–6.09 (m, 1H), 5.55 (q, *J* = 6.8 Hz, 1H), 2.12–2.04 (m, 4H), 1.64–1.53 (m, 4H), 1.46 (d, *J* = 6.4 Hz, 3H), 1.20 (s, 9H). ^13^C{^1^H} NMR (100.4 MHz, CDCl_3_): δ 177.3, 135.5, 120.0, 86.0, 85.0, 60.7, 38.6, 29.0, 27.0 (3 C), 25.6, 22.2, 21.5, 21.4. MS (ESI) *m/z* (%): 257 ([M + Na]^+^, 100). IR (CHCl_3_): 3026, 2975, 2938, 2862, 2225, 1734, 1723, 1281, 1158 cm^–1^. Anal. Calcd for C_15_H_22_O_2_: C, 76.88; H, 9.46. Found: C, 76.65; H, 9.45.

#### Acetic Acid 3-Cyclohex-1-enyl-1-butylprop-2-ynyl Ester (**20**)

Propargyl alcohol **49b** was prepared as reported.^23^ Acetylation of compound **49b** (143 mg, 0.75 mmol) afforded **20**, which was purified by flash chromatography (*n*-hexane/EtOAc, 30:1 + 1% Et_3_N; *R*_f_ = 0.43). Pure **20** was obtained as a colorless oil (153 mg, 88%). ^1^H NMR (400 MHz, CDCl_3_): δ 6.11–6.09 (m, 1H), 5.46 (t, *J* = 6.4 Hz, 1H), 2.11–2.04 (m, 4H), 2.05 (s, 3H), 1.77–1.70 (m, 2H), 1.60–1.55 (m, 4H), 1.41–1.29 (m, 4H), 0.89 (t, *J* = 7.2 Hz, 3H). ^13^C{^1^H} NMR (100.4 MHz, CDCl_3_): δ 169.9, 135.7, 119.9, 86.9, 83.8, 64.6, 34.7, 29.0, 27.1, 25.5, 22.2, 22.1, 21.4, 21.0, 13.9. MS (ESI) *m/z* (%): 257 ([M + Na]^+^, 100). Anal. Calcd for C_15_H_22_O_2_: C, 76.88; H, 9.46. Found: C, 76.95; H, 9.41.

#### Acetic Acid 3-Cyclohex-1-enyl-1-isopropylprop-2-ynyl Ester (**21**)

Propargyl alcohol **49c** was prepared as reported.^26^ Acetylation of propargyl alcohol **49c** (133 mg, 0.74 mmol) afforded **21**, which was purified by flash chromatography (*n*-hexane/EtOAc, 40:1 + 1% Et_3_N; R_*f*_ = 0.17). Pure **21** was obtained as a colorless oil (112 mg, 69%). ^1^H NMR (400 MHz, CDCl_3_): δ 6.13–6.09 (m, 1H), 5.32 (d, *J* = 5.2 Hz, 1H), 2.12–2.03 (m, 4H), 2.06 (s, 3H), 2.01–1.93 (m, 1H), 1.63–1.53 (m, 4H), 0.99 (d, *J* = 6.8 Hz, 3H), 0.97 (d, *J* = 6.8 Hz, 3H). ^13^C{^1^H} NMR (100.4 MHz, CDCl_3_): δ 170.0, 135.7, 119.9, 87.6, 82.4, 69.5, 32.5, 29.0, 25.5, 22.1, 21.4, 21.0, 18.2, 17.5. MS (ESI) *m/z* (%): 243 ([M + Na]^+^, 100). IR (CHCl_3_): 3027, 2933, 2876, 2223, 1734, 1373, 1242 cm^–1^. Anal. Calcd for C_14_H_20_O_2_: C, 76.33; H, 9.15. Found: C, 76.51; H, 9.30.

#### Acetic Acid 1-Methyl-3-(1-tosyl-1,2,3,6-tetrahydropyridin-4-yl)-prop-2-ynyl Ester (**23**)

Compound **49e** was obtained by Sonogashira coupling of **47** (1.13 g, 2.9 mmol) and (±)-3-butyn-2-ol (230 μL, 2.9 mmol). Purification of the crude by flash chromatography (*n*-hexane/EtOAc, 2:1 + 1% Et_3_N; *R*_f_ = 0.13) afforded pure propargyl alcohol **49e** which was used immediately in the next step. ^1^H NMR (400 MHz, CDCl_3_): δ 7.66 (d, *J* = 8.0 Hz, 2H), 7.32 (d, *J* = 8.0 Hz, 2H), 5.94–5.92 (m, 1H), 4.61 (q, *J* = 6.8 Hz, 1H), 3.65–3.62 (m, 2H), 3.16 (t, *J* = 5.6 Hz, 2H), 2.43 (s, 3H), 2.32–2.28 (m, 2H), 1.44 (d, *J* = 6.4 Hz, 3H). Acetylation of compound **49e** afforded **23**, which was purified by flash chromatography (*n*-hexane/EtOAc, 4:1 + 1% Et_3_N; *R*_f_ = 0.29). Pure **23** was obtained as a thick yellow oil (572 mg, 56% over 2 steps from **47**). ^1^H NMR (400 MHz, CDCl_3_): δ 7.63 (d, *J* = 8.4 Hz, 2H), 7.30 (d, *J* = 8.0 Hz, 2H), 5.95–5.93 (m, 1H), 5.50 (q, *J* = 6.8 Hz, 1H), 3.62–3.59 (m, 2H), 3.14 (t, *J* = 6.0 Hz, 2H), 2.41 (s, 3H), 2.30–2.26 (m, 2H), 2.04 (s, 3H), 1.43 (d, *J* = 6.4 Hz, 3H). ^13^C{^1^H} NMR (100.4 MHz, CDCl_3_): δ 169.7, 143.7, 132.9, 129.7 (2 C), 129.4, 127.6 (2 C), 118.5, 87.1, 83.7, 60.5, 44.8, 42.3, 29.1, 21.4, 21.3, 21.0. MS (ESI) *m/z* (%): 370 ([M + Na]^+^, 100). IR (CHCl_3_): 3028, 3014, 2940, 2858, 2232, 1734, 1343, 1230 cm^–1^. Anal. Calcd for C_18_H_21_NO_4_S: C, 62.23; H, 6.09; N, 4.03; S, 9.23. Found: C, 62.15; H, 6.19; N, 3.80; S, 8.90.

#### Acetic Acid 3-(3,6-Dihydro-2H-pyran-4-yl)-1-methylprop-2-ynyl Ester (**24**)

Compound **49f** was obtained by Sonogashira coupling of **48** (2.0 mmol) and (±)-3-butyn-1-ol (157 μL, 2.0 mmol). Purification of the crude by flash chromatography (*n*-hexane/EtOAc, 3:1 + 1% Et_3_N; *R*_f_ = 0.22) afforded pure propargyl alcohol **49f** which was used immediately in the next step. ^1^H NMR (200 MHz, CDCl_3_): δ 6.07–6.04 (m, 1H), 4.65 (q, *J* = 6.8 Hz, 1H), 4.21–4.18 (m, 2H), 3.77 (t, *J* = 5.4 Hz, 2H), 2.27–2.19 (m, 2H), 1.47 (d, *J* = 6.4 Hz, 3H). Acetylation of compound **49f** afforded **24**, which was purified by flash chromatography (*n*-hexane/EtOAc, 30:1 + 1% Et_3_N; *R*_f_ = 0.24). Pure **24** was obtained as a colorless oil (266 mg, 69% over 2 steps from **S7**). ^1^H NMR (400 MHz, CDCl_3_): δ 6.13–6.06 (m, 1H), 5.57 (q, *J* = 6.8 Hz, 1H), 4.22–4.12 (m, 2H), 3.76 (t, *J* = 5.6 Hz, 2H), 2.26–2.16 (m, 2H), 2.07 (s, 3H), 1.50 (d, *J* = 6.4 Hz, 3H). ^13^C{^1^H} NMR (100.4 MHz, CDCl_3_): δ 169.8, 133.3, 117.6, 86.4, 84.2, 65.2, 63.7, 60.6, 28.9, 21.4, 21.0. MS (ESI) *m/z* (%): 217 ([M + Na]^+^, 100). IR (CHCl_3_): 3028, 2937, 2863, 2832, 2230, 1734, 1238 cm^–1^. Anal. Calcd for C_11_H_14_O_3_: C, 68.02; H, 7.27. Found: C, 67.95; H, 7.35.

#### Acetic Acid 3-Cyclohept-1-enyl-1-methylprop-2-ynyl Ester (**25**)

Propargyl alcohol **49g** was prepared as reported.^23^ Acetylation of compound **49g** (63 mg, 0.38 mmol) afforded **25**, which was purified by flash chromatography (*n*-hexane/EtOAc, 40:1 + 1% Et_3_N; *R*_f_ = 0.29). Pure **25** was obtained as a colorless oil (54 mg, 69%). ^1^H NMR (400 MHz, CDCl_3_): δ 6.31 (t, *J* = 6.4 Hz, 1H), 5.58 (q, *J* = 6.4 Hz, 1H), 2.32–2.29 (m, 2H), 2.19–2.15 (m, 2H), 2.07 (s, 3H), 1.76–1.69 (m, 2H), 1.58–1.52 (m, 3H), 1.51–1.47 (m, 2H), 1.48 (d, *J* = 6.4 Hz, 3H). ^13^C{^1^H} NMR (100.4 MHz, CDCl_3_): δ 169.9, 141.0, 125.9, 88.0, 84.5, 61.0, 34.0, 32.0, 29.1, 26.5, 26.4, 21.6, 21.1. MS (ESI) *m/z* (%): 229 ([M + Na]^+^, 100). IR (CHCl_3_): 3020, 2927, 2853, 2221, 1733, 1449, 1372, 1233 cm^–1^. Anal. Calcd for C_13_H_18_O_2_: C, 75.69; H, 8.80. Found: C, 75.70; H, 8.80.

#### Acetic Acid 3-Cyclohept-1-enyl-1-isopropylprop-2-ynyl Ester (**26**)

Propargyl alcohol **49h** was prepared as reported.^26^ Acetylation of compound **49h** (260 mg, 1.35 mmol) afforded **26**, which was purified by flash chromatography (*n*-hexane/EtOAc, 30:1 + 1% Et_3_N; R_*f*_ = 0.28). Pure **26** was obtained as a pale-yellow oil (297 mg, 94%). ^1^H NMR (400 MHz, CDCl_3_): δ 6.30 (t, *J* = 6.4 Hz, 1H), 5.35 (d, *J* = 5.6 Hz, 1H), 2.33–2.29 (m, 2H), 2.19–2.14 (m, 2H), 2.08 (s, 3H), 2.03–1.94 (m, 1H), 1.76–1.69 (m, 2H), 1.58–1.47 (m, 4H), 1.01 (d, *J* = 6.8 Hz, 3H), 0.98 (d, *J* = 6.8 Hz, 3H). ^13^C{^1^H} NMR (100.4 MHz, CDCl_3_): δ 170.0, 140.7, 126.0, 89.2, 82.2, 69.6, 34.0, 32.6, 32.0, 29.1, 26.44, 26.37, 21.0, 18.2, 17.5. MS (ESI) *m/z* (%): 257 ([M + Na]^+^, 100). IR (CHCl_3_): 3032, 2969, 2927, 2854, 2213, 1733, 1448, 1372, 1236 cm^–1^. Anal. Calcd for C_15_H_22_O_2_: C, 76.88; H, 9.46. Found: C, 76.90; H, 9.46.

#### Acetic Acid 3-Cyclohept-1-enyl-1-butylprop-2-ynyl Ester (**27**)

Propargyl alcohol **49i** was prepared as reported.^26^ Acetylation of compound **49i** (323 mg, 1.57 mmol) afforded **27**, which was purified by flash chromatography (*n*-hexane/EtOAc, 30:1 + 1% Et_3_N; R_*f*_ = 0.45). Pure **27** was obtained as a colorless oil (366 mg, 94%). ^1^H NMR (400 MHz, CDCl_3_): δ 6.30 (t, *J* = 6.8 Hz, 1H), 5.48 (t, *J* = 6.8 Hz, 1H), 2.32–2.28 (m, 2H), 2.19–2.14 (m, 2H), 1.78–1.69 (m, 4H), 1.57–1.46 (m, 4H), 1.42–1.31 (m, 4H), 0.91 (t, *J* = 6.4 Hz, 3H). ^13^C{^1^H} NMR (100.4 MHz, CDCl_3_): δ 170.0, 140.8, 126.0, 88.6, 83.7, 64.7, 34.7, 34.0, 32.0, 29.1, 27.2, 26.42, 26.35, 22.2, 21.1, 13.9. MS (ESI) *m/z* (%): 519 ([2 M + Na]^+^, 42), 271 ([M + Na]^+^, 100). IR (CHCl_3_): 3027, 2929, 2855, 2216, 1734, 1457, 1374, 1230 cm^–1^. Anal. Calcd for C_16_H_24_O_2_: C, 77.38; H, 9.74. Found: C, 77.40; H, 9.79.

### General Procedure for the Gold(I)-Catalyzed Cycloisomerization/Hetero-Diels–Alder/Ring-Opening Tandem Reaction

The solution of propargyl acetates **9** and **20**–**27** in the eluent used for chromatography was concentrated and dried under *vacuum* just prior to use. Water (0.7 mmol, 3.5 equiv) was added to a solution of commercially available gold(I) complex ^*t*^Bu_3_PAuNTf_2_ (4.0 μmol, 2 mol %) in DCM (2 mL) and stirred at 25 °C under a nitrogen atmosphere, followed by the addition of a solution of propargyl acetate (0.2 mmol) and the dienophile (0.2 mmol, 1.0 equiv) in DCM (2 mL; final concentration of the acetate: 0.05 M). The reaction mixture was stirred at 25 °C until complete consumption of the starting material (TLC monitoring; usually 0.5–4 h). Aqueous saturated NaHCO_3_ (4 mL) was added, and the reaction mixture was vigorously stirred at 25 °C for 20 min; after separation of the phases, the product was extracted with DCM (5 mL), and the combined organic extracts were dried over anhydrous Na_2_SO_4_. After filtration and evaporation of the solvent, the oily residue was purified by flash chromatography.

#### Diethyl (3aS*,7aR*)-1-(1-Methyl-3-oxo-3,4,5,6,7,7a-hexahydro-3aH-inden-3a-yl)-hydrazine 1,2-Dicarboxylate (**12**)

Compound **12** was prepared following the general procedure, starting from acetate **9** (52 mg, 0.27 mmol) and DEAD. The reaction was complete in 30 min. Purification by flash chromatography (EtOAc/*n*-hexane, 1:2; R_*f*_ = 0.20) afforded **12** (83 mg, 95%) as a white solid. mp 98.9–100.1 °C. ^1^H NMR (400 MHz, CDCl_3_) (3.3 : 1 mixture of rotamers): δ 6.70 (br s, 1H, major), 6.47 (br s, 1H, minor), 5.98 (br s, 1H), 4.29–4.19 (m, 2H), 4.18–4.06 (m, 2H), 3.40 (br s, 1H, major), 3.33 (br s, 1H, minor), 2.09 (s, 3H), 2.01–1.91 (m, 3H), 1.64–1.56 (m, 1H), 1.53–1.42 (m, 2H), 1.39–1.25 (m, 1H), 1.30 (t, *J* = 6.8 Hz, 3H), 1.19 (t, *J* = 6.8 Hz, 3H), 1.14–1.06 (m, 1H). ^13^C{^1^H} NMR (100.4 MHz, CDCl_3_) (mixture of rotamers): δ 206.6 and 206.2, 176.8 and 176.2, 156.8 and 156.6, 154.8 and 154.7, 127.6 and 127.3, 68.6, 62.4 and 62.2, 62.0, 48.1 and 47.8, 29.2 and 29.0, 21.7 and 21.6, 20.3 and 20.1, 20.0 and 19.8, 17.3, 14.42 and 14.36, 14.3 and 14.1. MS (ESI) *m/z* (%): 671 ([2M+ Na]^+^, 70), 347 ([M + Na]^+^, 100). IR (CHCl_3_): 3393, 3027, 2943, 1749, 1714, 1617, 1379, 1229, 1203 cm^–1^. Anal. Calcd for C_16_H_24_N_2_O_5_: C, 59.24; H, 7.46; N, 8.64. Found: C, 59.53; H, 7.66; N, 8.36. On carrying out the reaction under the conditions reported in Table 1 entry 4 (see text), a 1:1 mixture of compounds **12** and **13** was obtained. A small amount of compound **13** could be isolated by flash chromatography (eluent: EtOAc/*n*-hexane, 1:4 + 1% Et_3_N; *R*_*f*_ = 0.24) and spectroscopically characterized. **13**: ^1^H NMR (400 MHz, CDCl_3_) (3:1 mixture of rotamers): δ 6.00 (m, 1H, minor), 5.97 (m, 1H, major), 4.37–4.01 (m, 4H), 3.36 (br s, 1H, major), 3.28 (br s, 1H, minor), 2.57 (s, 3H, minor), 2.50 (s, 3H, major), 2.09 (s, 3H, major), 2.07 (s, 3H, minor), 2.04–1.93 (m, 1H), 1.87–1.82 (m, 1H), 1.74–1.65 (m, 2H), 1.55–1.46 (m, 1H), 1.43–1.36 (m, 3H), 1.30–1.22 (m, 2H), 1.20–1.08 (m, 4H). ^13^C{^1^H} NMR (100.4 MHz, CDCl_3_) (mixture of rotamers, major rotamer reported): δ 205.1, 179.7, 171.6, 153.7, 153.4, 129.1, 69.8, 64.0, 62.5, 49.7, 27.2, 24.4, 21.2, 17.6, 17.4, 17.3, 14.4, 14.1. MS (ESI) *m/z* (%): 755 ([2 M + Na]^+^, 100), 389 ([M + Na]^+^, 33), 367 ([M + 1]^+^, 2). IR (CHCl_3_): 3033, 2985, 2943, 1717, 1623, 1377, 1336, 1259 cm^–1^. Compound **12** was also synthesized on a larger scale, starting from acetate **9** (250 mg, 1.3 mmol) and affording, after purification by flash chromatography, pure **12** (404 mg, 96%) as a white solid.

#### Diisopropyl (3aS*,7aR*)-1-(1-Methyl-3-oxo-3,4,5,6,7,7a-hexahydro-3aH-inden-3a-yl)-hydrazine 1,2-Dicarboxylate (**28**)

Compound **28** was prepared following the general procedure, starting from acetate **9** (53 mg, 0.27 mmol) and DIAD. The reaction was complete in 30 min. Purification by flash chromatography (EtOAc/*n*-hexane, 1:3; *R*_*f*_ = 0.14) afforded **28** (90 mg, 95%) as a white solid. mp 132.6–134.0 °C. ^1^H NMR (400 MHz, CDCl_3_) (2.9 : 1 mixture of rotamers): δ 6.66 (br s, 1H, major), 6.48 (br s, 1H, minor), 5.99 (s, 1H, minor), 5.97 (s, 1H, major), 5.04–4.91 (m, 1H), 4.87–4.80 (m, 1H), 3.39 (br s, 1H, major), 3.31 (br s, 1H, minor), 2.07 (s, 3H), 1.98–1.92 (m, 3H), 1.61–1.56 (m, 1H), 1.50–1.40 (m, 2H), 1.32–1.25 (m, 1H), 1.28 (d, *J* = 6.0 Hz, 3H), 1.27 (d, *J* = 6.0 Hz, 3H), 1.17 (d, *J* = 6.4 Hz, 3H), 1.15 (d, *J* = 6.4 Hz, 3H), 1.11–1.06 (m, 1H). ^13^C{^1^H} NMR (100.4 MHz, CDCl_3_) (mixture of rotamers): δ 206.7, 176.8 and 176.2, 156.6, 154.4, 127.8 and 127.4, 70.4 and 70.2, 69.9, 68.7 and 68.4, 48.3 and 47.9, 29.3 and 29.0, 21.95 (2 C), 21.89, 21.86, 21.7, 20.3, 20.1, 17.3. MS (ESI) *m/z* (%): 727 ([2 M + Na]^+^, 100), 375 ([M + Na]^+^, 39). IR (CHCl_3_): 3397, 3031, 2985, 2941, 1734, 1710, 1376, 1240 cm^–1^. Anal. Calcd for C_18_H_28_N_2_O_5_: C, 61.34; H, 8.01; N, 7.95. Found: C, 61.39; H, 8.08; N, 7.47.

#### Dibenzyl (3aS*,7aR*)-1-(1-Methyl-3-oxo-3,4,5,6,7,7a-hexahydro-3aH-inden-3a-yl)-hydrazine 1,2-Dicarboxylate (**29**)

The solution of propargyl acetate **9** in EtOAc/*n*-hexane and 1:20 + 1% Et_3_N was concentrated and dried under *vacuum* just prior to use. Gold(I) complex IPrAuSbF_6_ was generated in situ by mixing IPrAuCl (2.4 mg, 4.9 μmol, 2 mol %) and AgSbF_6_ (1.7 mg, 4.9 μmol, 2 mol %) in DCM (2.5 mL) and leaving the mixture under stirring for 5 min at 25 °C before adding the substrates. In a round bottom flask containing acetate **9** (47 mg, 0.24 mmol) and dibenzyl azodicarboxylate (80 mg, 0.27 mmol), DCM (2.4 mL) was added, and the resulting solution was immediately transferred into the flask containing the gold(I) complex. The reaction mixture was stirred until complete consumption of the starting material (1.75 h). Aqueous saturated NaHCO_3_ (5 mL) was added, and the reaction mixture was vigorously stirred at 25 °C for 20 min; after separation of the phases, the product was extracted with DCM (5 mL), and the combined organic extracts were dried over anhydrous Na_2_SO_4_. After filtration and evaporation of the solvent, the oily residue was purified by flash chromatography (eluent: EtOAc/*n*-hexane, 1:2; *R*_*f*_ = 0.32), affording pure **29** (90 mg, 84%) as a white foam. ^1^H NMR (400 MHz, CDCl_3_) (2.5 : 1 mixture of rotamers): δ 7.32–7.29 (m, 8H), 7.24–7.21 (m, 2H), 6.96 (br s, 1H, major), 6.71 (br s, 1H, minor), 5.95 (s, 1H), 5.19–4.96 (m, 4H), 3.41 (m, 1H, major), 3.17 (m, 1H, minor), 2.04 (s, 3H, major), 1.97–1.93 (m, 3H and 3H minor), 1.61–1.56 (m, 1H), 1.51–1.37 (m, 2H), 1.32–1.26 (m, 1H), 1.14–1.04 (m, 1H). ^13^C{^1^H} NMR (100.4 MHz, CDCl_3_) (mixture of rotamers): δ 206.5 and 206.3, 177.1 and 176.6, 156.7 and 156.4, 154.7 and 154.6, 135.6 and 135.4 (2 C), 128.5 (2 C), 128.32 (2 C), 128.29 (2 C), 128.0 (2 C), 127.8 (2 C), 127.4, 68.9 and 68.8, 68.1 and 68.0, 67.7 and 67.6, 48.1 and 47.7, 29.2 and 28.9, 21.7 and 21.3, 20.3, 20.0 and 19.6, 17.3. MS (ESI) *m/z* (%): 919 ([2 M + Na]^+^, 100), 471 ([M + Na]^+^, 45), 449 ([M + 1]^+^, 25). IR (CHCl_3_): 3395, 3029, 2945, 1749, 1717, 1617, 1233 cm^–1^. Anal. Calcd for C_26_H_28_N_2_O_5_: C, 69.63; H, 6.29; N, 6.25. Found: C, 69.56; H, 6.31; N, 6.28.

#### Diethyl (3aS*,7aR*)-1-(1-Butyl-3-oxo-3,4,5,6,7,7a-hexahydro-3aH-inden-3a-yl)-hydrazine 1,2-Dicarboxylate (**30**)

Compound **30** was prepared following the general procedure, starting from acetate **20** (42 mg, 0.18 mmol) and DEAD. The reaction was complete in 30 min. Purification by flash chromatography (EtOAc/*n*-hexane, 1:2; *R*_*f*_ = 0.29) afforded **30** (60 mg, 91%) as a white solid. mp 70.5–73.0 °C. ^1^H NMR (400 MHz, CDCl_3_) (3.1 : 1 mixture of rotamers): δ 7.04 (br s, 1H, major), 6.81 (br s, 1H, minor), 5.93 (s, 1H), 4.22–4.13 (m, 2H), 4.11–3.99 (m, 2H), 3.39 (br s, 1H, major), 3.32 (br s, 1H, minor), 2.39–2.22 (m, 2H), 1.98–1.86 (m, 3H), 1.60–1.48 (m, 3H), 1.48–1.30 (m, 5H), 1.26 (t, *J* = 7.2 Hz, 3H), 1.13 (t, *J* = 6.8 Hz, 3H), 1.10–0.99 (m, 1H), 0.90 (t, *J* = 7.2 Hz, 3H). ^13^C{^1^H} NMR (100.4 MHz, CDCl_3_) (mixture of rotamers): δ 206.8 and 206.5, 181.0 and 180.4, 156.9 and 156.7, 154.8, 125.7 and 125.5, 68.6, 62.4, 62.0, 47.2 and 46.9, 30.7, 29.3 and 29.1, 28.6, 22.4, 21.9 and 21.8, 20.5 and 20.3, 20.2 and 20.0, 14.4, 14.1, 13.7. MS (ESI) *m/z* (%): 755 ([2 M + Na]^+^, 100), 389 ([M + Na]^+^, 64). IR (CHCl_3_): 3398, 3027, 2939, 2874, 1749, 1715, 1379, 1230 cm^–1^. Anal. Calcd for C_19_H_30_N_2_O_5_: C, 62.27; H, 8.25; N, 7.64. Found: C, 62.27; H, 8.34; N, 7.37.

#### Diisopropyl (3aS*,7aR*)-1-(1-Butyl-3-oxo-3,4,5,6,7,7a-hexahydro-3aH-inden-3a-yl)-hydrazine 1,2-Dicarboxylate (**31**)

Compound **31** was prepared following the general procedure, starting from acetate **20** (43 mg, 0.18 mmol) and DIAD. The reaction was complete in 30 min. Purification by flash chromatography (EtOAc/*n*-hexane, 1:2; *R*_*f*_ = 0.33) afforded **31** (67 mg, 92%) as a white solid. mp 82.7–84.5 °C. ^1^H NMR (400 MHz, CDCl_3_) (2.9 : 1 mixture of rotamers): δ 6.59 (br s, 1H, major), 6.38 (br s, 1H, minor), 5.99 (s, 1H, minor), 5.96 (s, 1H, major), 5.03–4.94 (m, 1H), 4.89–4.83 (m, 1H), 3.43 (br s, 1H, major), 3.36 (br s, 1H, minor), 2.42–2.26 (m, 2H), 2.01–1.93 (m, 3H), 1.65–1.55 (m, 3H), 1.49–1.35 (m, 5H), 1.293 (d, *J* = 6.4 Hz, 3H), 1.288 (d, *J* = 6.4 Hz, 3H), 1.18 (d, *J* = 6.4 Hz, 3H), 1.16 (d, *J* = 6.4 Hz, 3H), 1.12–1.07 (m, 1H), 0.94 (t, *J* = 7.6 Hz, 3H). ^13^C{^1^H} NMR (100.4 MHz, CDCl_3_) (mixture of rotamers): δ 206.8, 180.8 and 180.3, 156.7 and 156.5, 154.5, 125.9 and 125.6, 70.4 and 70.2, 69.9, 68.4, 47.4 and 47.1, 30.7, 29.5, 28.7, 22.6, 22.0 (2 C), 21.9, 21.7 and 21.6 (2 C), 20.6, 20.4, 13.8. MS (ESI) *m/z* (%): 811 ([2 M + Na]^+^, 72), 417 ([M + Na]^+^, 100). IR (CHCl_3_): 3397, 3015, 2985, 2939, 2875, 1748, 1707, 1385, 1240 cm^–1^. Anal. Calcd for C_21_H_34_N_2_O_5_: C, 63.93; H, 8.69; N, 7.10. Found: C, 63.90; H, 7.25; N, 8.74.

#### Diethyl (3aS*,7aR*)-1-(1-Isopropyl-3-oxo-3,4,5,6,7,7a-hexahydro-3aH-inden-3a-yl)-hydrazine 1,2-Dicarboxylate (**32**)

Compound **32** was prepared following the general procedure, starting from acetate **21** (41 mg, 0.19 mmol) and DEAD. The reaction was complete in 30 min. Purification by flash chromatography (EtOAc/*n*-hexane, 1:2; *R*_*f*_ = 0.23) afforded **32** (56 mg, 85%) as a white solid. mp 118.5–122.7 °C. ^1^H NMR (400 MHz, CDCl_3_) (4.6 : 1 mixture of rotamers): δ 6.75 (br s, 1H, major), 6.53 (br s, 1H, minor), 5.97 (s, 1H, minor), 5.95 (s, 1H, major), 4.27–4.17 (m, 2H), 4.09 (q, *J* = 7.2 Hz, 2H), 3.51 (m, 1H, major), 3.45 (m, 1H, minor), 2.59 (quint, *J* = 6.8 Hz, 1H), 2.02–1.91 (m, 3H), 1.63–1.57 (m, 1H), 1.53–1.42 (m, 2H), 1.40–1.32 (m, 1H), 1.30 (t, *J* = 6.8 Hz, 3H), 1.21 (d, *J* = 6.8 Hz, 3H), 1.17 (t, *J* = 7.2 Hz, 3H), 1.15 (d, *J* = 6.8 Hz, 3H), 1.18–1.12 (m, 1H). ^13^C{^1^H} NMR (100.4 MHz, CDCl_3_) (mixture of rotamers): δ 207.1 and 206.7, 186.2 and 185.6, 157.0 and 156.7, 154.9, 124.0 and 123.7, 69.0 and 68.9, 62.4 and 62.3, 62.2 and 62.0, 46.2 and 45.8, 29.6 and 29.3, 29.1 and 29.0, 21.9 and 21.8, 21.0, 20.5 and 20.4, 20.2, 20.1 and 19.9, 14.45 and 14.38, 14.3 and 14.2. MS (ESI) *m/z* (%): 727 ([2M + Na]^+^, 100), 375 ([M + Na]^+^, 22). IR (CHCl_3_): 3395, 3031, 2942, 2874, 1749, 1715, 1339, 1233 cm^–1^. Anal. Calcd for C_18_H_28_N_2_O_5_: C, 61.34; H, 8.01; N, 7.95. Found: C, 61.32; H, 8.04; N, 7.91.

#### Diisopropyl (3aS*,7aR*)-1-(1-Isopropyl-3-oxo-3,4,5,6,7,7a-hexahydro-3aH-inden-3a-yl)-hydrazine 1,2-Dicarboxylate (**33**)

Compound **33** was prepared following the general procedure, starting from acetate **21** (43 mg, 0.19 mmol) and DIAD. The reaction was complete in 60 min. Purification by flash chromatography (EtOAc/*n*-hexane, 1:4; *R*_*f*_ = 0.28) afforded **33** (61 mg, 83%) as a white solid. mp 131.0–133.8 °C. ^1^H NMR (400 MHz, CDCl_3_) (3.2 : 1 mixture of rotamers): δ 6.62 (br s, 1H, major), 6.41 (br s, 1H, minor), 5.97 (s, 1H, minor), 5.94 (s, 1H, major), 5.00–4.94 (m, 1H), 4.88–4.82 (m, 1H), 3.51 (br s, 1H, major), 3.44 (br s, 1H, minor), 2.62–2.56 (m, 1H), 2.05–1.88 (m, 3H), 1.63–1.58 (m, 1H), 1.50–1.43 (m, 2H), 1.41–1.33 (m, 1H), 1.29 (d, *J* = 6.4 Hz, 3H), 1.28 (d, *J* = 6.0 Hz, 3H), 1.22 (d, *J* = 6.8 Hz, 3H), 1.18–1.14 (m, 9H), 1.13–1.05 (m, 1H). ^13^C{^1^H} NMR (100.4 MHz, CDCl_3_) (mixture of rotamers): δ 207.0, 185.8 and 185.4, 156.7, 154.4, 124.2 and 123.7, 70.4, 69.8, 68.8, 46.3 and 46.0, 29.5, 29.0, 22.0, 21.92 (2 C), 21.86, 21.7, 21.0, 20.6, 20.3, 20.1. MS (ESI) *m/z* (%): 783 ([2M+ Na]^+^, 100), 403 ([M + Na]^+^, 38). IR (CHCl_3_): 3393, 3031, 2984, 2941, 2876, 1746, 1712, 1385, 1233 cm^–1^. Anal. Calcd for C_20_H_32_N_2_O_5_: C, 63.13; H, 8.48; N, 7.36. Found: C, 63.06; H, 8.59; N, 7.66.

#### Diethyl (3aS*,7aR*)-1-(1-Phenyl-3-oxo-3,4,5,6,7,7a-hexahydro-3aH-inden-3a-yl)-hydrazine 1,2-Dicarboxylate (**34**)

Compound **34** was prepared following the general procedure, starting from acetate **22** (53 mg, 0.21 mmol) and DEAD, without water addition. The reaction was complete in 50 min. Purification by flash chromatography (EtOAc/*n*-hexane, 1:2; *R*_*f*_ = 0.24) afforded **34** (55 mg, 68%) as a white foam. ^1^H NMR (400 MHz, CDCl_3_) (2.8 : 1 mixture of rotamers): δ 7.52–7.46 (m, 2H), 7.44–7.39 (m, 3H), 7.01 (br s, 1H, major), 6.87 (br s, 1H, minor), 6.42–6.38 (m, 1H), 4.30–4.16 (m, 2H), 4.09 (q, *J* = 7.2 Hz, 2H), 4.06 (br s, 1H, major), 3.90 (br s, 1H, minor), 2.08–1.96 (m, 2H), 1.94–1.85 (m, 1H), 1.68–1.61 (m, 1H), 1.47–1.37 (m, 3H), 1.30 (t, *J* = 7.2 Hz, 3H), 1.17–1.12 (m, 3H), 1.08–1.02 (m, 1H). ^13^C{^1^H} NMR (100.4 MHz, CDCl_3_) (mixture of rotamers): δ 206.9, 175.8, 157.0, 154.9, 134.0, 130.3, 128.7 (2 C), 127.4 (2 C), 126.9, 69.0, 62.6, 62.1, 46.5 and 46.0, 28.7 and 28.5, 22.3 and 22.1, 18.5, 18.2 and 18.1, 14.5, 14.4 and 14.2. MS (ESI) *m/z* (%): 795 ([2M + Na]^+^, 100), 409 ([M + Na]^+^, 30). IR (CHCl_3_): 3400, 3027, 3015, 2946, 2873, 1748, 1707, 1337 cm^–1^. Anal. Calcd for C_21_H_26_N_2_O_5_: C, 65.27; H, 6.78; N, 7.25. Found: C, 65.29; H, 7.13; N, 7.20.

#### Diisopropyl (3aS*,7aR*)-1-(1-Phenyl-3-oxo-3,4,5,6,7,7a-hexahydro-3aH-inden-3a-yl)-hydrazine 1,2-Dicarboxylate (**35**)

Compound **35** was prepared following the general procedure, starting from acetate **22** (68 mg, 0.27 mmol) and DIAD, without water addition. The reaction was complete in 30 min. Purification by flash chromatography (EtOAc/*n*-hexane, 1:3; *R*_*f*_ = 0.23) afforded **35** (79 mg, 71%) as a white foam. ^1^H NMR (400 MHz, CDCl_3_) (4.5 : 1 mixture of rotamers): δ 7.51–7.49 (m, 2H), 7.43–7.41 (m, 3H), 6.73 (br s, 1H, major), 6.52 (br s, 1H, minor), 6.42 (s, 1H, minor), 6.39 (s, 1H, major), 5.06–4.95 (m, 1H), 4.89–4.83 (m, 1H), 4.00 (m, 1H, major), 3.90 (m, 1H, minor), 2.08–1.96 (m, 2H), 1.94–1.85 (m, 1H), 1.66–1.60 (m, 1H), 1.48–1.38 (m, 3H), 1.30 (d, *J* = 6.4 Hz, 3H), 1.29 (d, *J* = 6.4 Hz, 3H), 1.19–1.14 (m, 6H), 1.08–1.02 (m, 1H). ^13^C{^1^H} NMR (100.4 MHz, CDCl_3_) (mixture of rotamers): δ 206.8, 175.6 and 175.0, 156.8 and 156.4, 154.5, 134.3, 130.3, 128.7 (2 C), 127.4 (2 C), 126.9, 70.6, 70.2 and 70.0, 69.0 and 68.8, 46.6 and 46.2, 28.8 and 28.5, 22.1, 22.0, 21.9 (2 C), 21.7, 18.5, 18.3. MS (ESI) *m/z* (%): 851 ([2M + Na]^+^, 100), 437 ([M + Na]^+^, 47). IR (CHCl_3_): 3400, 3026, 2985, 2942, 2874, 1746, 1707, 1376, 1244 cm^–1^. Anal. Calcd for C_23_H_30_N_2_O_5_: C, 66.65; H, 7.30; N, 6.76. Found: C, 66.38; H, 7.30; N, 6.61.

#### Diethyl (4aS*,7aR*)-1-(7-Methyl-5-oxo-2-tosyl-1,2,3,4,5,7a-hexahydro-[2]pyrindin-4a-yl)-hydrazine 1,2-Dicarboxylate (**36**)

Compound **36** was prepared following the general procedure, starting from acetate **23** (48 mg, 0.14 mmol) and DEAD. The reaction was complete in 4 h. Purification by flash chromatography (EtOAc/*n*-hexane, 1:1; *R*_*f*_ = 0.29) afforded **36** (51 mg, 76%) as a white solid. mp 210.4–211.7 °C. ^1^H NMR (400 MHz, CDCl_3_) (2.8 : 1 mixture of rotamers): δ 7.61 (d, *J* = 8.0 Hz, 2H), 7.30 (d, *J* = 8.0 Hz, 2H), 7.05 (br s, 1H, major), 6.74 (br s, 1H, minor), 6.02 (s, 1H), 4.11–3.87 (m, 5H), 3.72–3.62 (m, 1H, minor), 3.44–3.34 (m, 2H), 2.93 (dd, *J* = 12.4, 3.2 Hz, 1H, major), 2.71 (br d, *J* = 12.4 Hz, 1H, minor), 2.39 (s, 3H), 2.43–2.34 (m, 1H), 2.19 (s, 3H), 2.03–1.97 (m, 1H), 1.86–1.79 (m, 1H), 1.14–1.06 (m, 6H). ^13^C{^1^H} NMR (100.4 MHz, CDCl_3_) (mixture of rotamers): δ 204.0, 175.3, 156.8, 154.6, 143.5, 132.9, 129.6 (2 C), 127.8, 127.5 (2 C), 66.4 and 66.1, 62.8 and 62.6, 62.2, 48.5 and 48.1, 42.2, 42.0, 28.9, 21.4, 17.2, 14.2, 14.1. MS (ESI) *m/z* (%): 981 ([2M + Na]^+^, 100), 502 ([M + Na]^+^, 32), 480 ([M + 1]^+^, 8). IR (CHCl_3_): 3392, 3032, 2985, 2873, 1748, 1717, 1328, 1233 cm^–1^. Anal. Calcd for C_22_H_29_N_3_O_7_S: C, 55.10; H, 6.10; N, 8.76; S, 6.69. Found: C, 55.02; H, 6.13; N, 8.74; S, 6.68.

#### Diisopropyl (4aS*,7aR*)1-(7-Methyl-5-oxo-2-tosyl-1,2,3,4,5,7a-hexahydro-[2]pyrindin-4a-yl)-hydrazine 1,2-Dicarboxylate (**37**)

Compound **37** was prepared following the general procedure, starting from acetate **23** (190 mg, 0.55 mmol) and DIAD. The reaction was complete in 4.5 h. Purification by flash chromatography (EtOAc/*n*-hexane, 1:2; *R*_*f*_ = 0.05) afforded **37** (220 mg, 79%) as a white solid. mp 200.1–201.5 °C. ^1^H NMR (400 MHz, CDCl_3_) (6 : 1 mixture of rotamers): δ 7.61 (d, *J* = 8.4 Hz, 2H), 7.29 (d, *J* = 8.4 Hz, 2H), 6.71 (br s, 1H, major), 6.47 (br s, 1H, minor), 6.05 (s, 1H, minor), 6.02 (s, 1H, major), 4.83–4.77 (m, 1H), 4.64–4.58 (m, 1H), 4.02 (d, *J* = 12.8 Hz, 1H), 3.48–3.33 (m, 2H), 2.91 (dd, *J* = 12.8, 4.0 Hz, 1H, major), 2.81 (dd, *J* = 12.4, 4.4 Hz, 1H, minor), 2.58–2.50 (m, 1H, minor), 2.39 (s, 3H), 2.35–2.28 (m, 1H), 2.20 (s, 3H), 2.03–1.97 (m, 1H), 1.88–1.80 (m, 1H), 1.18 (d, *J* = 6.4 Hz, 3H), 1.14 (d, *J* = 6.4 Hz, 3H), 1.10 (d, *J* = 6.4 Hz, 3H), 0.98 (d, *J* = 6.0 Hz, 3H), 0.74 (d, *J* = 6.0 Hz, 3H, minor). ^13^C{^1^H} NMR (100.4 MHz, CDCl_3_) (mixture of rotamers): δ 203.8, 174.9, 156.3, 154.2, 143.9 and 143.5, 132.9, 129.9 and 129.6 (2 C), 127.7, 127.5 and 127.4 (2 C), 71.0, 70.5 and 70.3, 65.8, 48.4, 42.5 and 42.1, 42.3 and 41.9, 29.1 and 28.9, 21.9 and 21.8, 21.7, 21.6, 21.4 and 21.3 (2 C), 17.2. MS (ESI) *m/z* (%): 1037 ([2M + Na]^+^, 100), 530 ([M + Na]^+^, 87). IR (CHCl_3_): 3394, 3031, 2985, 1746, 1717, 1623, 1246 cm^–1^. Anal. Calcd for C_24_H_33_N_3_O_7_S: C, 56.79; H, 6.55; N, 8.28; S, 6.32. Found: C, 56.83; H, 6.59; N, 8.18; S, 6.30.

#### Diethyl (4aS*,7aR*)-1-(7-Methyl-5-oxo-3,4,5,7a-tetrahydro-1H-cyclopenta[c]pyran-4a-yl)-hydrazine 1,2-Dicarboxylate (**38**)

Compound **38** was prepared following the general procedure, starting from acetate **24** (81 mg, 0.42 mmol) and DEAD. The reaction was complete in 60 min. Purification by flash chromatography (EtOAc/*n*-hexane, 1:1; *R*_*f*_ = 0.39) afforded **38** (125 mg, 92%) as a white solid. mp 41.7–46.5 °C. ^1^H NMR (400 MHz, CDCl_3_) (5.8 : 1 mixture of rotamers): δ 6.81 (br s, 1H, major), 6.59 (br s, 1H, minor), 6.04 (s, 1H), 4.30–4.19 (m, 3H), 4.17–4.08 (m, 2H), 3.99 (dd, *J* = 12.8, 4.0 Hz, 1H, major), 3.86 (dd, *J* = 12.4, 4.0 Hz, 1H, minor), 3.77–3.72 (m, 1H), 3.36 (td, *J* = 11.2, 2.0 Hz, 1H), 3.21 (m, 1H, major), 3.17 (m, 1H, minor), 2.17 (s, 3H), 2.06–1.98 (m, 1H), 1.84–1.76 (m, 1H), 1.31 (t, *J* = 7.2 Hz, 3H), 1.20 (t, *J* = 7.2 Hz, 3H). ^13^C{^1^H} NMR (100.4 MHz, CDCl_3_) (mixture of rotamers): δ 205.1 and 204.8, 175.1 and 174.4, 157.0 and 156.5, 154.8, 127.7 and 127.4, 65.9 and 65.8, 63.5, 63.3 and 63.1, 62.7, 62.4 and 62.3, 48.2 and 47.9, 29.3 and 29.0, 17.2, 14.4 and 14.3, 14.2 and 14.1. MS (ESI) *m/z* (%): 675 ([2M + Na]^+^, 100), 349 ([M + Na]^+^, 25), 327 ([M + 1]^+^, 2). IR (CHCl_3_): 3393, 3028, 3014, 2985, 2878, 1749, 1718, 1379, 1239 cm^–1^. Anal. Calcd for C_15_H_22_N_2_O_6_: C, 55.21; H, 6.79; N, 8.58. Found: C, 55.20; H, 6.80; N, 8.56.

#### Diethyl (3aS*,8aR*)-1-(1-Methyl-3-oxo-4,5,6,7,8,8a-hexahydro-3H-azulen-3a-yl)-hydrazine 1,2-Dicarboxylate (**39**)

Compound **39** was prepared following the general procedure, starting from acetate **25** (46 mg, 0.22 mmol) and DEAD, without water addition. The reaction was complete in 2 h. Purification by flash chromatography (EtOAc/*n*-hexane, 1:3; R_*f*_ = 0.13) afforded **39** (64 mg, 85%) as a white solid. mp 130.8–132.3 °C. ^1^H NMR (400 MHz, CDCl_3_) (9 : 1 mixture of diastereoisomers; major diastereoisomer as a 4.3 : 1 mixture of rotamers): δ 6.62 (br s, 1H major rotamer), 6.55 (br s, 1H, minor diastereoisomer), 6.39 (br s, 1H minor rotamer), 6.12 (s, 1H, both rotamers), 6.02 (s, 1H, minor diastereoisomer), 4.28–4.13 (m, 2H), 4.11–3.99 (m, 2H), 3.69 (m, 1H, minor diastereoisomer), 3.48 (m, 1H, major rotamer), 3.33 (m, 1H, minor rotamer), 2.17–2.08 (m, 1H), 2.10 (s, 3H), 2.02–1.96 (m, 1H), 1.91–1.82 (m, 1H), 1.71–1.58 (m, 4H), 1.30 (t, *J* = 6.8 Hz, 3H), 1.22–1.14 (m, 1H), 1.16 (t, *J* = 6.8 Hz, 3H), 1.12–1.04 (m, 1H), 1.03–0.93 (m, 1H), 0.90–0.80 (m, 2H, minor rotamer). ^13^C{^1^H} NMR (100.4 MHz, CDCl_3_) (mixture of diastereoisomers and rotamers; major rotamer of the major diastereoisomer reported): δ 207.5, 179.6, 156.8, 154.7, 130.4, 72.6, 62.4, 62.1, 54.2, 35.0, 31.5, 31.0, 25.4, 22.6, 17.6, 14.4, 14.0. MS (ESI) *m/z* (%): 699 ([2M+ Na]^+^, 100), 361 ([M + Na]^+^, 51). IR (CHCl_3_): 3406, 3027, 2932, 2859, 1747, 1714, 1379, 1236 cm^–1^. Anal. Calcd for C_17_H_26_N_2_O_5_: C, 60.34; H, 7.74; N, 8.28. Found: C, 60.42; H, 7.77; N, 7.96.

#### Diethyl (3aS*,8aR*)-1-(1-Isopropyl-3-oxo-4,5,6,7,8,8a-hexahydro-3H-azulen-3a-yl)-hydrazine 1,2-Dicarboxylate (**40**)

Compound **40** was prepared following the general procedure, starting from acetate **26** (61 mg, 0.26 mmol) and DEAD. The reaction was complete in 40 min. Purification by flash chromatography (EtOAc/*n*-hexane, 1:3; R_*f*_ = 0.22) afforded **40** (74 mg, 78%) as a white solid. mp 129.9–132.2 °C. ^1^H NMR (400 MHz, CDCl_3_) (4.5 : 1 mixture of diastereoisomers; major diastereoisomer as a 3 : 1 mixture of rotamers): δ 6.63 (br s, 1H major rotamer), 6.54 (br s, 1H, minor diastereoisomer), 6.38 (br s, 1H minor rotamer), 6.11 (s, 1H, both rotamers), 6.03 (s, 1H, minor diastereoisomer), 4.32–4.13 (m, 2H), 4.12–3.98 (m, 2H), 3.91 (m, 1H, minor diastereoisomer), 3.64–3.62 (m, 1H, major rotamer), 3.50 (m, 1H, minor rotamer), 2.62–2.51 (m, 1H), 2.19–2.08 (m, 1H), 2.01–1.83 (m, 2H), 1.71–1.58 (m, 4H), 1.30 (t, *J* = 6.8 Hz, 3H), 1.24 (d, *J* = 6.4 Hz, 3H), 1.22–1.18 (m, 1H), 1.17–1.14 (m, 6H), 1.07–0.94 (m, 2H), 0.91–0.79 (m, 2H, minor rotamer). ^13^C{^1^H} NMR (100.4 MHz, CDCl_3_) (mixture of diastereoisomers and rotamers; major rotamer of the major diastereoisomer reported): δ 208.0, 189.3, 157.0, 154.7, 126.6, 72.8, 62.4, 62.1, 51.6, 35.1, 31.1, 29.2, 25.7, 25.6, 22.3, 21.2, 19.9, 14.5, 14.3. MS (ESI) *m/z* (%): 755 ([2M + Na]^+^, 100), 389 ([M + Na]^+^, 25). IR (CHCl_3_): 3420, 3026, 2971, 2932, 2859, 1747, 1710, 1378, 1236 cm^–1^. Anal. Calcd for C_19_H_30_N_2_O_5_: C, 62.27; H, 8.25; N, 7.64. Found: C, 62.30; H, 8.27; N, 7.59.

#### Diisopropyl (3aS*,8aR*)-1-(1-Isopropyl-3-oxo-4,5,6,7,8,8a-hexahydro-3H-azulen-3a-yl)-hydrazine 1,2-Dicarboxylate (**41**)

Compound **41** was prepared following the general procedure, starting from acetate **26** (69 mg, 0.30 mmol) and DIAD. The reaction was complete in 30 min. Purification by flash chromatography (EtOAc/*n*-hexane, 1:4; *R*_*f*_ = 0.15) afforded **41** (92 mg, 79%) as a white solid. mp 154.0–158.4 °C. ^1^H NMR (400 MHz, CDCl_3_) (6.5 : 1 mixture of diastereoisomers; major diastereoisomer as a 4 : 1 mixture of rotamers): δ 6.50 (br s, 1H major rotamer), 6.43 (br s, 1H, minor diastereoisomer), 6.29 (br s, 1H minor rotamer), 6.11 (s, 1H, both rotamers), 6.02 (s, 1H, minor diastereoisomer), 4.99–4.90 (m, 1H), 4.85–4.76 (m, 1H), 3.94 (m, 1H, minor diastereoisomer), 3.63 (d, *J* = 5.6 Hz, 1H, major rotamer), 3.50 (d, *J* = 5.6 Hz, 1H, minor rotamer), 2.60–2.51 (m, 1H), 2.15–2.07 (m, 1H), 1.99–1.88 (m, 2H), 1.88–1.81 (m, 1H, major), 1.76–1.58 (m, 4H), 1.33–1.23 (m, 9H), 1.20–1.13 (m, 9H), 1.07–0.93 (m, 2H), 0.91–0.79 (m, 1H, major). ^13^C{^1^H} NMR (100.4 MHz, CDCl_3_) (mixture of diastereoisomers and rotamers; major rotamer of the major diastereoisomer reported): δ 208.0, 188.9, 156.7, 154.2, 126.7, 72.7, 70.4, 69.9, 51.6, 35.2, 31.2, 29.2, 25.73, 25.71, 22.2, 22.1, 22.0, 21.8, 21.7, 21.2, 19.9. MS (ESI) *m/z* (%): 811 ([2M+ Na]^+^, 100), 417 ([M + Na]^+^, 26). IR (CHCl_3_): 3404, 3027, 2984, 2933, 2859, 1740, 1706, 1376, 1239 cm^–1^. Anal. Calcd for C_21_H_34_N_2_O_5_: C, 63.93; H, 8.69; N, 7.10. Found: C, 63.95; H, 8.70; N, 7.11.

#### Diethyl (3aS*,8aR*)-1-(1-Butyl-3-oxo-4,5,6,7,8,8a-hexahydro-3H-azulen-3a-yl)-hydrazine 1,2-Dicarboxylate (**42**)

Compound **42** was prepared following the general procedure, starting from acetate **27** (69 mg, 0.28 mmol) and DEAD. The reaction was complete in 30 min. Purification by flash chromatography (EtOAc/*n*-hexane, 1:3; *R*_*f*_ = 0.24) afforded **42** (94 mg, 88%) as a white solid. mp 84.2–87.3 °C. ^1^H NMR (400 MHz, CDCl_3_) (4.7 : 1 mixture of diastereoisomers; major diastereoisomer as a 3.2 : 1 mixture of rotamers): δ 6.81 (br s, 1H major rotamer), 6.63 (br s, 1H, minor diastereoisomer), 6.56 (br s, 1H minor rotamer), 6.11 (s, 1H, both rotamers), 6.01 (s, 1H, minor diastereoisomer), 4.21–3.96 (m, 4H), 3.75 (m, 1H, minor diastereoisomer), 3.50 (m, 1H, major rotamer), 3.35 (m, 1H, minor rotamer), 2.59–2.53 (m, 1H, minor diastereoisomer), 2.44–2.36 (m, 1H), 2.29–2.21 (m, 1H), 2.16–2.05 (m, 1H), 2.02–1.77 (m, 3H), 1.72–1.48 (m, 5H), 1.42–1.34 (m, 2H), 1.27 (t, *J* = 7.2 Hz, 3H), 1.23–1.16 (m, 1H), 1.14 (t, *J* = 6.8 Hz, 3H), 1.08–0.92 (m, 2H), 0.91 (t, *J* = 7.2 Hz, 3H). ^13^C{^1^H} NMR (100.4 MHz, CDCl_3_) (mixture of diastereoisomers and rotamers; major rotamer of the major diastereoisomer reported): δ 207.7, 183.9, 156.9, 154.7, 128.6, 72.5, 62.4, 62.1, 53.0, 35.0, 31.1, 31.0, 28.9, 25.7, 25.5, 22.5, 22.2, 14.5, 14.2, 13.8. MS (ESI) *m/z* (%): 783 ([2M+ Na]^+^, 100), 403 ([M + Na]^+^, 61), 381 ([M + 1]^+^, 5). IR (CHCl_3_): 3406, 3014, 2933, 2861, 1747, 1711, 1334, 1236 cm^–1^. Anal. Calcd for C_20_H_32_N_2_O_5_: C, 63.13; H, 8.48; N, 7.36. Found: C, 63.15; H, 8.48; N, 7.41.

#### Diethyl (1S*,3aS*,7aR*)-1-(1-Methyl-3-oxo-octahydroinden-3a-yl)-hydrazine 1,2-Dicarboxylate (**43**)

To a solution of **12** (160 mg, 0.49 mmol) in MeOH (9.0 mL), 10% Pd/C wet (107 mg, 0.045 mmol) was added under a nitrogen atmosphere. The resulting suspension was first flushed with hydrogen under vigorous stirring and then maintained under a hydrogen atmosphere (balloon) at room temperature. After 2 h, the mixture was filtered over a Celite pad, and the residual solution was evaporated under reduced pressure. The foamy residue was purified by flash chromatography (eluent: EtOAc/*n*-hexane, 1:4; *R*_*f*_ = 0.18), and pure compound **43** (158 mg, 99%) was obtained as a white foam. ^1^H NMR (400 MHz, CDCl_3_) (4.1 : 1 mixture of rotamers): δ 6.68–6.45 (m, 1H, major), 6.48–6.44 (m, 1H, minor), 4.29–4.16 (m, 2H), 4.14–4.07 (m, 2H), 2.57–2.48 (m, 2H), 2.25–2.11 (m, 2H), 1.99–1.89 (m, 1H), 1.84–1.65 (m, 2H and 1H major), 1.62–1.48 (m, 2H), 1.40–1.28 (m, 3H), 1.29 (t, *J* = 7.2 Hz, 3H), 1.22 (t, *J* = 7.2 Hz, 3H), 1.12 (d, *J* = 6.0 Hz, 3H). ^13^C{^1^H} NMR (100.4 MHz, CDCl_3_) (mixture of rotamers): δ 215.8 and 215.4, 156.7 and 156.6, 155.3 and 155.2, 69.9 and 69.8, 62.5 and 62.4, 62.2 and 61.9, 44.9 and 44.7, 42.9 and 42.8, 27.9 and 27.8, 26.3 and 26.1, 21.4 and 21.3, 20.3 and 20.2, 19.4 and 19.3, 18.7, 14.5 and 14.4, 14.3 and 14.2. MS (ESI) *m/z* (%): 675 ([2M+ Na]^+^, 100), 349 ([M + Na]^+^, 27), 327 ([M + 1]^+^, 2). IR (CHCl_3_): 3385, 2959, 2937, 1748, 1705, 1379, 1339, 1317, 1234 cm^–1^. Anal. Calcd for C_16_H_26_N_2_O_5_: C, 58.88; H, 8.03; N, 8.58. Found: C, 58.91; H, 8.10; N, 8.60.

#### Diisopropyl (1S*,3aS*,7aR*)-1-(1-Methyl-3-oxo-octahydroinden-3a-yl)-hydrazine 1,2-Dicarboxylate (**44**)

It was prepared in the same way as reported for **43**, starting from **28** (82 mg, 0.23 mmol) and obtaining, after flash chromatography purification (eluent: EtOAc/*n*-hexane, 1:4; *R*_*f*_ = 0.21), compound **44** (81 mg, 99%) as a white foam. ^1^H NMR (400 MHz, CDCl_3_) (3.5 : 1 mixture of rotamers): δ 6.54 (br s, 1H, major), 6.35 (br s, 1H, minor), 5.06–4.94 (m, 1H), 4.88–4.82 (m, 1H), 2.58–2.48 (m, 2H), 2.26–2.12 (m, 2H), 1.96–1.92 (m, 1H, major), 1.87–1.77 (m, 1H, minor), 1.76–1.65 (m, 2H), 1.61–1.47 (m, 2H), 1.34–1.26 (m, 9H), 1.23–1.19 (m, 6H), 1.12 (d, *J* = 6.4 Hz, 3H). ^13^C{^1^H} NMR (100.4 MHz, CDCl_3_) (mixture of rotamers): δ 215.53 and 215.50, 156.5 and 156.4, 155.0 and 154.8, 70.5 and 70.4, 70.1 and 70.0, 69.7, 45.0 and 44.7, 42.9 and 42.8, 27.9 and 27.8, 26.5 and 26.1, 22.0, 21.9, 21.86, 21.8, 21.4 and 21.1, 20.3 and 20.2, 19.4, 18.8. MS (ESI) *m/z* (%): 731 ([2M+ Na]^+^, 51), 377 ([M + Na]^+^, 100), 355 ([M + 1]^+^, 13). IR (CHCl_3_): 3393, 2985, 2938, 1748, 1700, 1387, 1375, 1314, 1239 cm^–1^. Anal. Calcd for C_18_H_30_N_2_O_5_: C, 61.00; H, 8.53; N, 7.90. Found: C, 60.98; H, 8.57; N, 7.85.

## Data Availability

The data underlying this study are available in the published article and its online Supporting Information.

## Supporting Information Available

Structure assignment by NMR studies; proton NMR experiment carried out in CD_2_Cl_2_; copies of ^1^H and ^13^C{^1^H} NMR spectra for all new compounds; ^1^H NMR spectra (enlarged view) of compound **12** recorded at variable temperatures (PDF)
