# Supplementary material for: Synthesis of 5-Hydrazino-2-cyclopentenone Derivatives by a Gold(I)-Catalyzed Cycloisomerization/Hetero-Diels–Alder/Ring-Opening Tandem Reaction of Enynyl Acetates
Source: J Org Chem. 2023 May 12;88(11):7015–25. doi: 10.1021/acs.joc.3c00310 (PMC10262200; doi:10.1021/acs.joc.3c00310)
Supplement: Supplementary file 1 — jo3c00310_si_001.pdf [file jo3c00310_si_001.pdf]

Synthesis of 5-Hydrazino-2-cyclopentenone Derivatives by a Gold(I)-catalyzed Cycloisomerization/Hetero Diels-Alder/Ring Opening Tandem Reaction of Enynyl Acetates

Dina Scarpi,[a] Nunzia Favale,[a] and Ernesto G. Occhiato[a,]*

[a]*Dipartimento di Chimica "U. Schiff", Università degli Studi di Firenze, Via della Lastruccia 13, 50019, Sesto Fiorentino (FI), Italy. E-mail:* ernesto.occhiato@unifi.it

Supporting Information

Table of contents

1. Structure assignment by NMR analysis S2-S3

2. Proton NMR Experiment carried out in CD_2Cl_2 S4-S6

3. Copies of ^{1}H and ^{13}C NMR spectra S7-S62

4. ^{1}H NMR spectra (enlarged view) of compound 12 recorded at variable T S63

Structure assignment by NMR analysis

Compounds 12 and 13

Compound **12** was fully characterized by ESI-MS, elemental analysis determination, IR, and mono- and bidimensional NMR (in $CDCl_3$) spectroscopies. ESI-MS and elemental analysis were consistent with the molecular formula (see the Experimental Section), so the following discussion refers to the NMR spectroscopic analysis only. Compound **13** was characterized by ESI-MS, IR, and mono- and bidimensional NMR (in $CDCl_3$) spectroscopies. See Figure S1 for structures and numbering used. Both compounds are mixtures of two rotamers (in a 3:1 ratio).

*Compound **12** (major rotamer).* In compound **12**, the NH signal of the hydrazine appendage resonates at 6.70 ppm, a signal which disappears if D_2O is added, the olefinic 2-H at 5.98, the methyl group at 2.09 ppm, and finally the bridgehead 3a-H proton at 3.40 ppm as a broad singlet. This compound shows analogies with compound **S1**[1] described by Zhang as it possesses a six-membered ring-fused cyclopentenone and an electronegative atom at position 7a. Diagnostic are the ^{13}C NMR resonances: in compound **S1**, the conjugated enone system is characterized by the signals at 210.8 ppm (C=O), 183.2 ppm (C3), and 125.2 ppm (C2), that almost exactly match those of compound **12**, i.e., 206.6 ppm (C=O), 176.8 ppm (C3), and 127.3 (C2). The C7a atom bearing the hydrazine moiety in **12** resonates at 68.6 ppm, just slightly more upfield shifted than the same C atom in **S1** (as expected, being the O atom more electronegative than N) which resonates at 77.9 ppm. Finally, the bridgehead C3a atom resonates almost at the same chemical shift in compounds **12** (48.1 ppm) and **S1** (49.0 ppm). Finally, in compound **12**, two more signals at 156.8 and 154.9 ppm are found correspondent to the two carbamate quaternary C atoms.

*Compound **13** (major rotamer).* It has very similar spectra than compound **12**, but it presents the ^{1}H and ^{13}C NMR signals of the *N*-acetyl moiety. The CH_3 of this group resonate as a singlet at 2.50 ppm in the ^{1}H NMR spectrum, and at 49.7 ppm in the ^{13}C NMR spectrum. The carbonyl C atom of the *N*-acetyl moiety is found at 171.6 ppm.

Other compounds

*Compound **14***. This compound was not isolated and ^{1}H NMR data refers to those collected during the experiment in CD_2Cl_2 described in the text. Apart from the *t*-butyloxy moiety signals, the ^{1}H NMR spectrum almost exactly matches that of known compound **20** which we have instead isolated in this work. The singlet at 5.90 ppm can be assigned to the olefinic proton (6.07 ppm in **20**, $CDCl_3$) and the singlet at 1.93 ppm at the methyl group at C3 (1.95 ppm in **20**, $CDCl_3$). A diagnostic signal of all compounds possessing such structure is an upfield shifted quartet of doublets at 0.78 ppm, attributable to one of the 4-H protons (0.87 ppm in **20**, $CDCl_3$). Finally, the bridgehead 3a-H proton resonates as a dd at 2.48 ppm in **14** and at 2.54 ppm ($CDCl_3$) in **20**.

*Compounds **15** and **19***. Compound **15** was not isolated and ^{1}H NMR data refers to those collected during the experiment in CD_2Cl_2 described in the text. Apart from the *t*-butyloxy moiety signals, the ^{1}H NMR spectrum

almost exactly matches that of compound **19** which we have instead isolated (as a single diastereomer) in this work and partially characterized. Also, the ^{1}H NMR spectrum is very similar (apart from the obvious differences due to the different appendage at C1) to that of compound **S2** we have recently reported.[2] The most notable signal which demonstrates that the [4+2] cycloaddition did occur is that of the methyl group at C3 which is upfield shifted in **15** compared to the corresponding one in substrate **14**, resonating at 1.71 ppm in **15** and at 1.78 ppm in **19** ($CDCl_3$). We had observed such upfield shift also in compound **S2**, in which the methyl group resonated at 1.70 ppm in $CDCl_3$, and in its *N*-Boc analogue (1.66 ppm, $CDCl_3$), whose structure had been confirmed by X-ray analysis.[2] As expected, the 2-H proton is slightly upfield shifted, too, at 5.71 ppm in **15** and 6.00 ppm in **19** ($CDCl_3$) for the ED effect by the acyloxy group at C1. The bridgehead 3a-H proton resonates at 2.77 and 2.93 ($CDCl_3$) ppm in **15** and **19** respectively, as a broad doublet, consistently with the chemical shift value of the same proton in **S2** (2.59 ppm, $CDCl_3$; 2.55 ppm in its *N*-Boc analogue).

*Compound **17***. Compound **17** was not isolated and ^{1}H NMR data refers to those collected during the experiment in CD_2Cl_2 described in the text. The only signal attributable to compound **17** and which is diagnostic of the occurred ring opening is the singlet at 1.83 ppm of the methyl group at C3 which is downfield shifted from 1.71 ppm (**15**) because of its allylic position (in analogy to **S3**[2]). Expectedly, 2-H proton does not change significantly its chemical shift going from **15** (5.71 ppm) to **17** (5.74 ppm), being in both compounds at the b position to the acyloxy group.

Figure S1

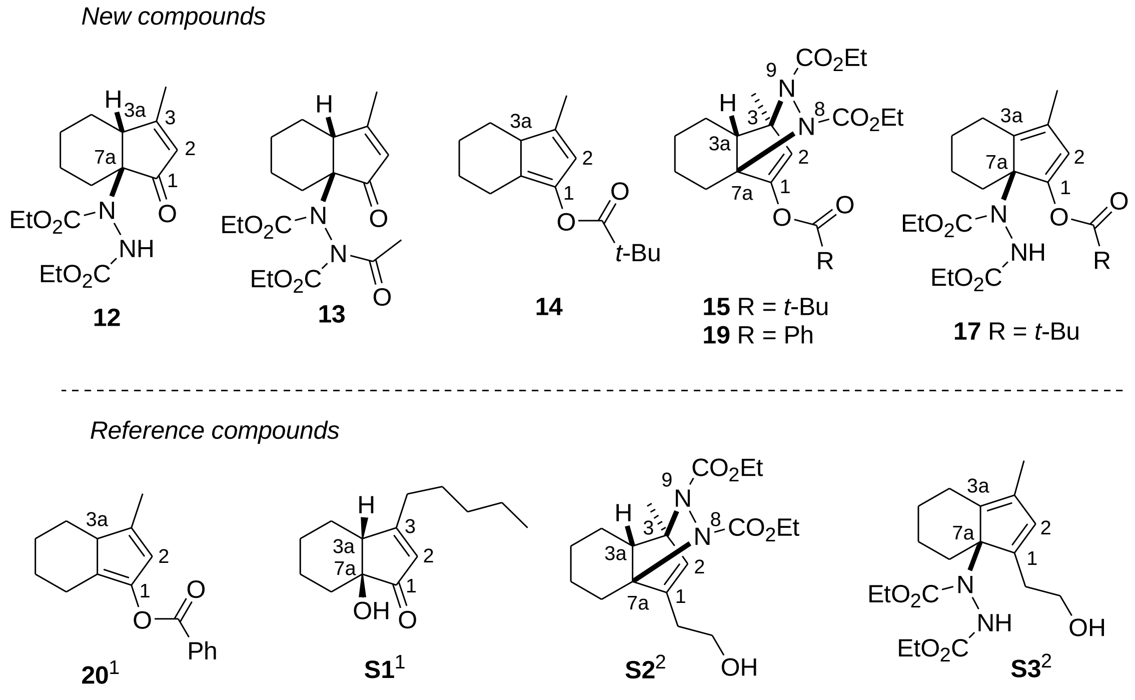

References

[1] Zhao, K.; Hsu, Y.-C.; Yang, Z.; Liu, R.-L.; Zhang, L. *Org. Lett.* **2020**, *22*, 6500.

[2] Scarpi, D.; Bagni, F.; Faggi, C.; Carral-Menoyo, A.; Gómez-Bengoa, E.; Occhiato, E. G. *J. Org. Chem.* **2022**, *87*, 6038.

Proton NMR Experiment carried out in CD_2Cl_2

We report here the results of one experiment carried out with pivaloyl ester **10** in NMR tube with CD_2Cl_2 as the solvent and monitored directly by ^{1}H NMR (Scheme S1). Most significant spectra recorded during the gold(I)-catalyzed cycloisomerization/hetero Diels-Alder/ring opening tandem reaction of propargyl pivaloate **10** are reported in Figures S2-S5. The main diagnostic signals, belonging to the intermediates, are highlighted in different colors.

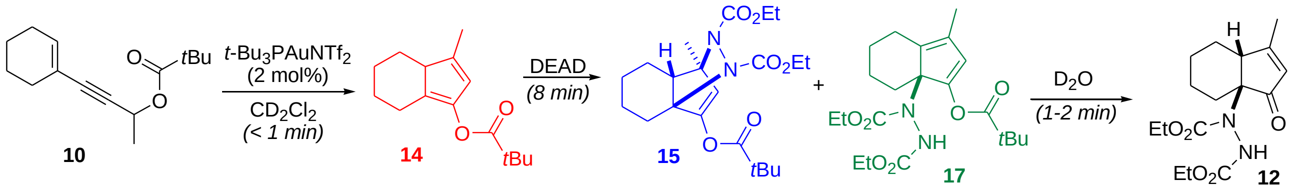

Scheme S1.

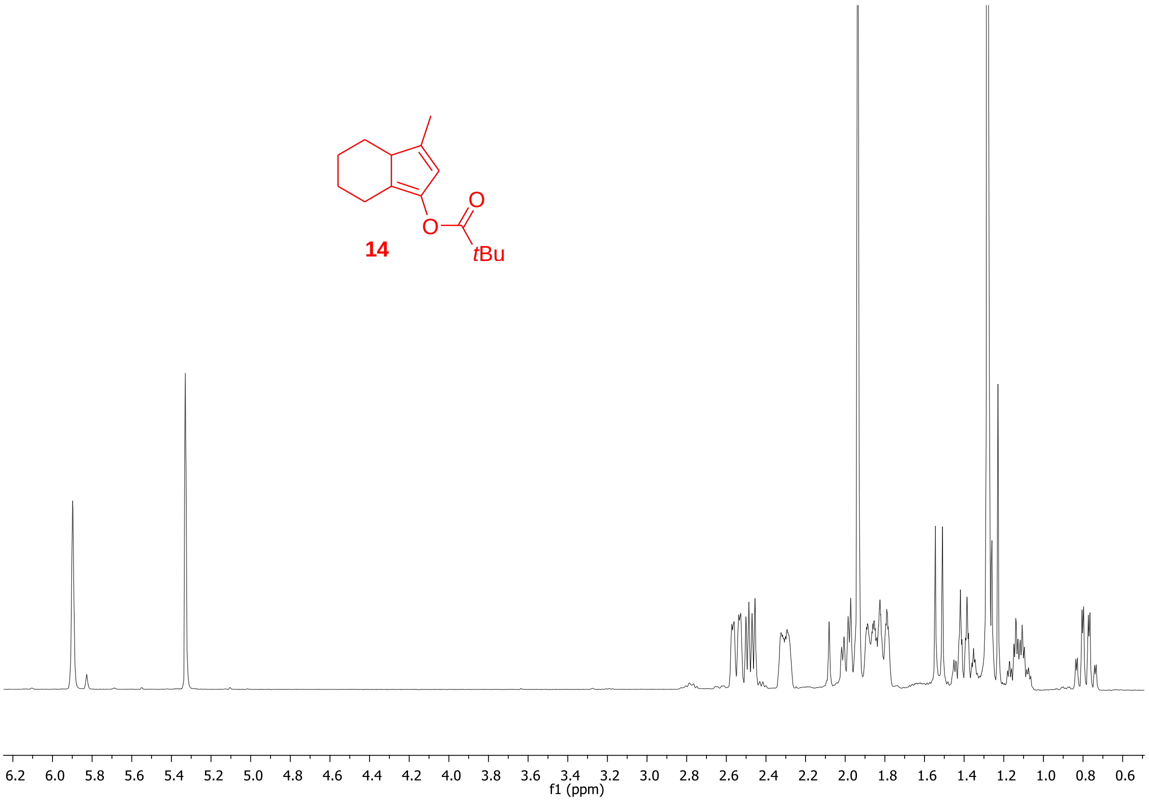

Figure S2. ^{1}H NMR (CD_2Cl_2, 400 MHz) spectrum at 1 minute after the addition of substrate **10** to the solution of $t$$Bu_3PAuNTf_2$.

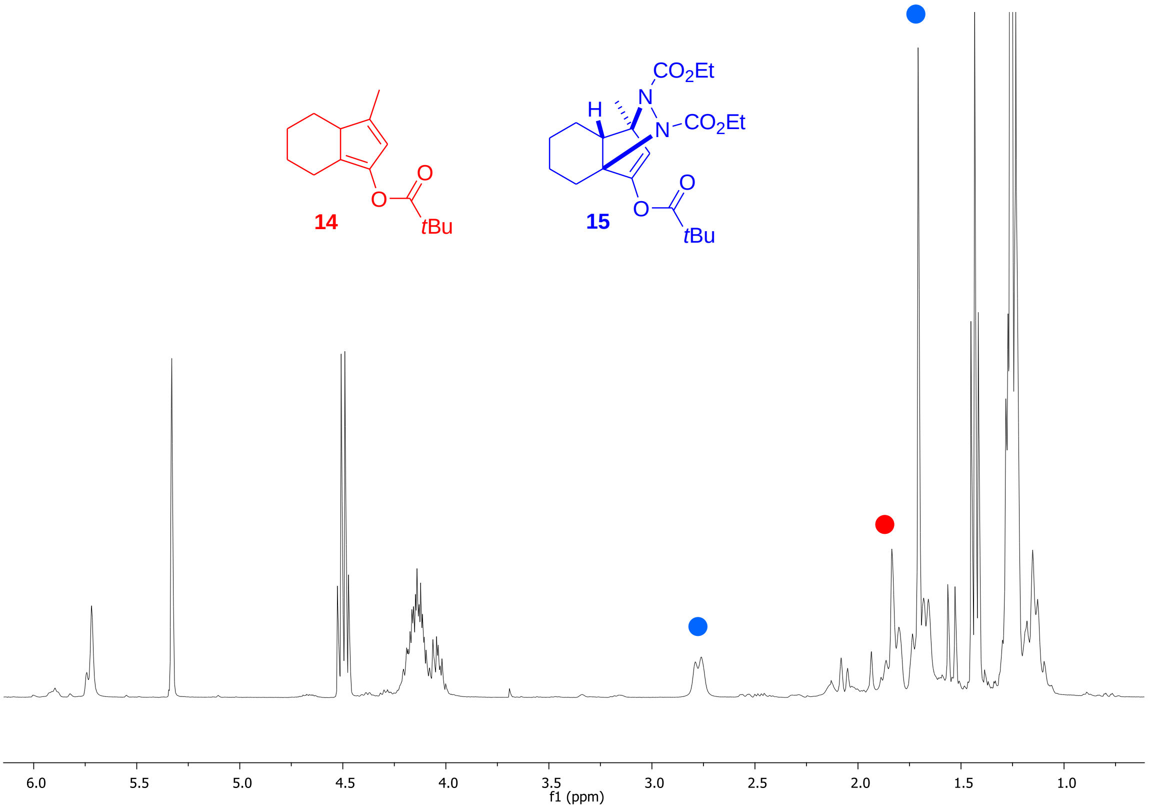

Figure S3. ^{1}H NMR (CD_2Cl_2, 400 MHz) spectrum at 8 minutes after the addition of DEAD to the solution of intermediate **14**. Main diagnostic signals of compounds **14** (red) and **15** (blue) are pointed out.

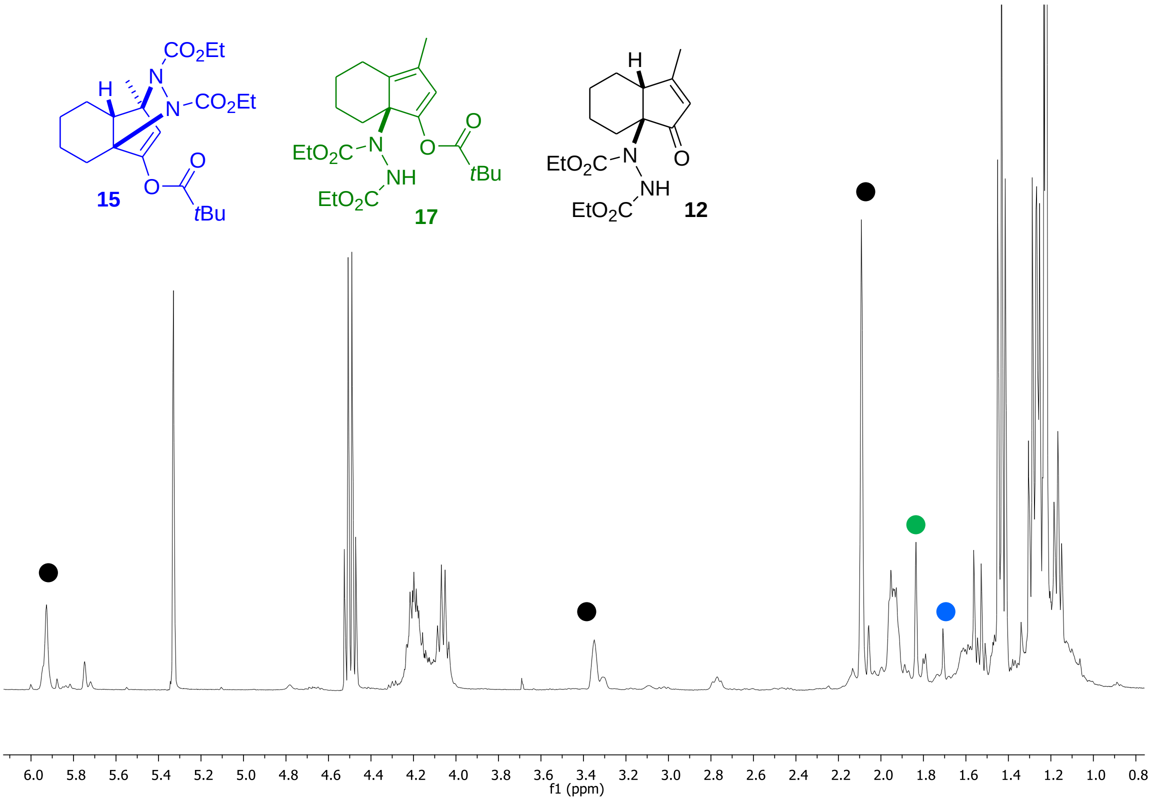

Figure S4. ^{1}H NMR (CD_2Cl_2, 400 MHz) spectrum at 2 minutes after the addition of D_2O to the solution of intermediate **15**. Main diagnostic signals of compounds **15** (blue), **17** (green) and **12** (black) are pointed out.

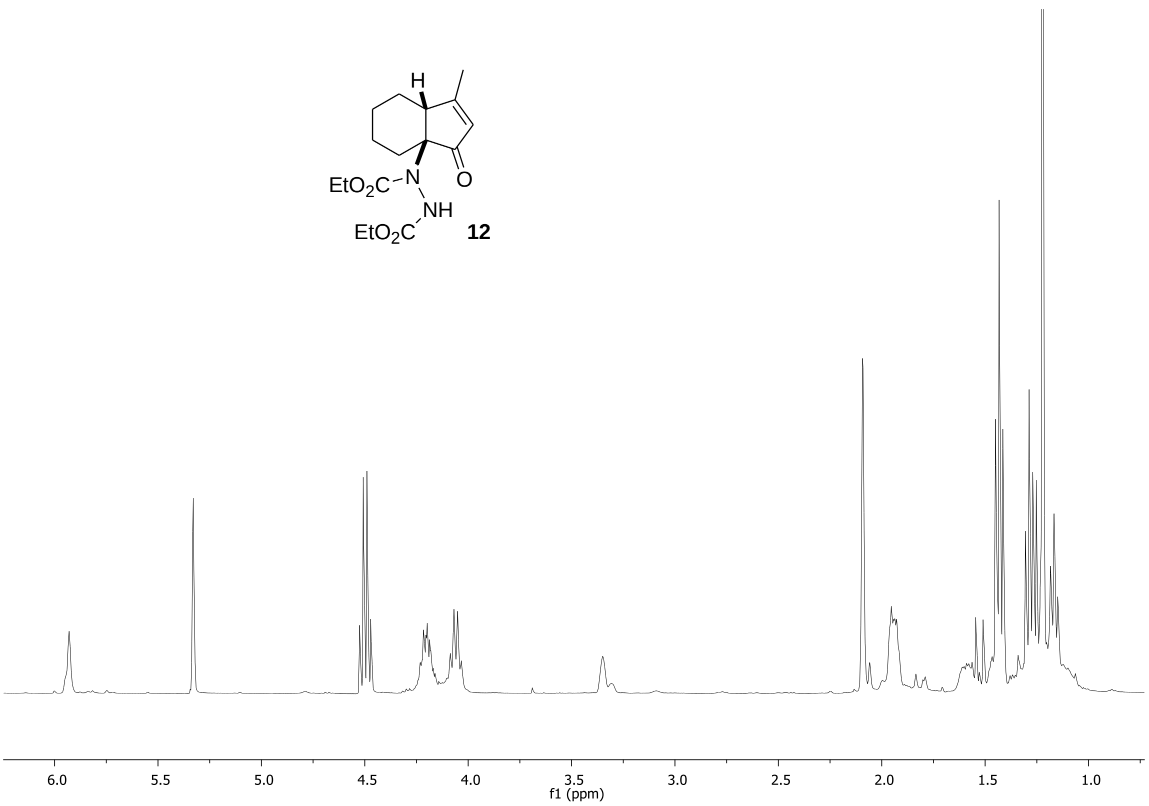

Figure S5. ^{1}H NMR (CD_2Cl_2, 400 MHz) spectrum at 10 minutes after the addition of D_2O to the solution of intermediate **15**: only compound **12** is detectable.

.

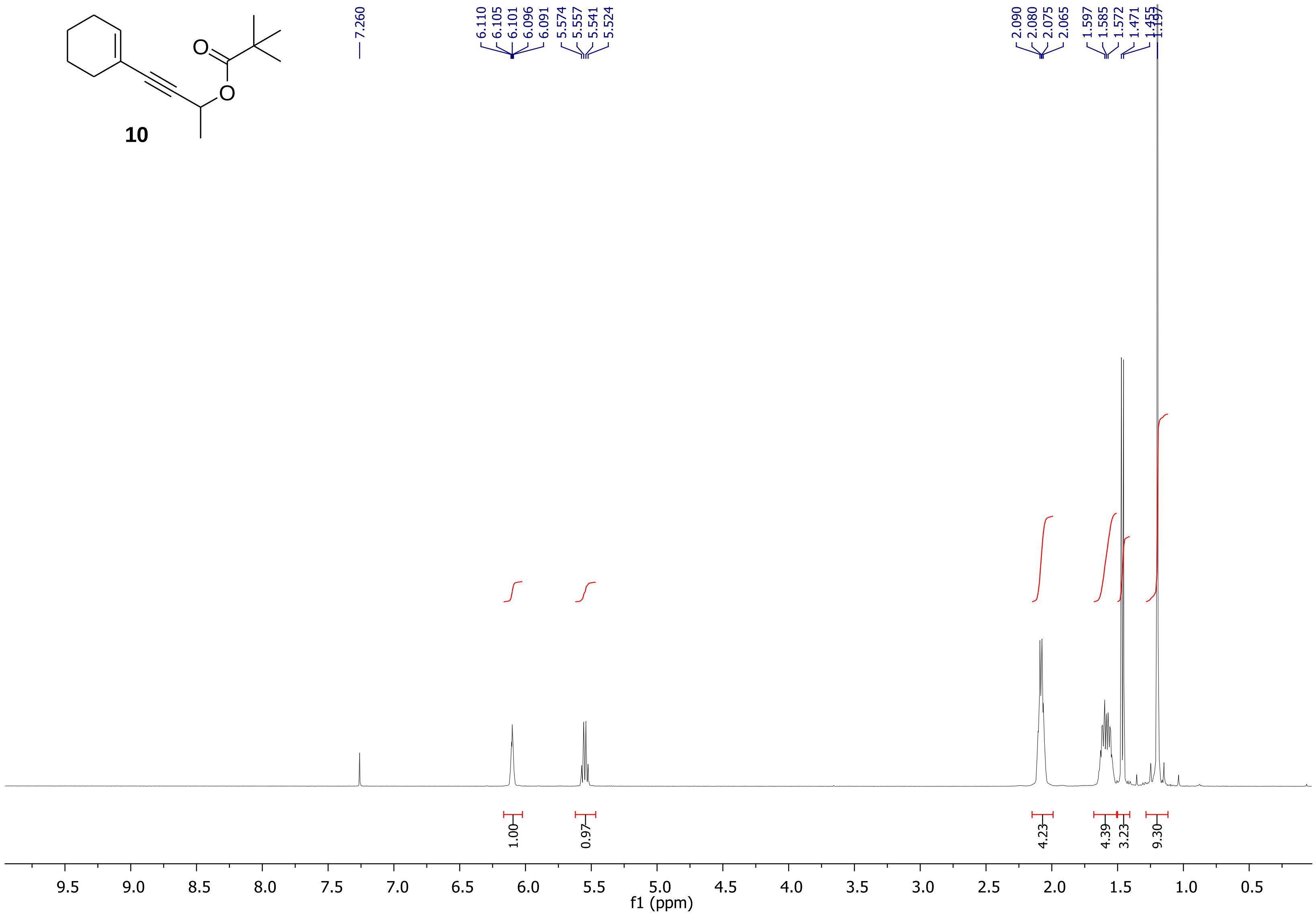

^{1}H NMR ($CDCl_3$, 400 MHz) of compound **10**

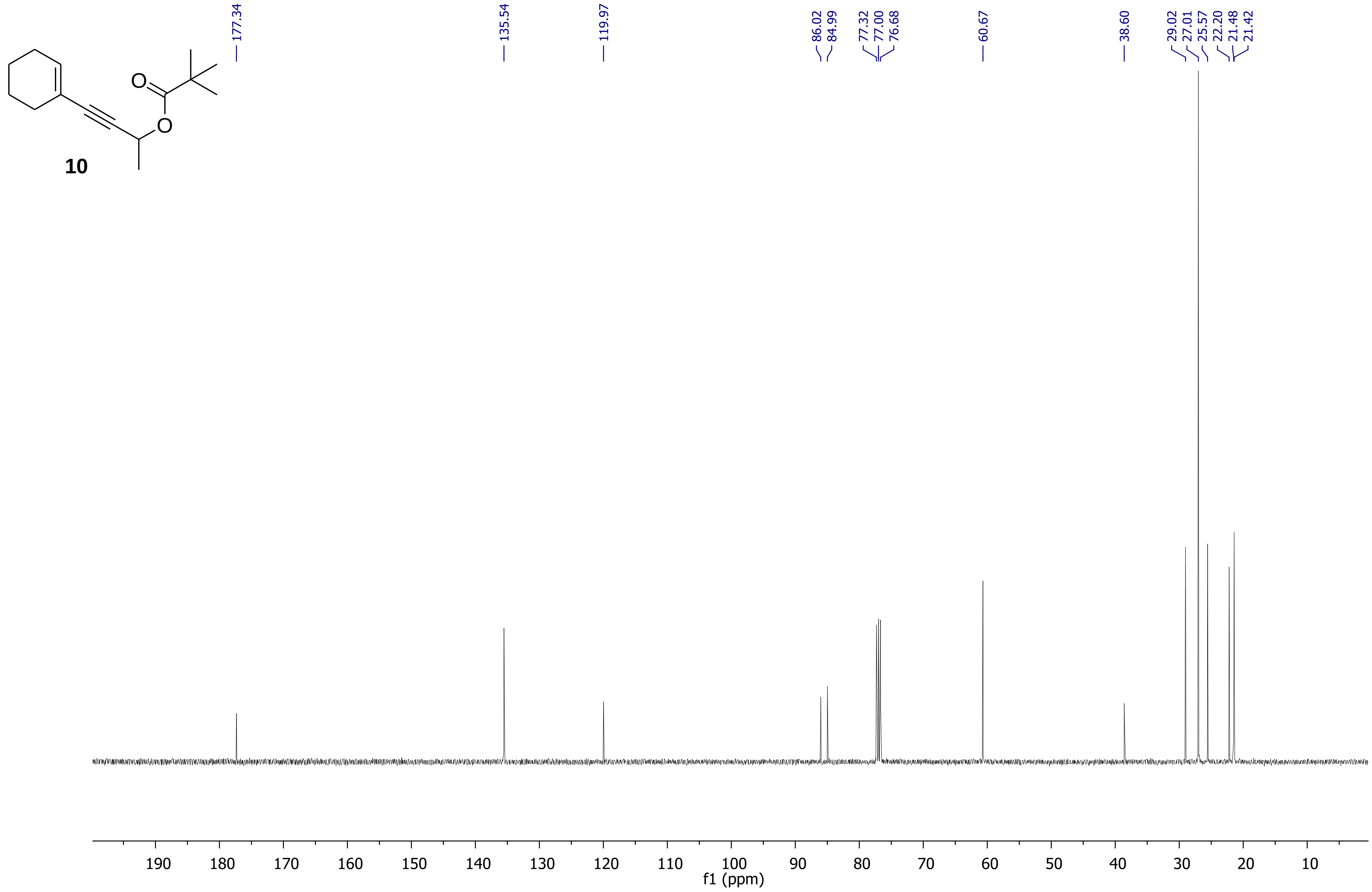

$^{13}C\{^{1}H\}$ NMR ($CDCl_3$, 100.4 MHz) of compound **10**

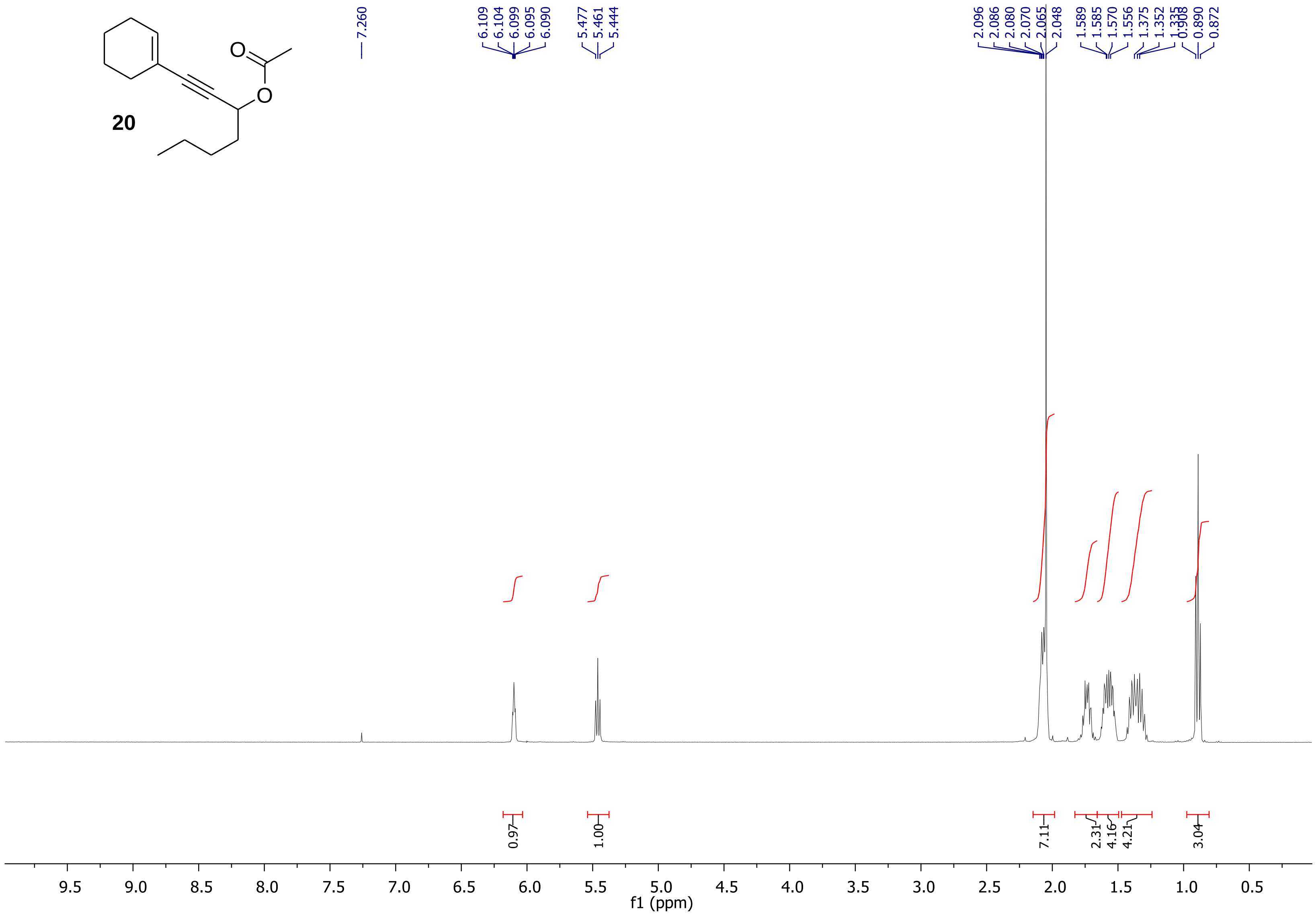

^{1}H NMR ($CDCl_3$, 400 MHz) of compound **20**

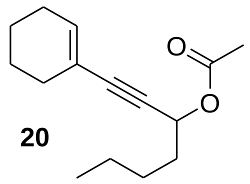

20

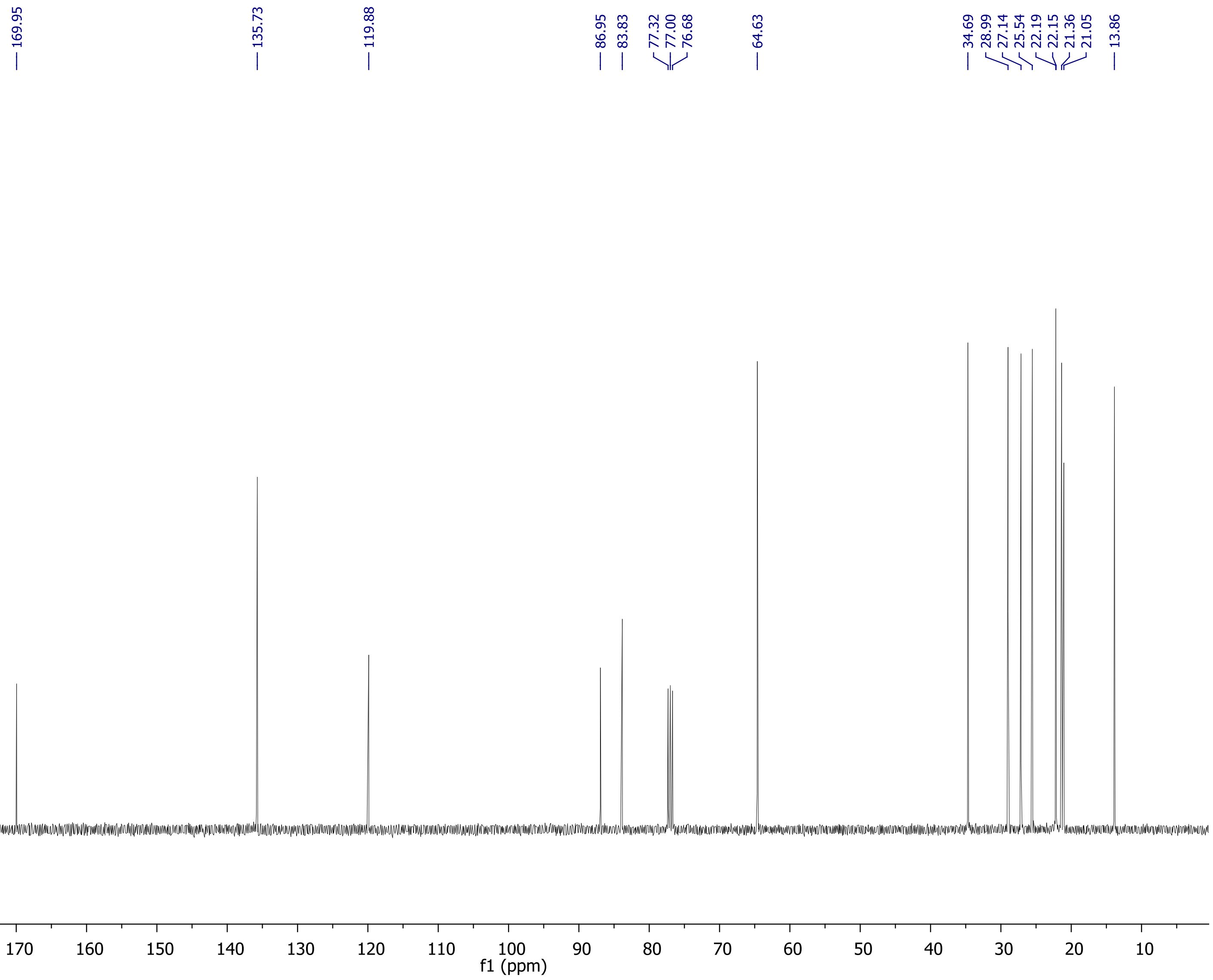

$^{13}C\{^1H\}$ NMR ($CDCl_3$, 100.4 MHz) of compound **20**

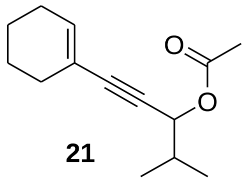

21

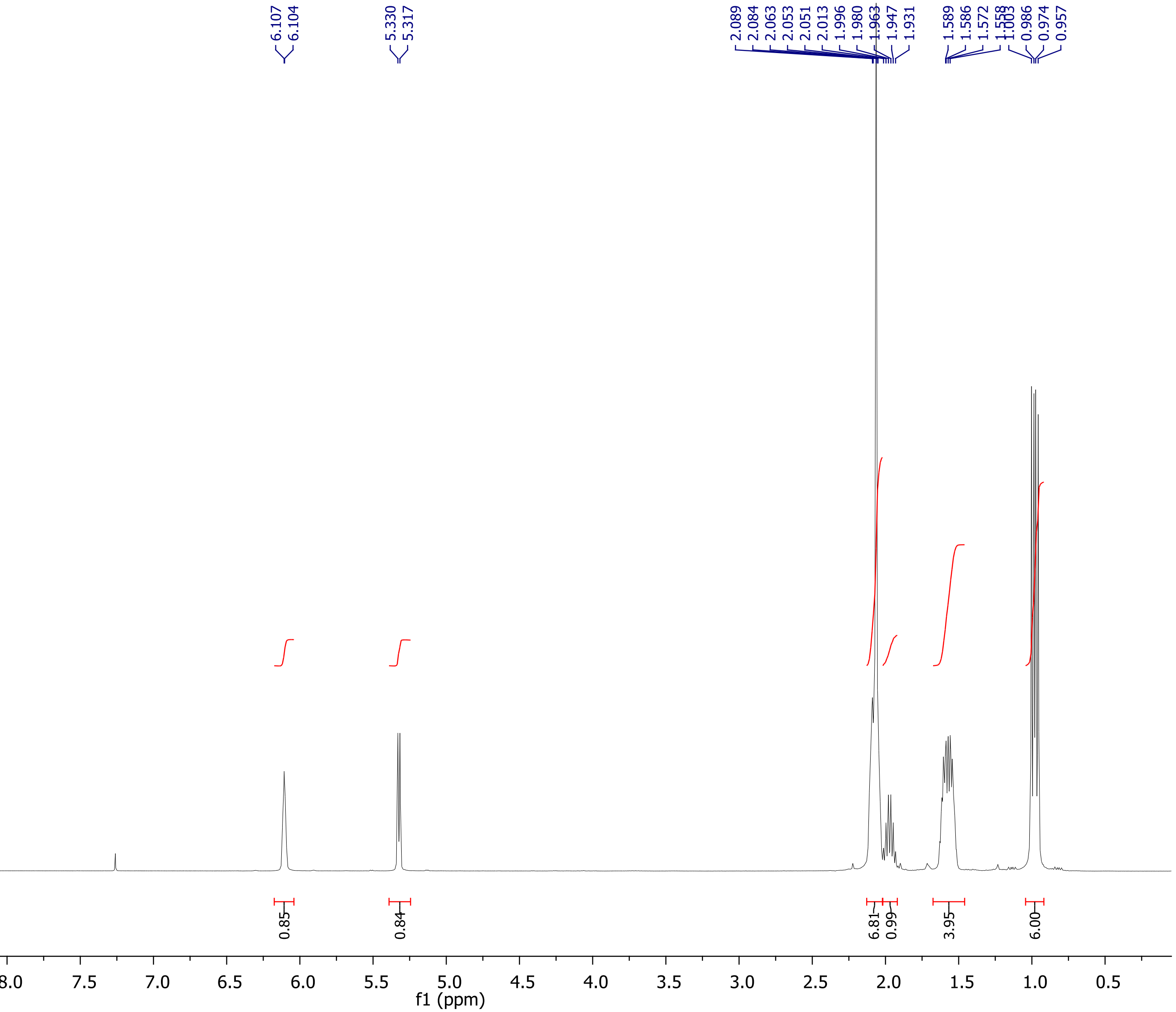

^{1}H NMR ($CDCl_3$, 400 MHz) of compound **21**

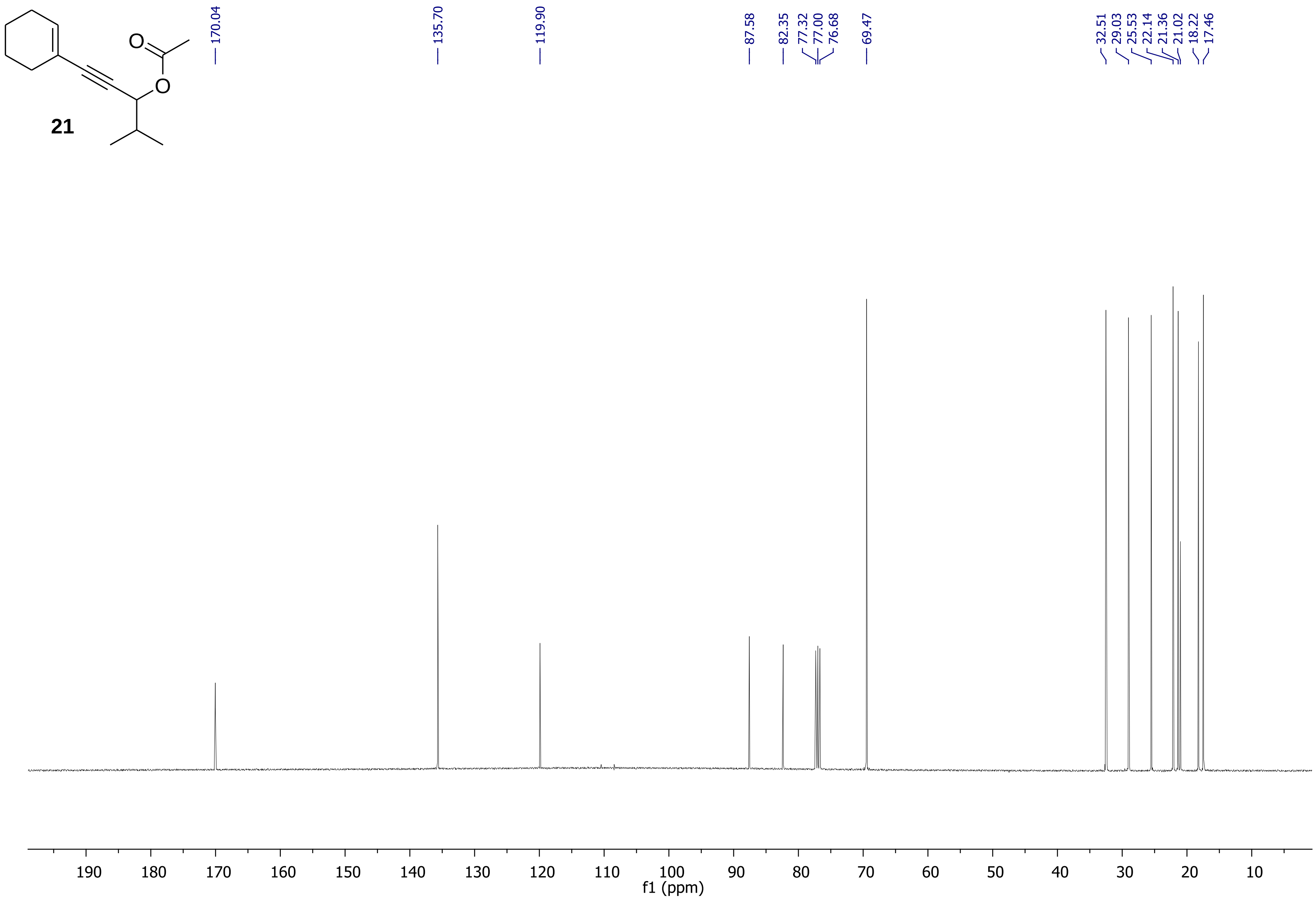

$^{13}C\{^{1}H\}$ NMR ($CDCl_3$, 100.4 MHz) of compound **21**

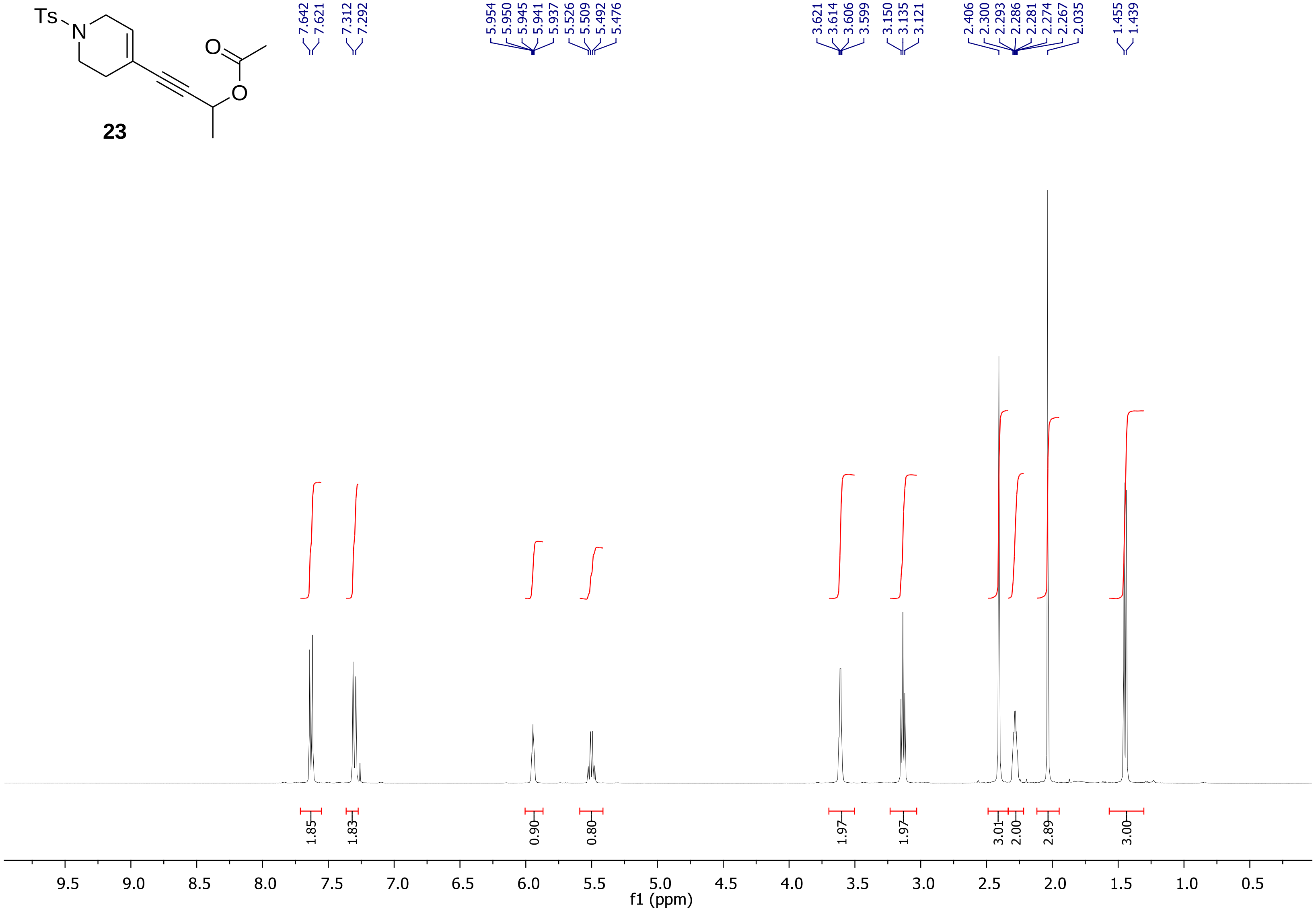

23

^{1}H NMR ($CDCl_3$, 400 MHz) of compound **23**

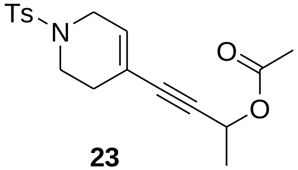

23

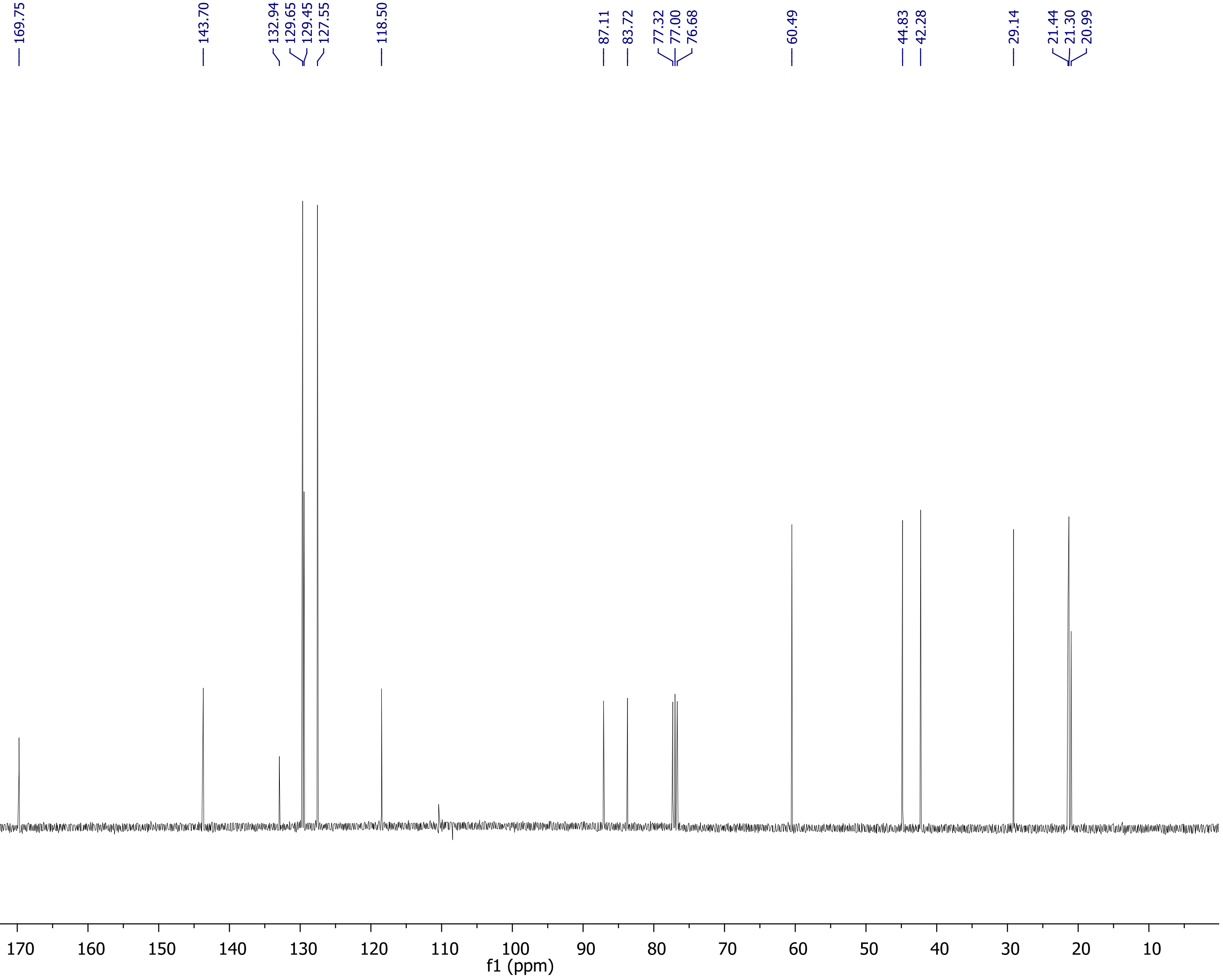

$^{13}C\{^{1}H\}$ NMR ($CDCl_3$, 100.4 MHz) of compound **23**

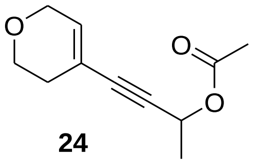

24

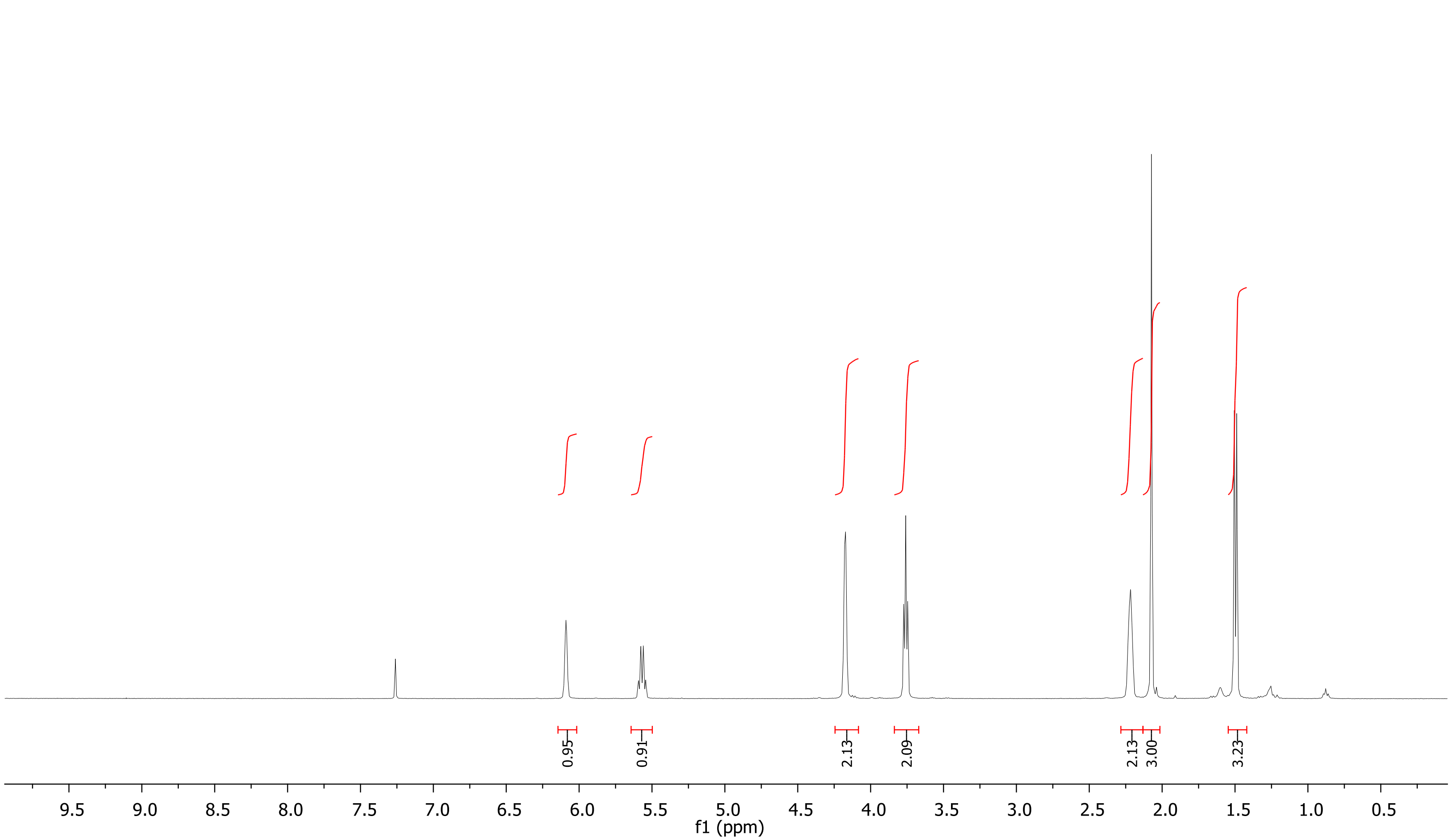

^{1}H NMR ($CDCl_3$, 400 MHz) of compound **24**

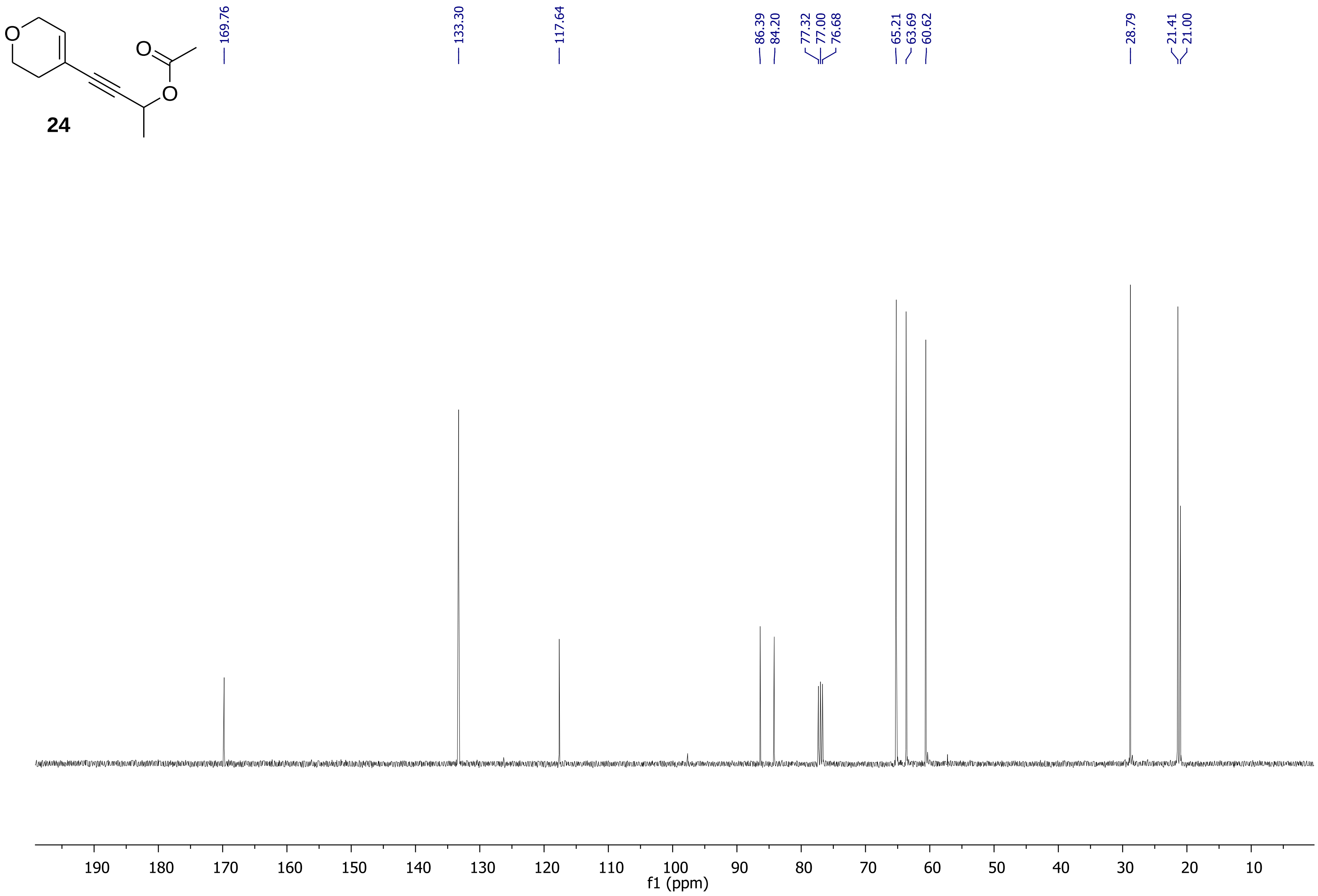

$^{13}C\{^{1}H\}$ NMR ($CDCl_3$, 100.4 MHz) of compound **24**

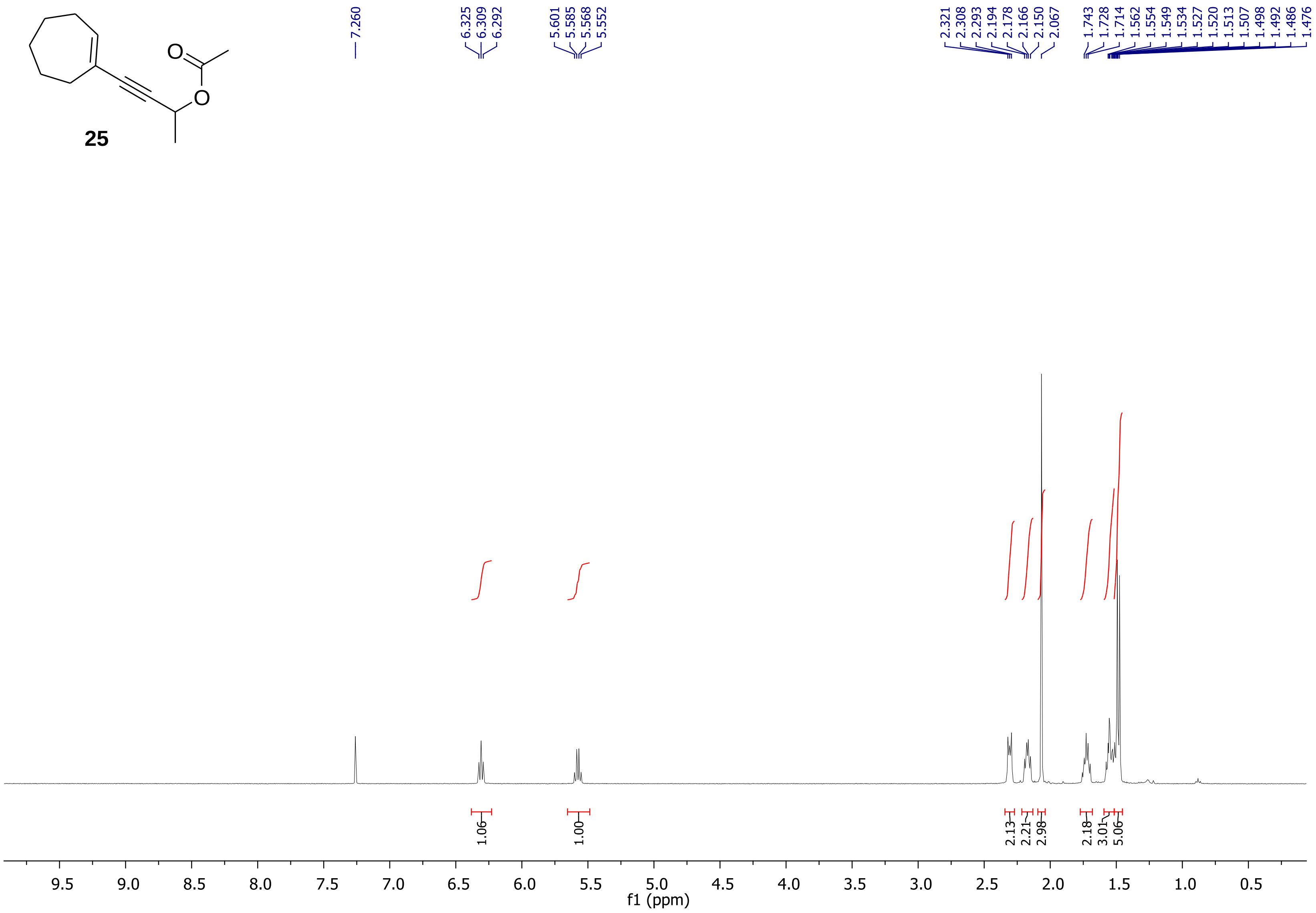

^{1}H NMR ($CDCl_3$, 400 MHz) of compound **25**

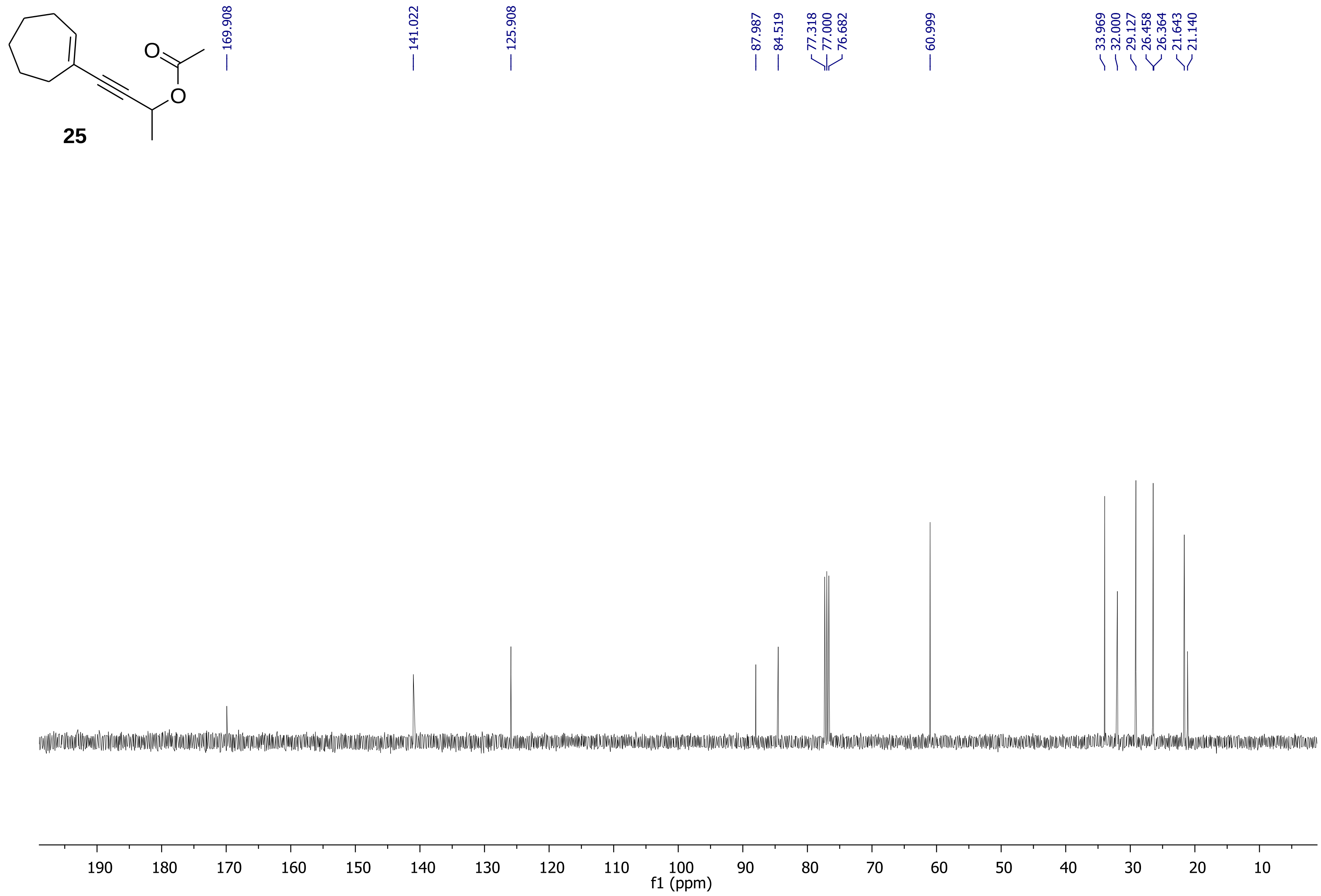

$^{13}C\{^1H\}$ NMR ($CDCl_3$, 100.4 MHz) of compound **25**

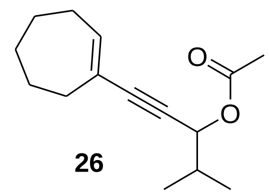

26

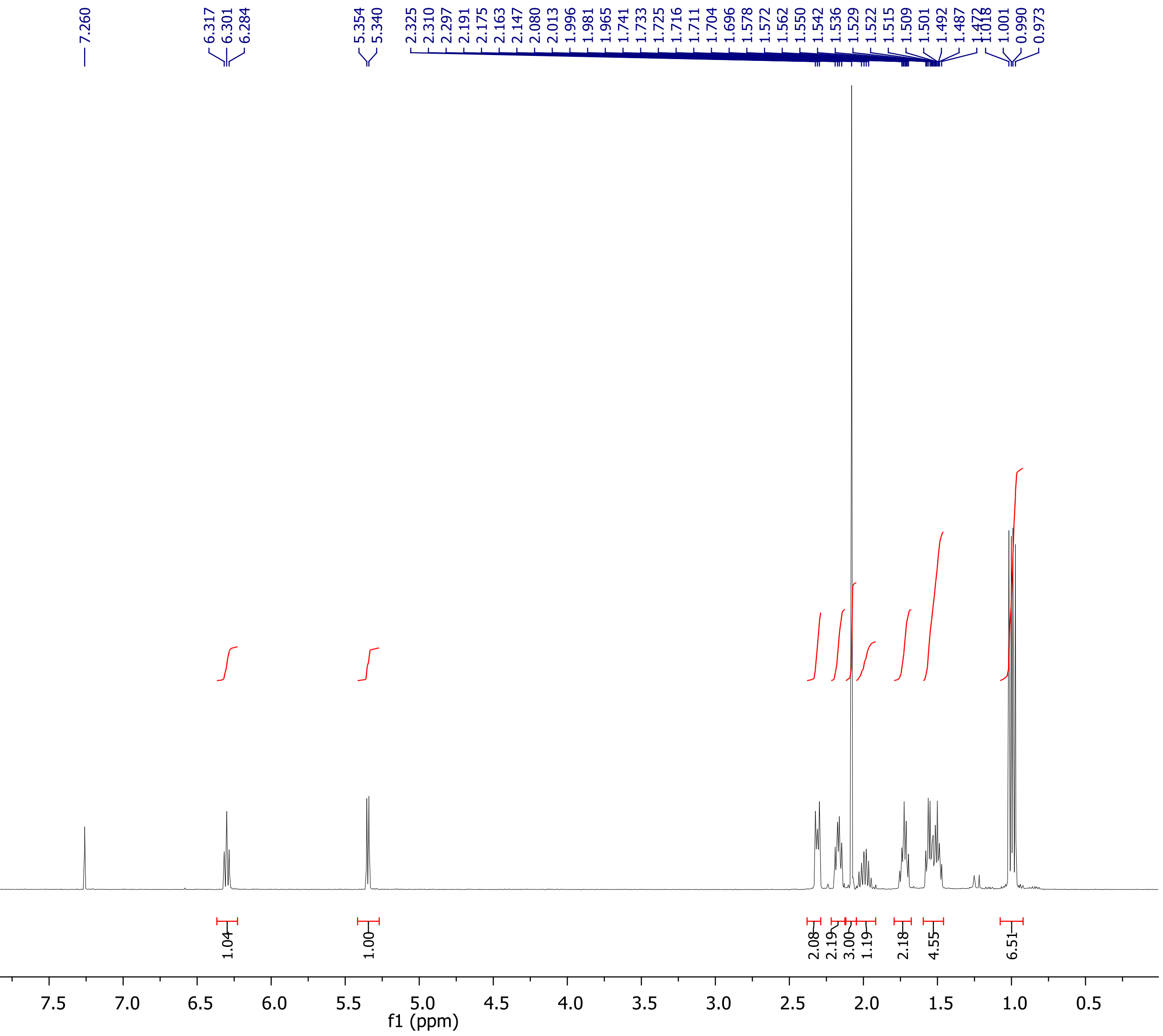

^{1}H NMR ($CDCl_3$, 400 MHz) of compound **26**

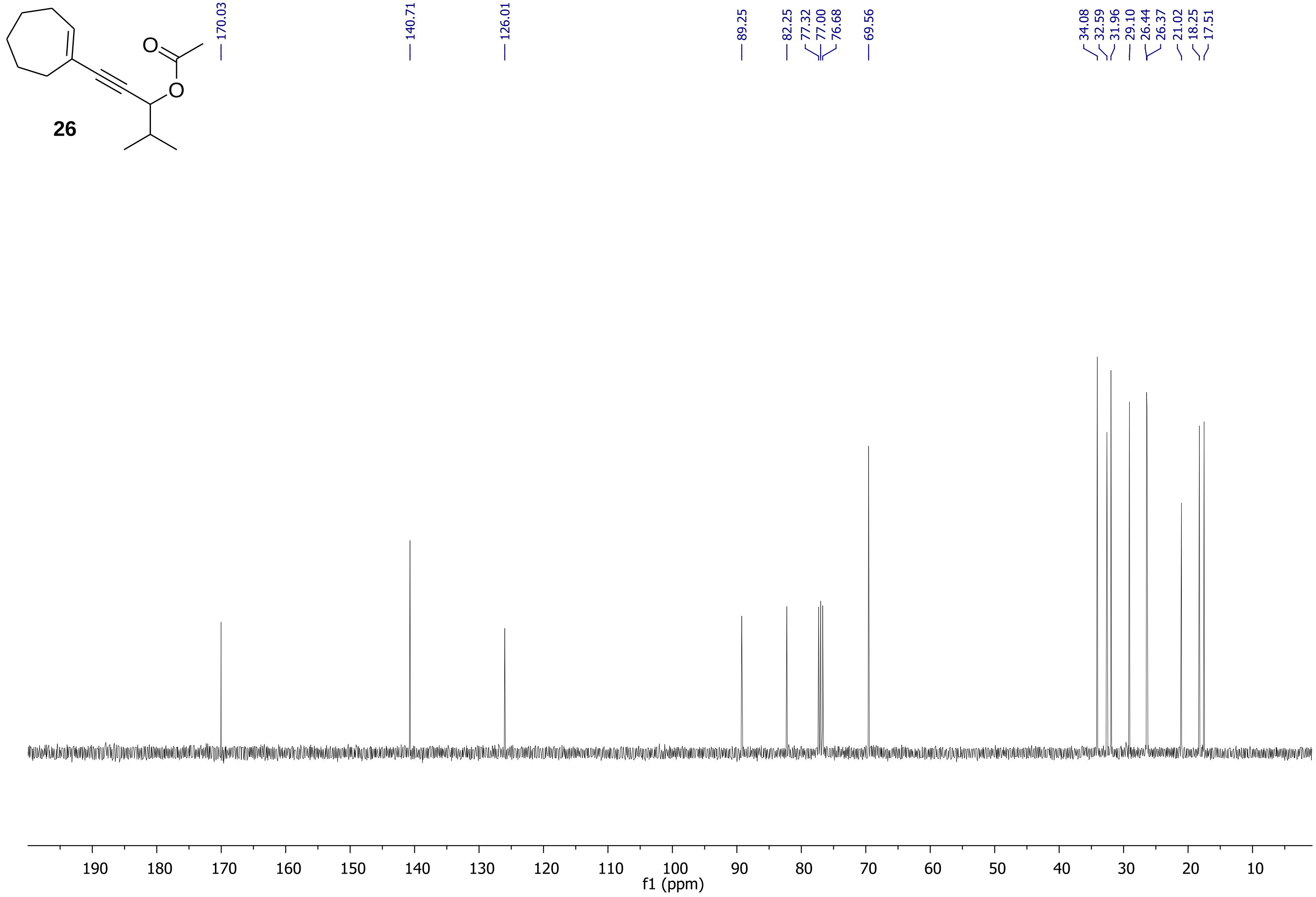

$^{13}C\{^{1}H\}$ NMR ($CDCl_3$, 100.4 MHz) of compound **26**

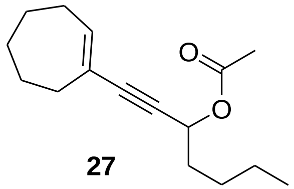

27

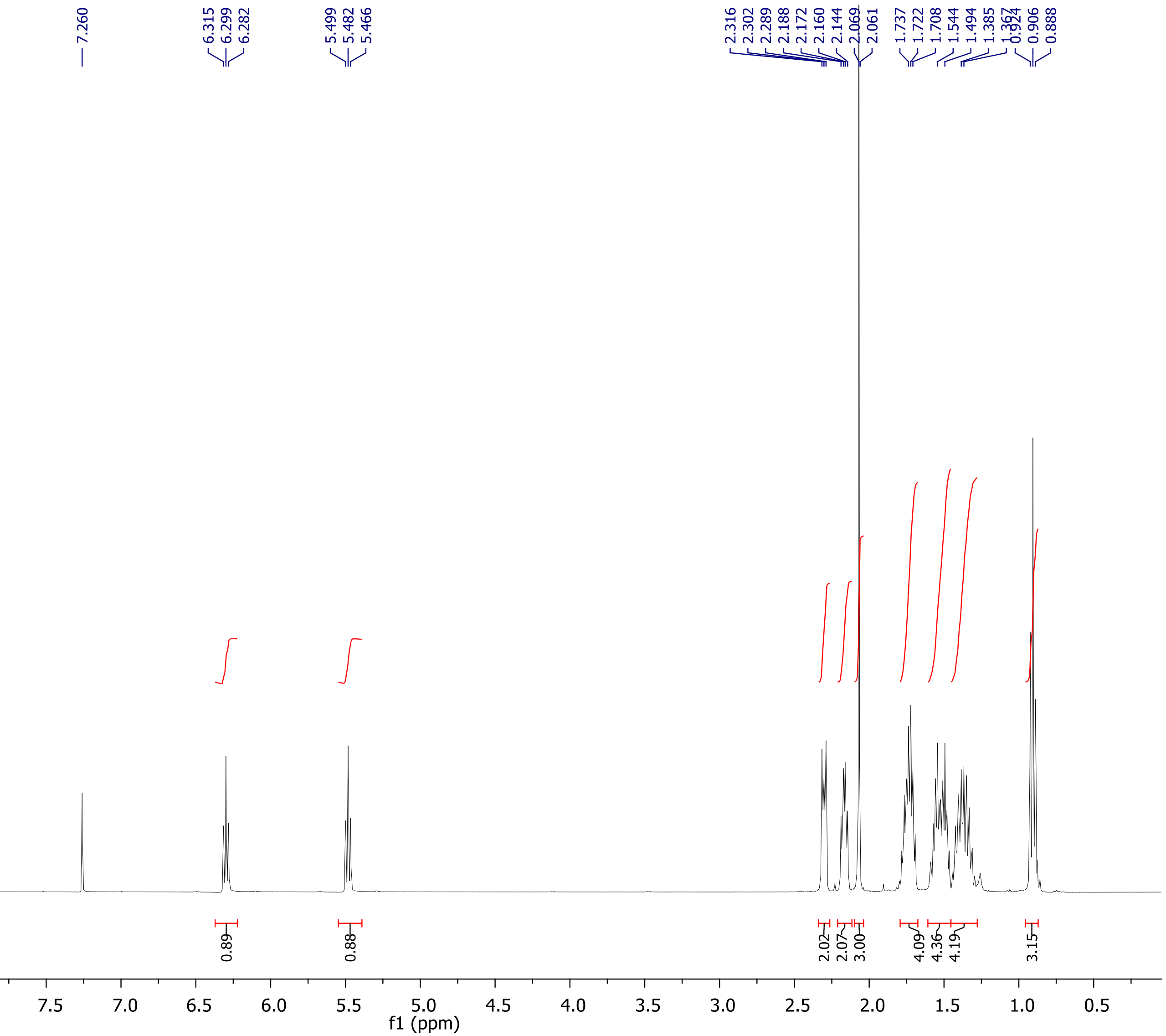

^{1}H NMR ($CDCl_3$, 400 MHz) of compound **27**

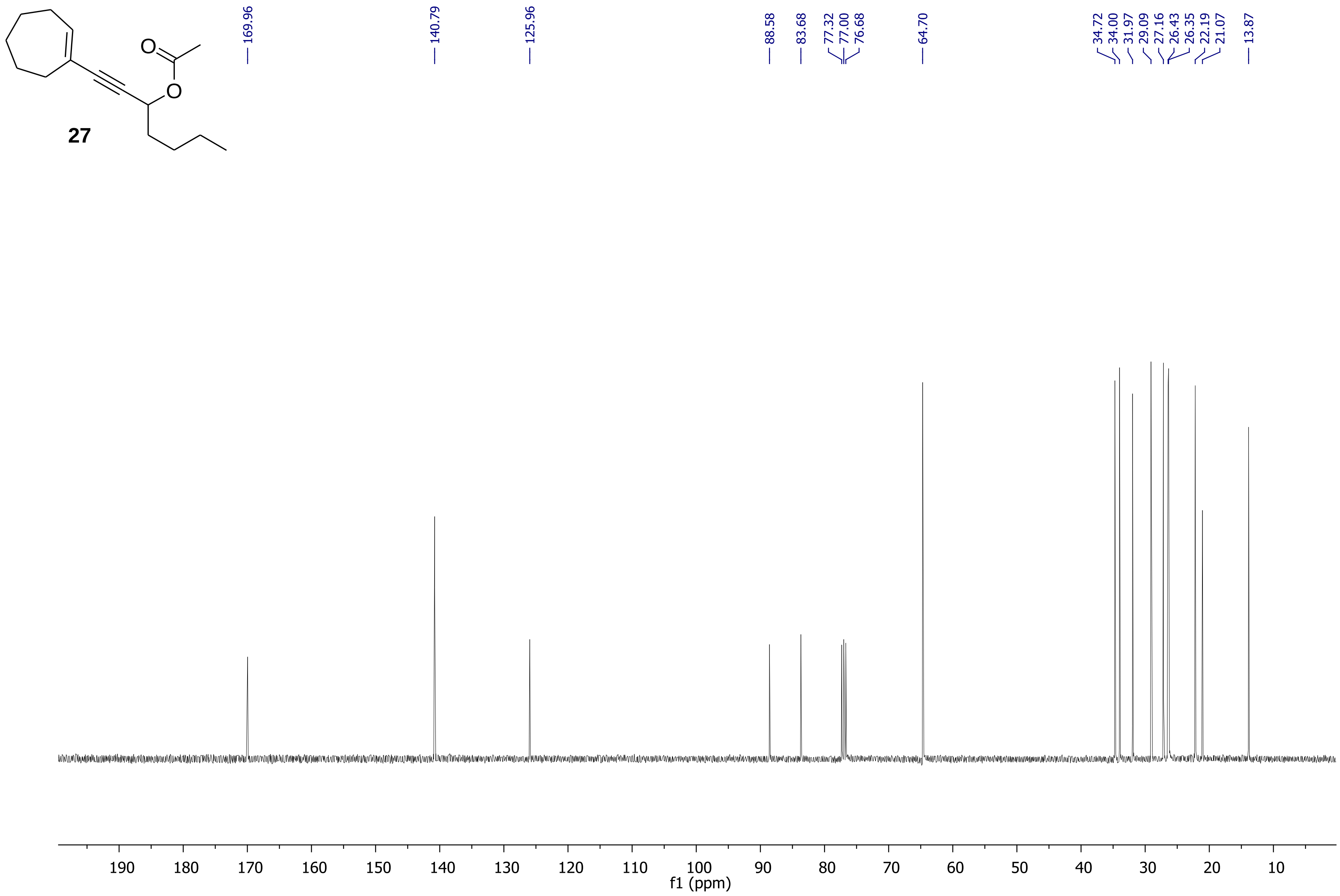

$^{13}C\{^{1}H\}$ NMR ($CDCl_3$, 100.4 MHz) of compound **27**

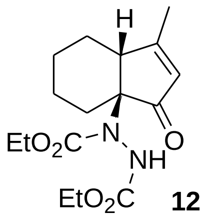

12

3.3 : 1 mixture of rotamers

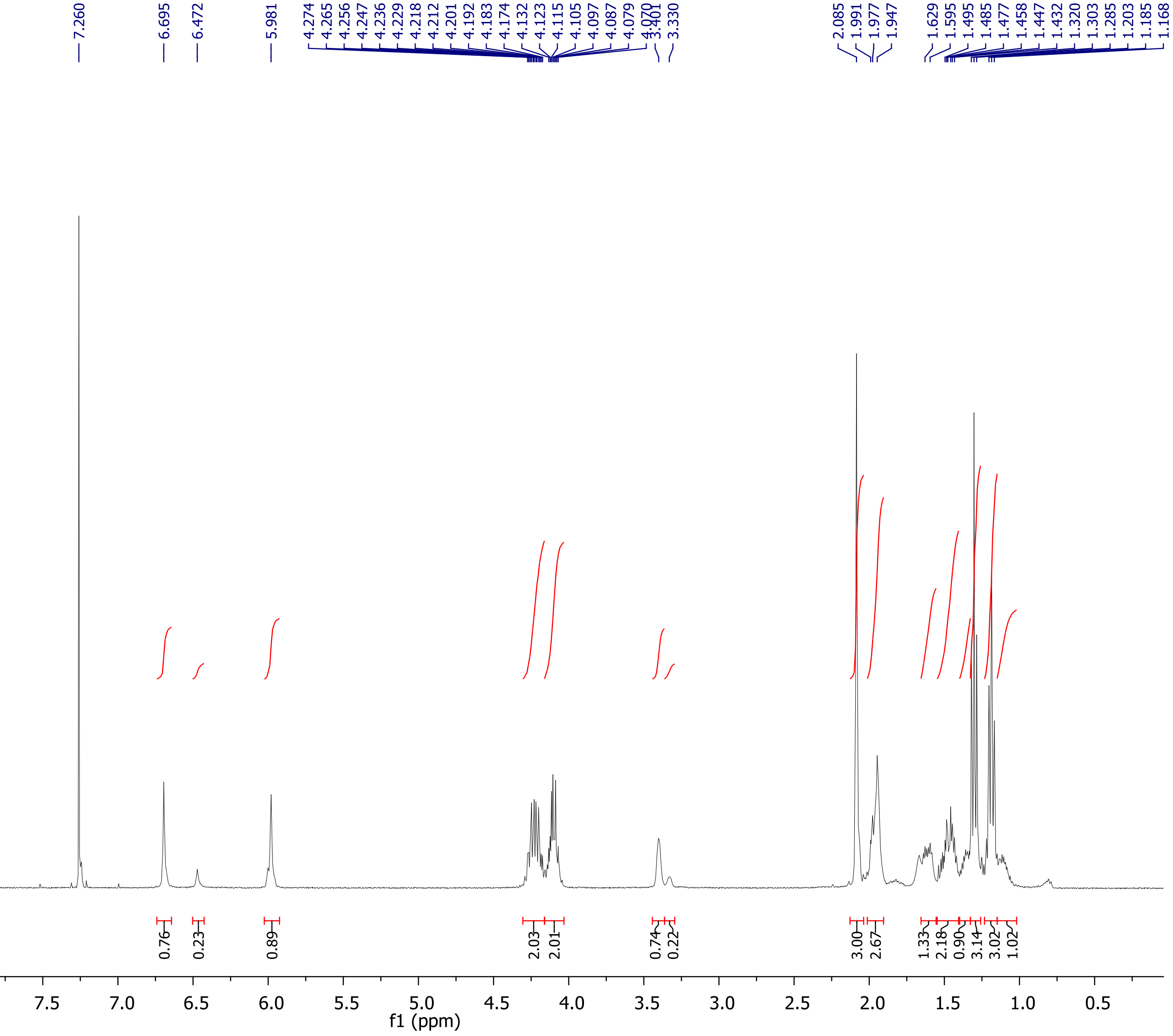

^{1}H NMR ($CDCl_3$, 400 MHz) of compound **12**

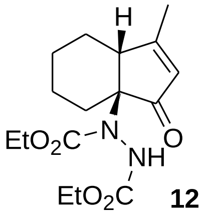

12

mixture of rotamers

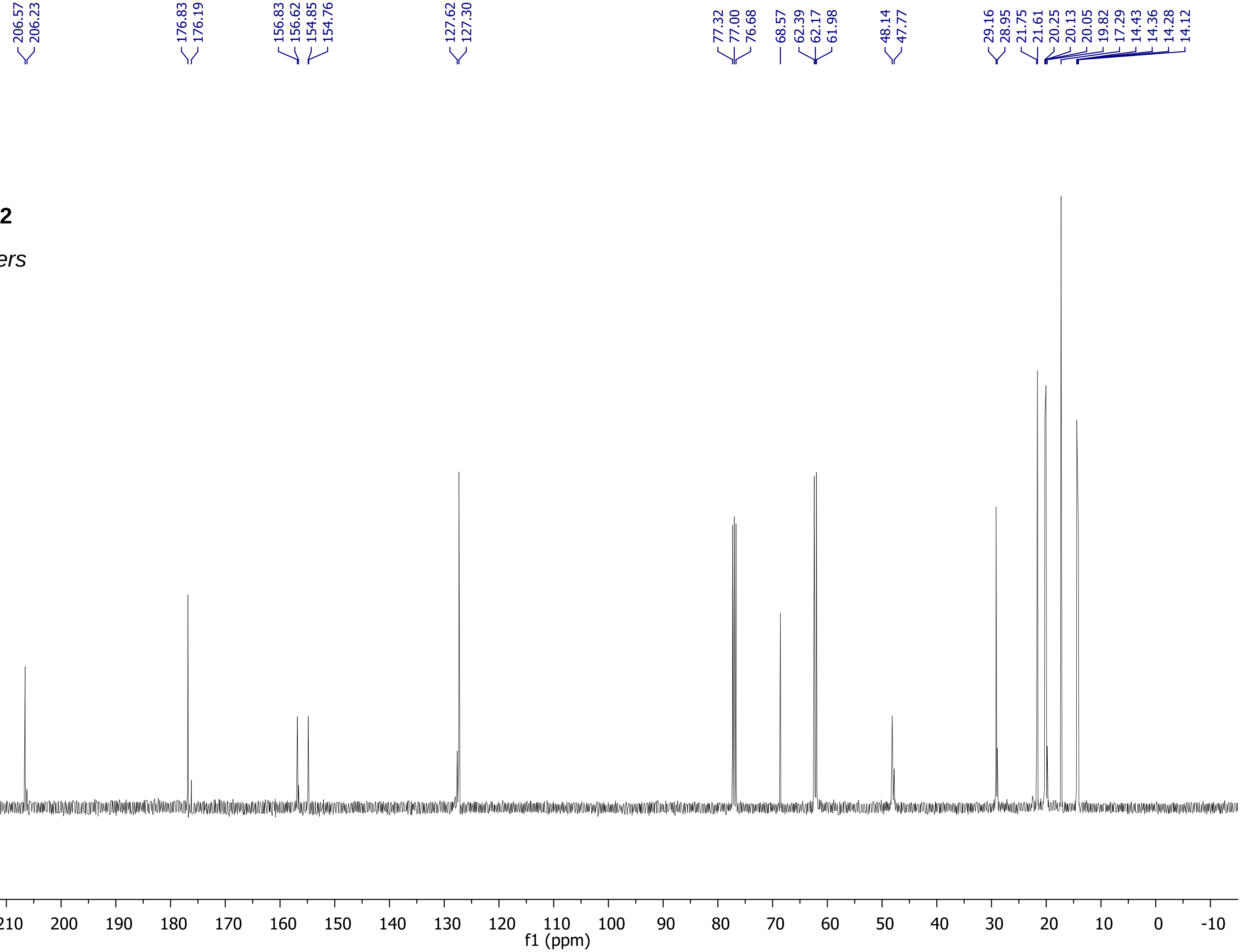

$^{13}C\{^1H\}$ NMR ($CDCl_3$, 100.4 MHz) of compound **12**

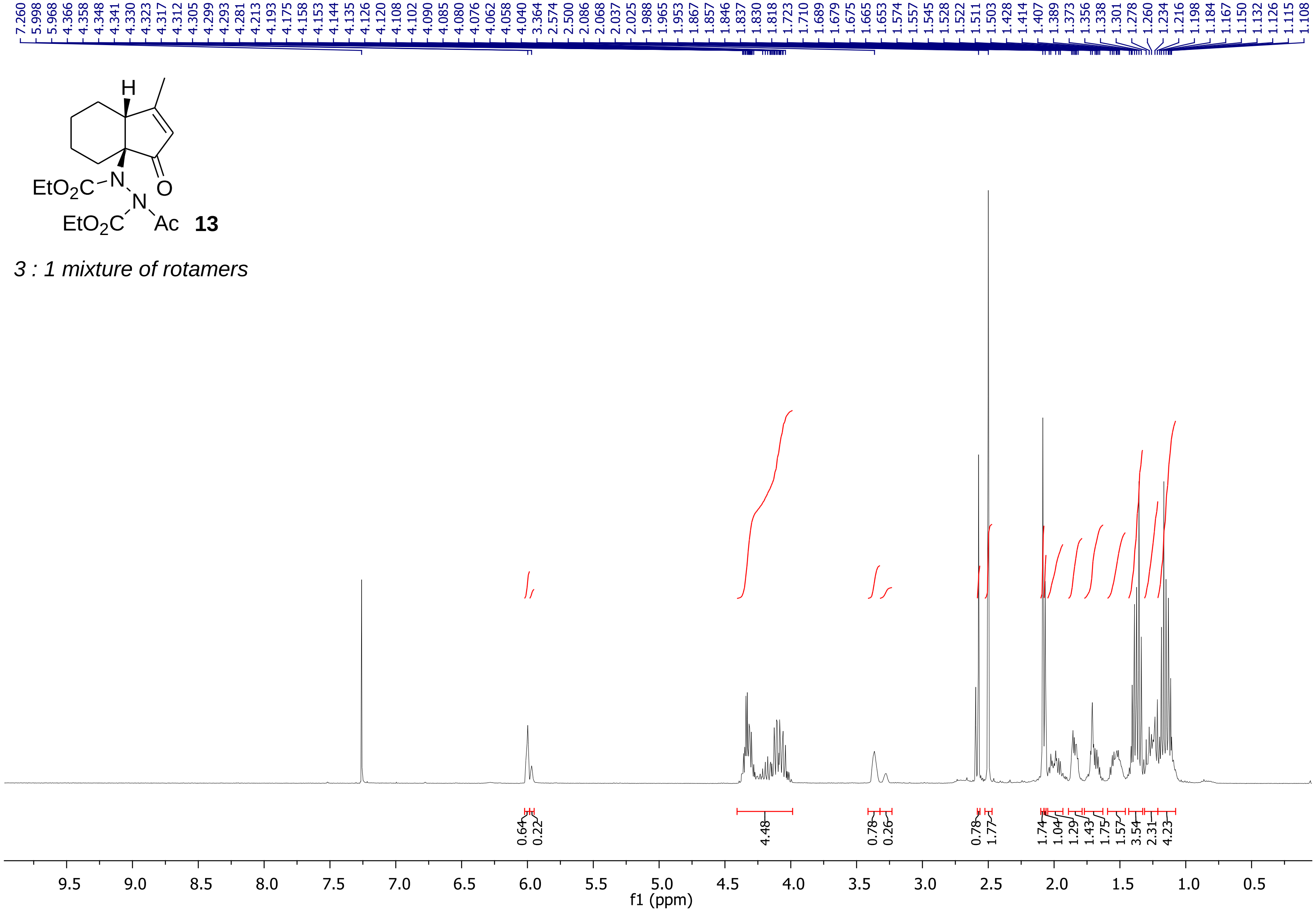

^{1}H NMR ($CDCl_3$, 400 MHz) of compound **13**

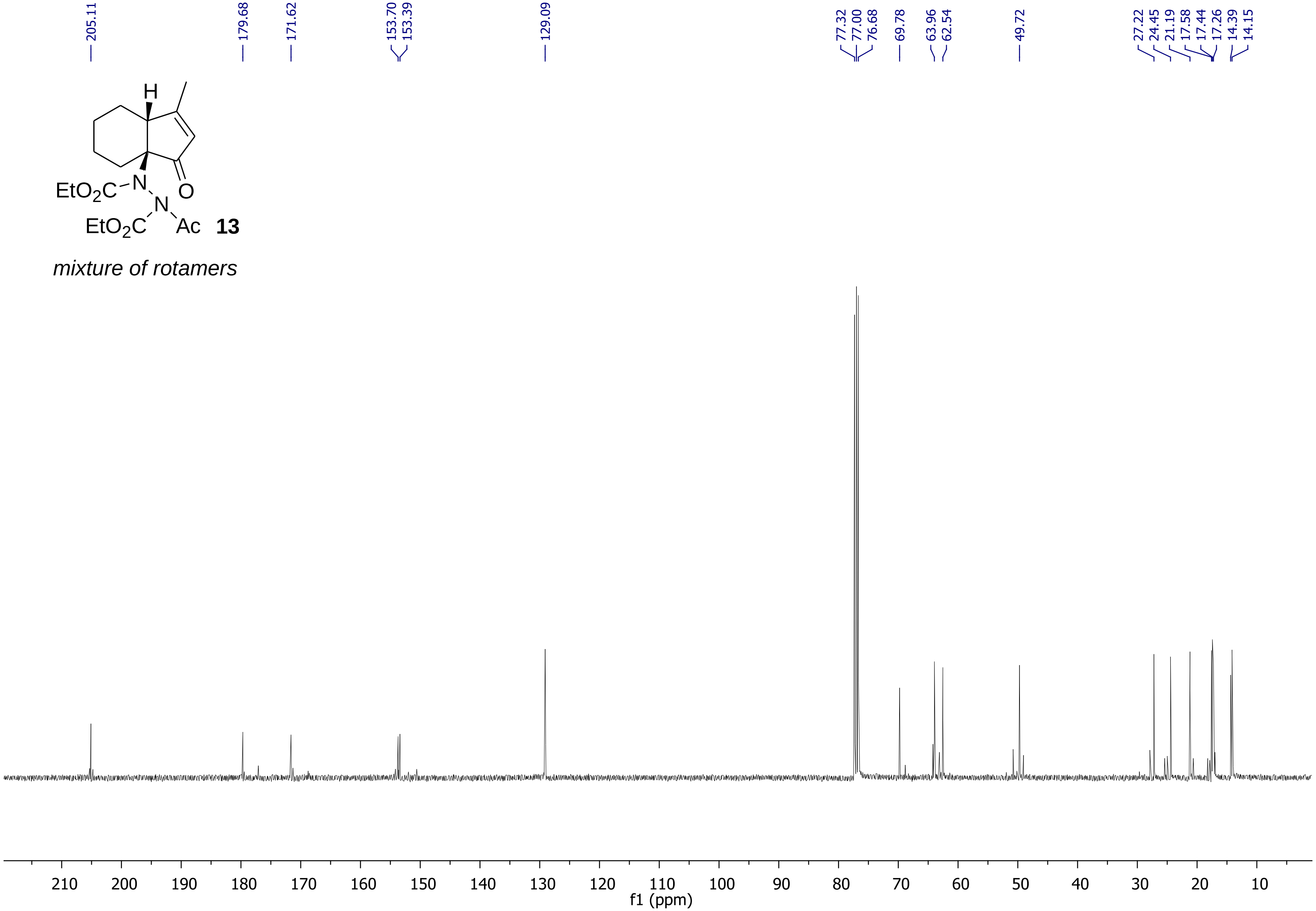

$^{13}C\{^{1}H\}$ NMR ($CDCl_3$, 100.4 MHz) of compound **13**

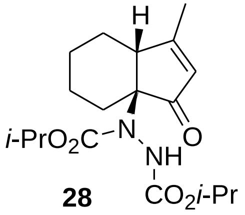

28

2.9 : 1 mixture of rotamers

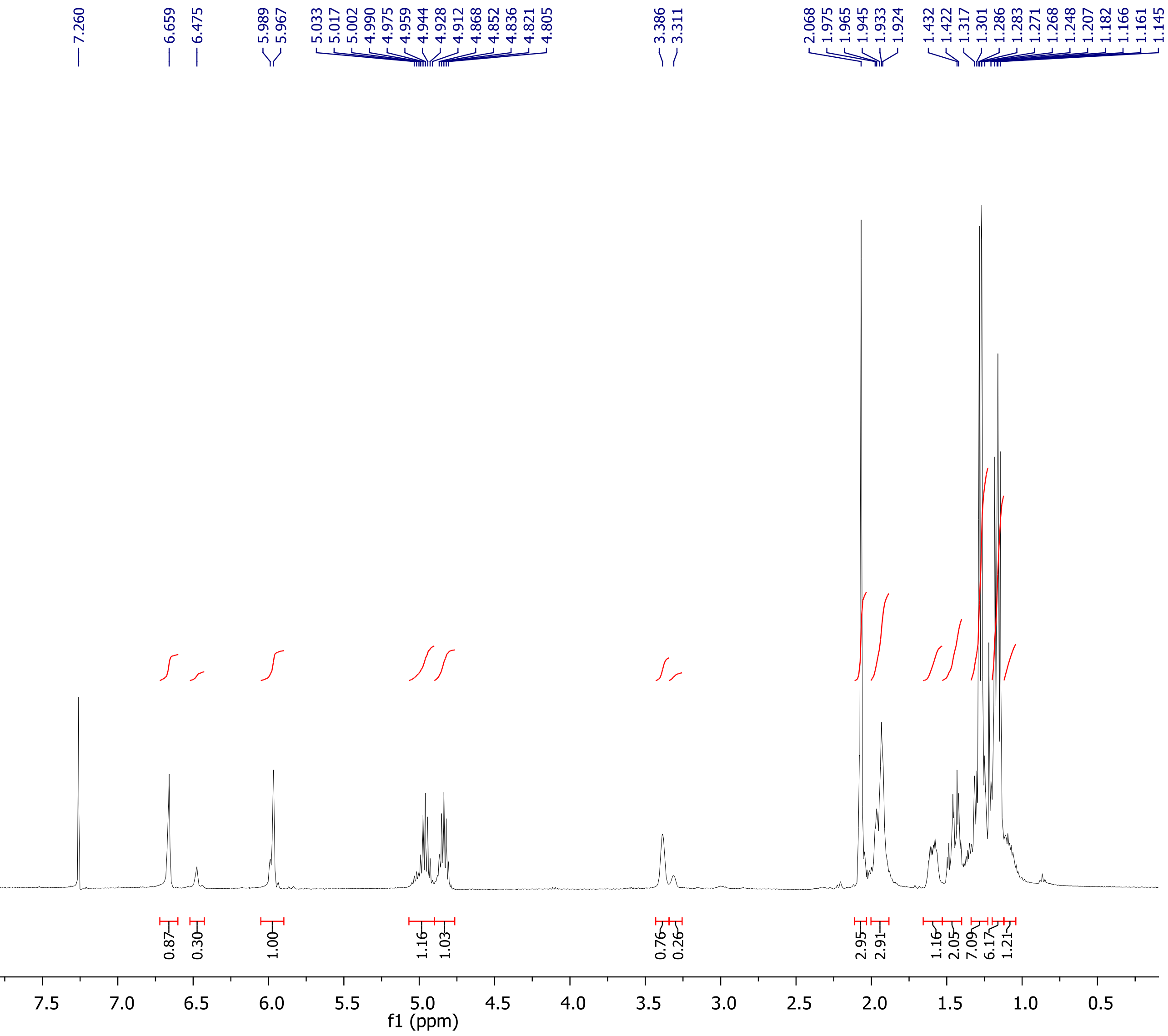

^{1}H NMR ($CDCl_3$, 400 MHz) of compound **28**

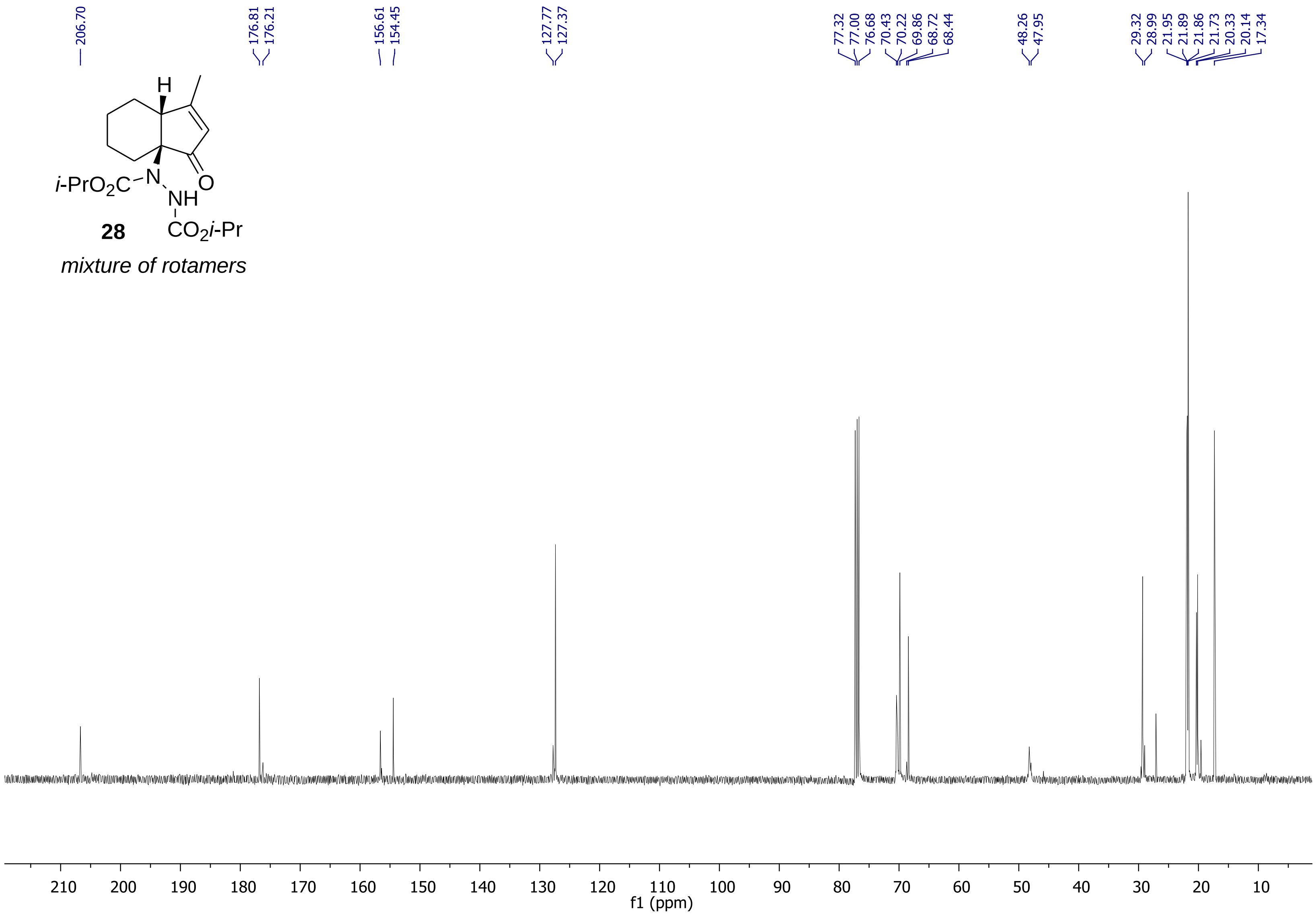

$^{13}C\{^{1}H\}$ NMR ($CDCl_3$, 100.4 MHz) of compound **28**

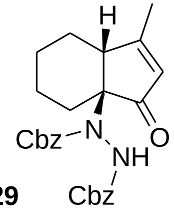

29

2.5 : 1 mixture of rotamers

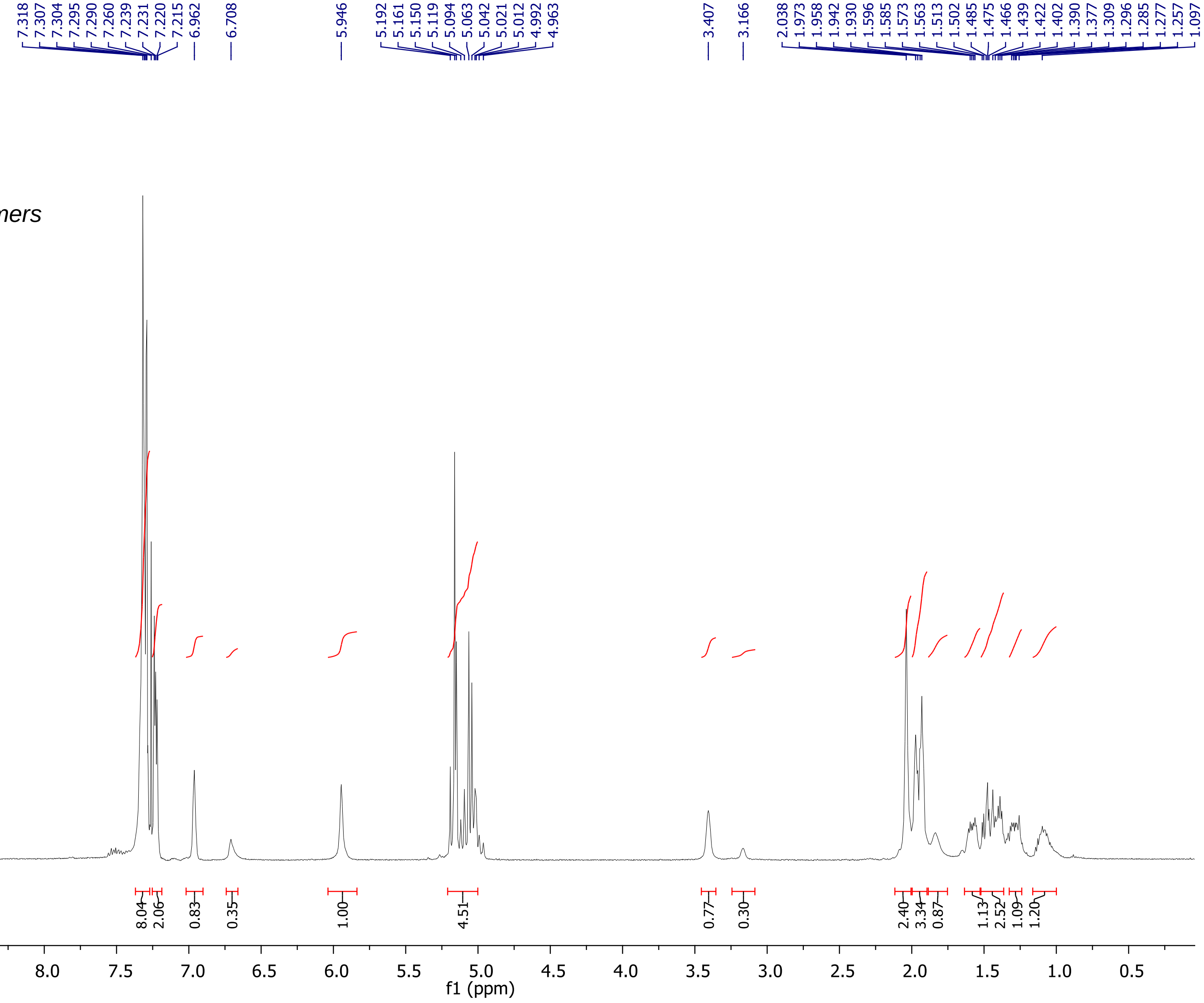

^{1}H NMR ($CDCl_3$, 400 MHz) of compound **29**

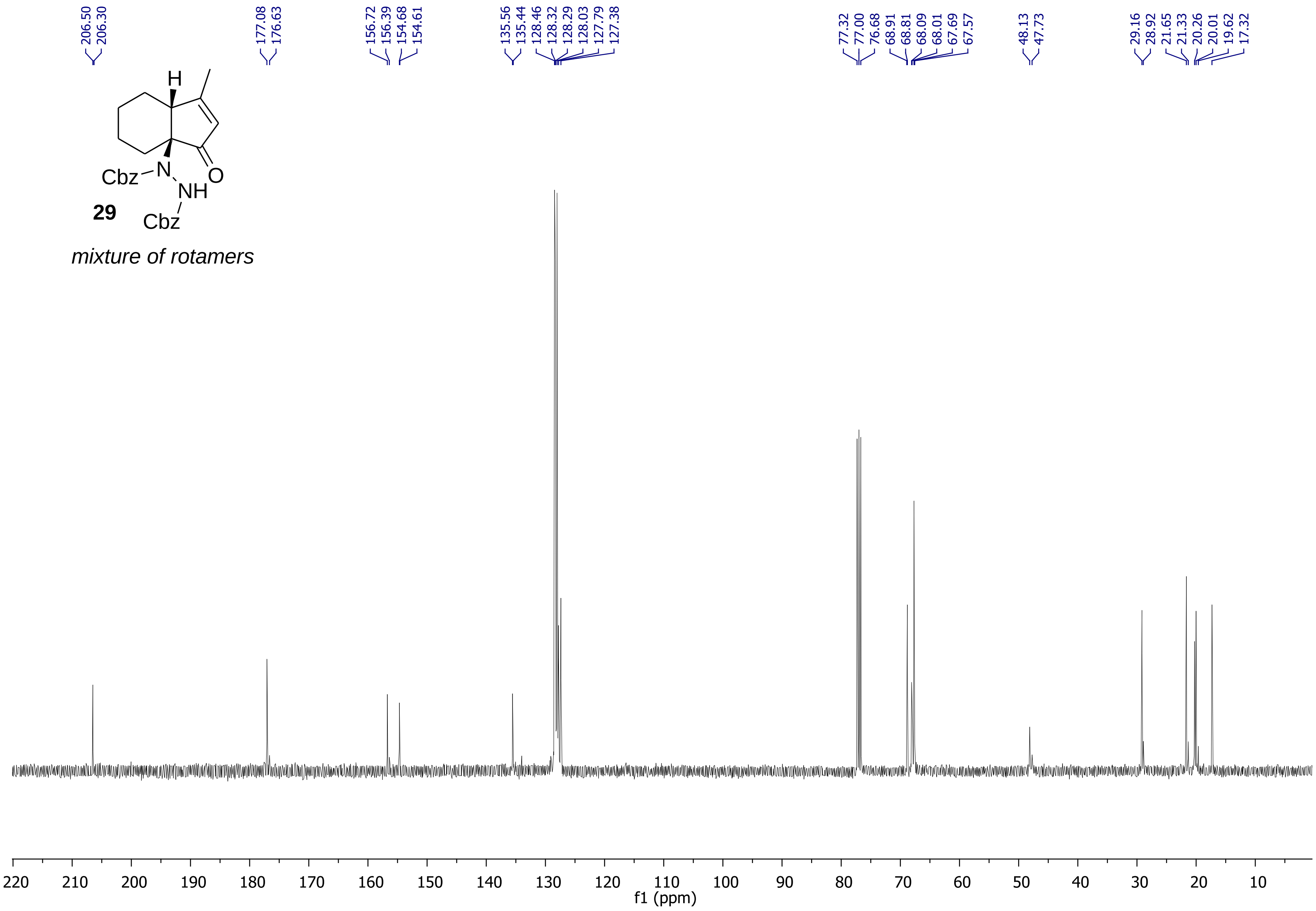

$^{13}C\{^{1}H\}$ NMR ($CDCl_3$, 100.4 MHz) of compound **29**

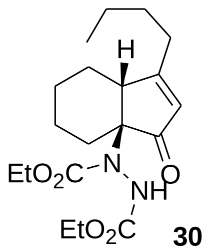

30

3.1 : 1 mixture of rotamers

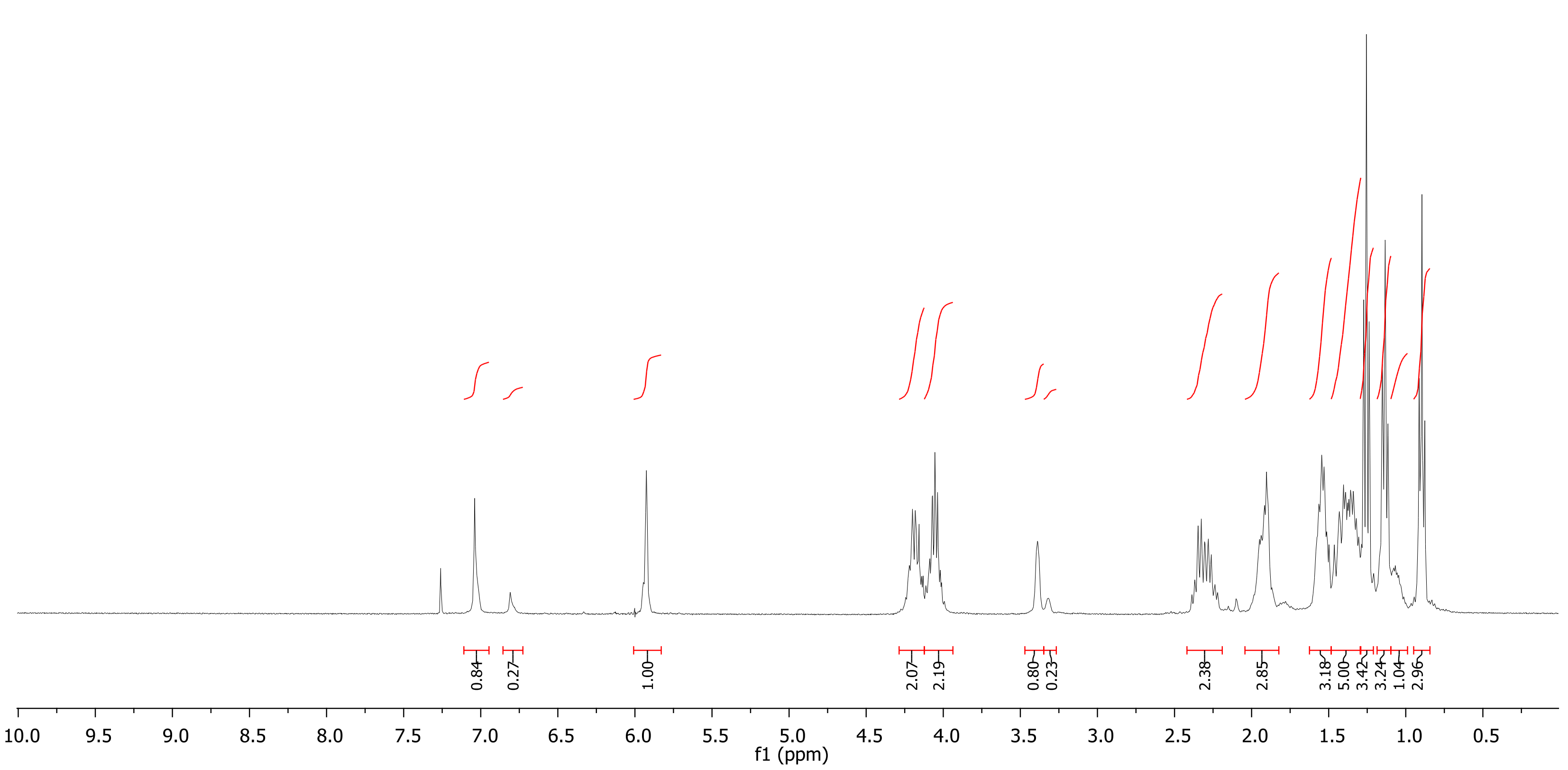

^{1}H NMR ($CDCl_3$, 400 MHz) of compound **30**

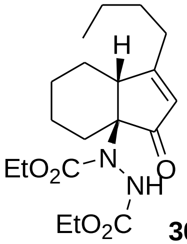

30

mixture of rotamers

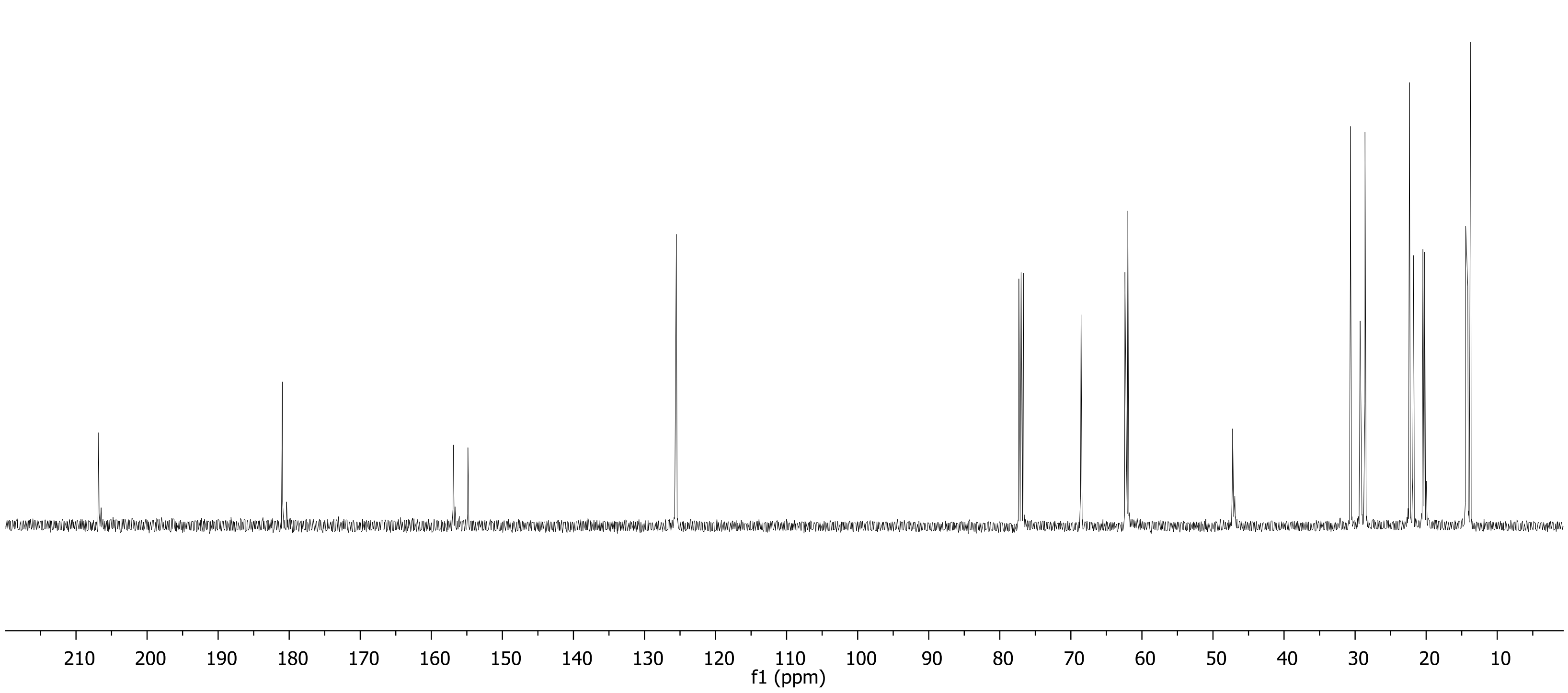

$^{13}C\{^1H\}$ NMR ($CDCl_3$, 100.4 MHz) of compound **30**

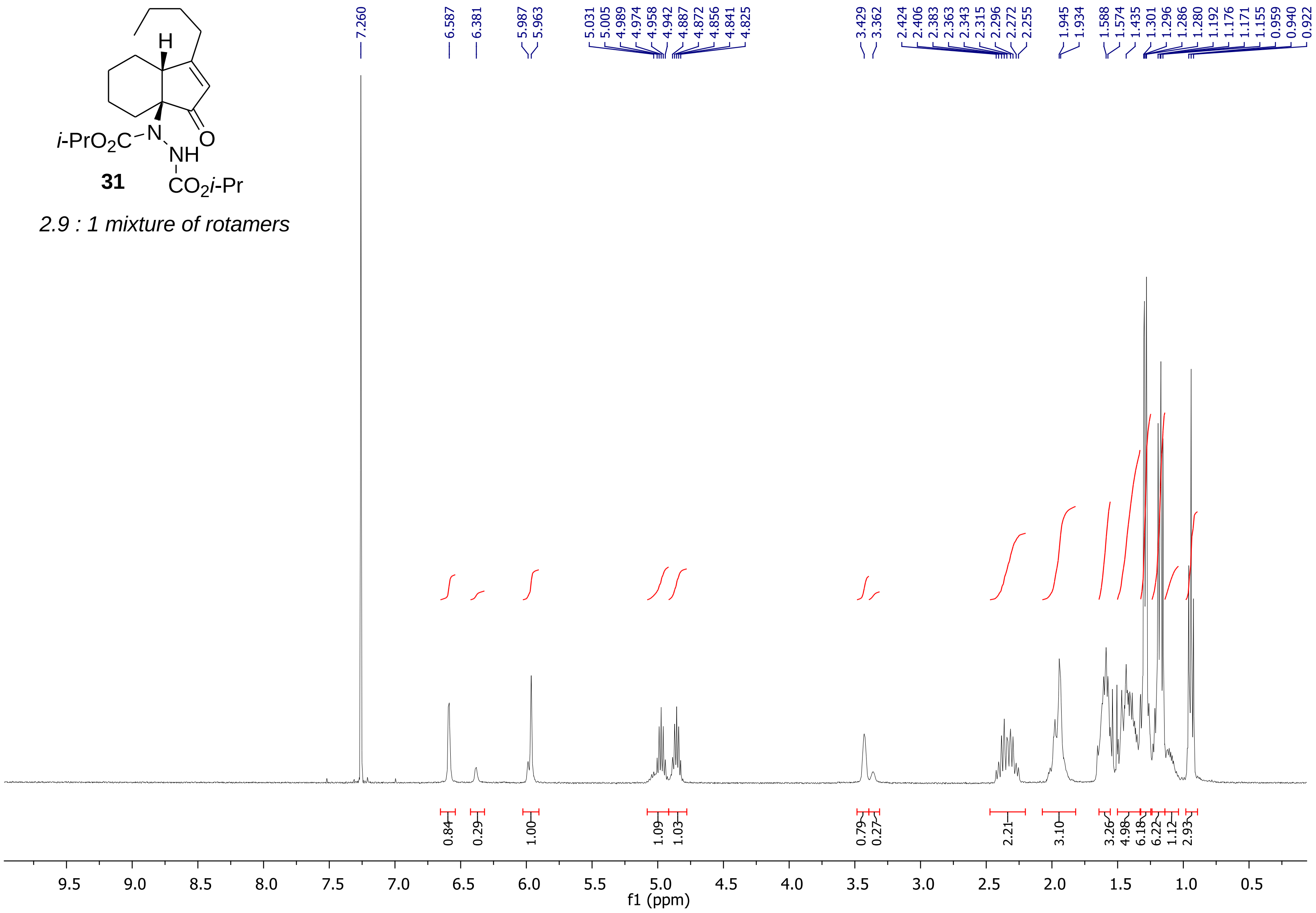

^{1}H NMR ($CDCl_3$, 400 MHz) of compound **31**

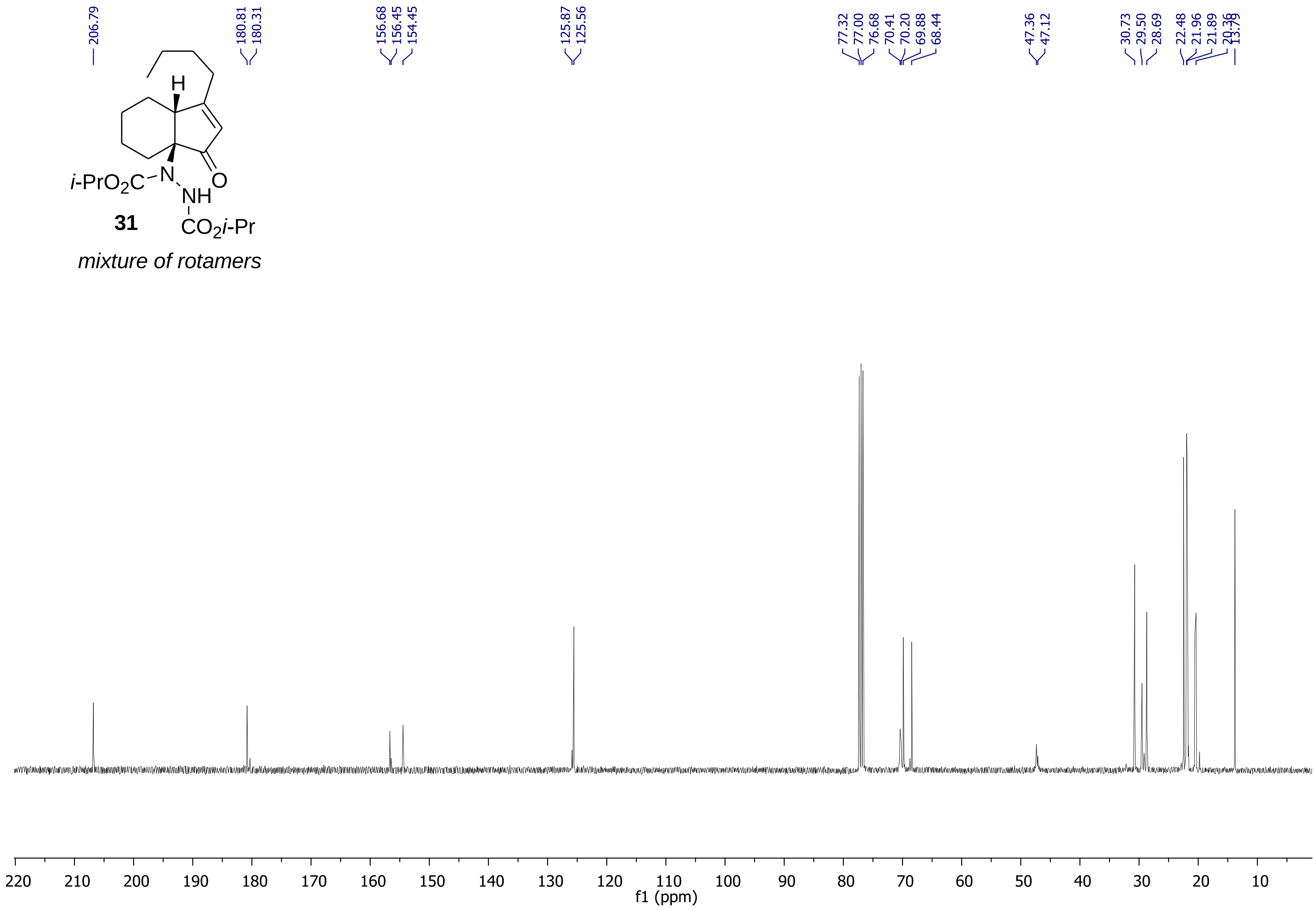

$^{13}C\{^{1}H\}$ NMR ($CDCl_3$, 100.4 MHz) of compound **31**

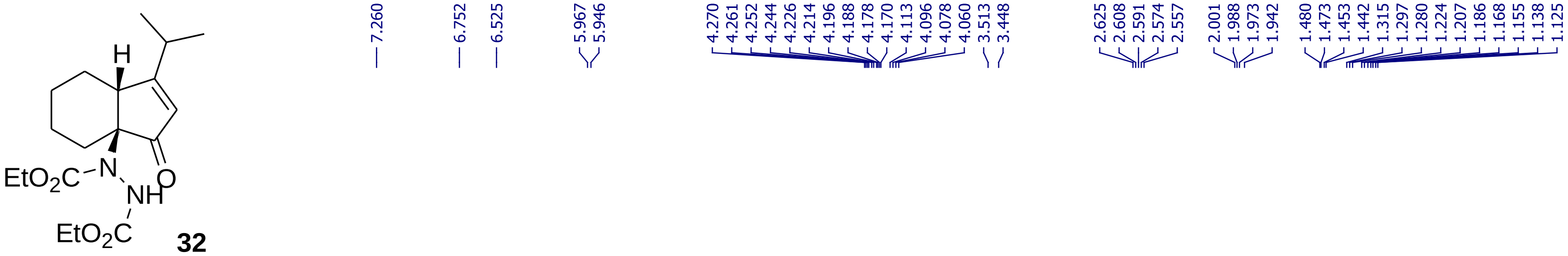

32

4.6 : 1 mixture of rotamers

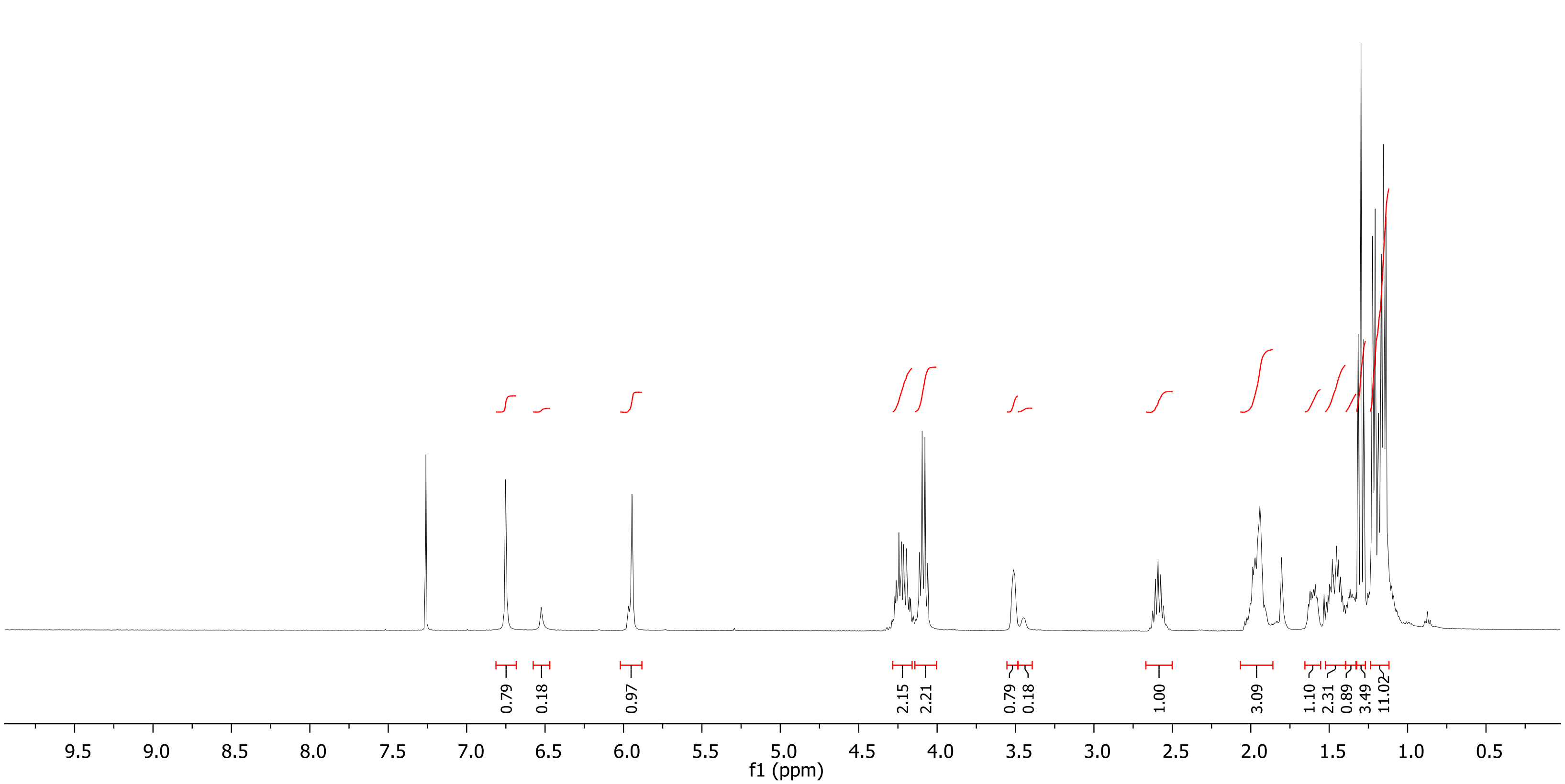

^{1}H NMR (CDCl$_3$, 400 MHz) of compound **32**

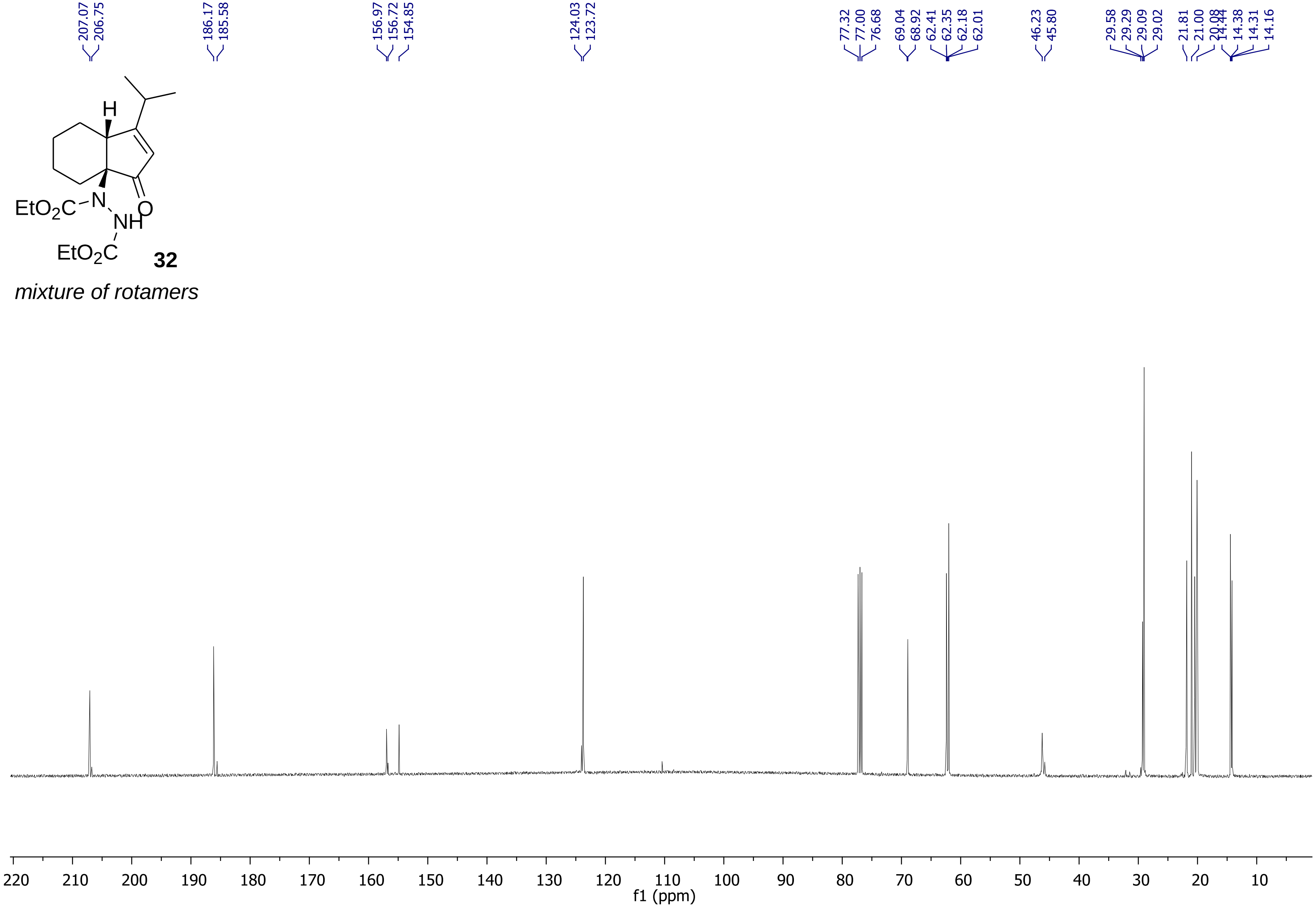

$^{13}C\{^{1}H\}$ NMR ($CDCl_3$, 100.4 MHz) of compound **32**

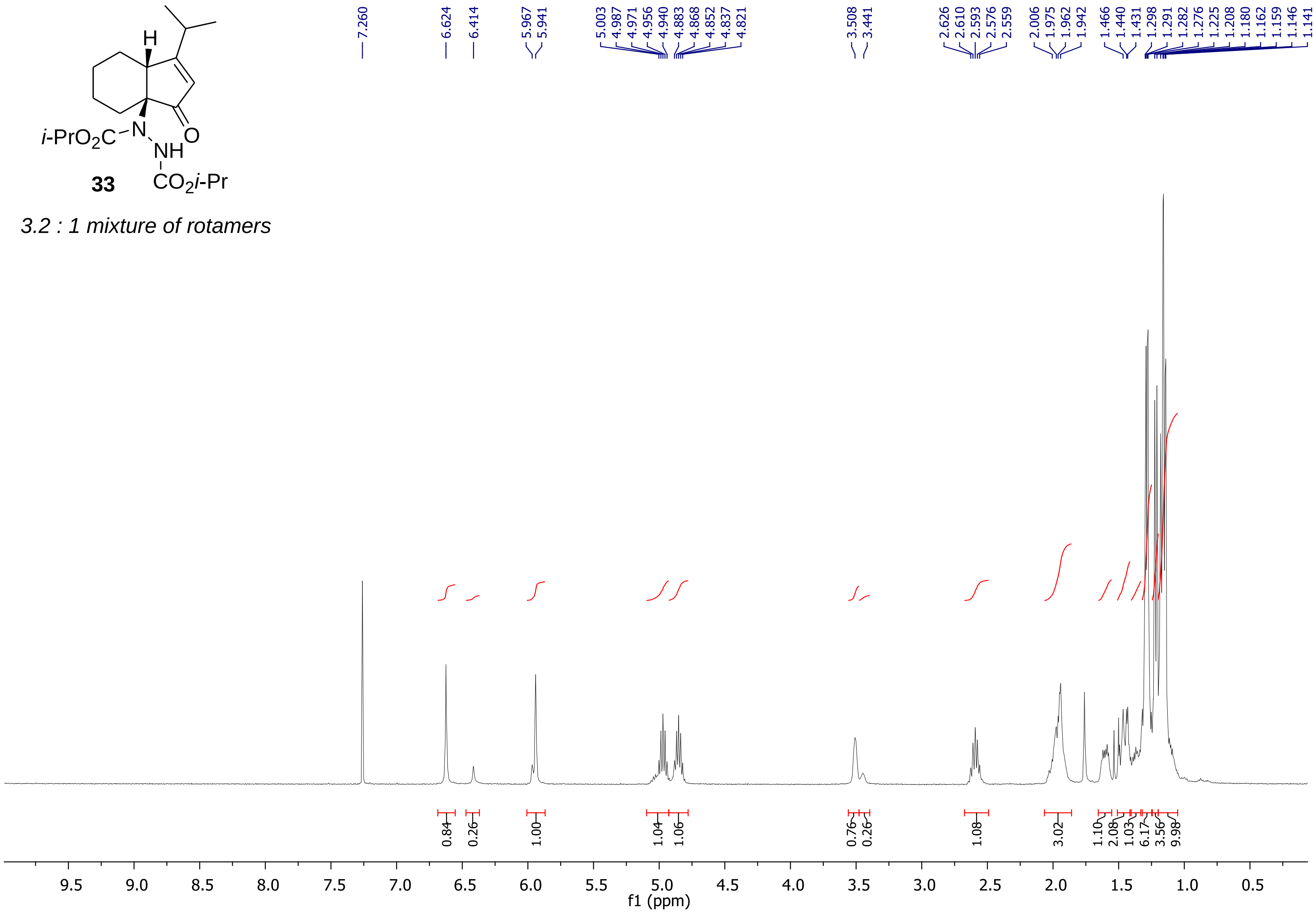

^{1}H NMR ($CDCl_3$, 400 MHz) of compound **33**

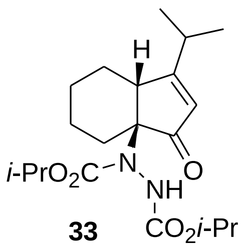

33

mixture of rotamers

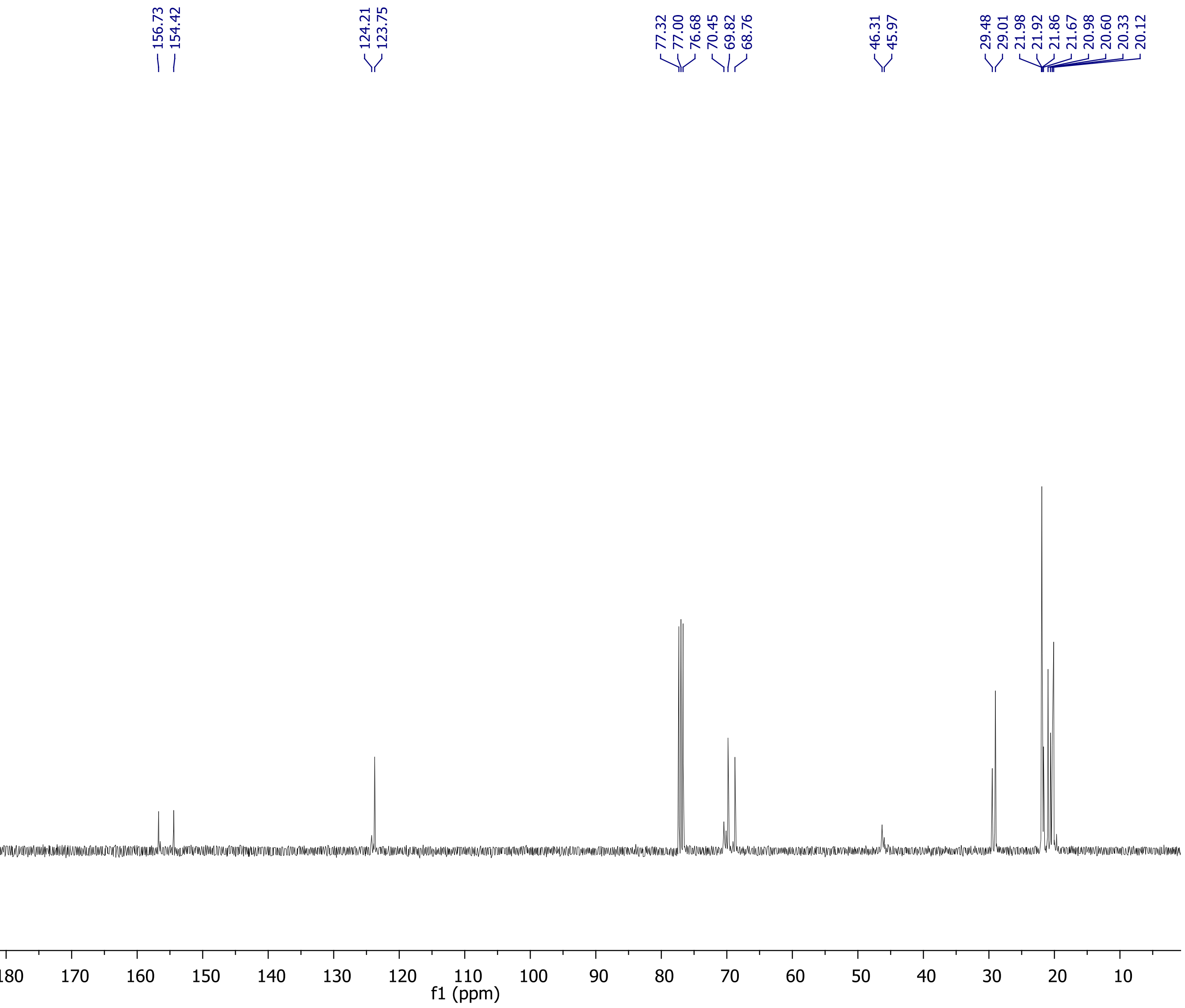

$^{13}C\{^{1}H\}$ NMR ($CDCl_3$, 100.4 MHz) of compound **33**

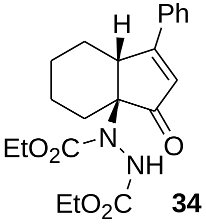

34

2.8 : 1 mixture of rotamers

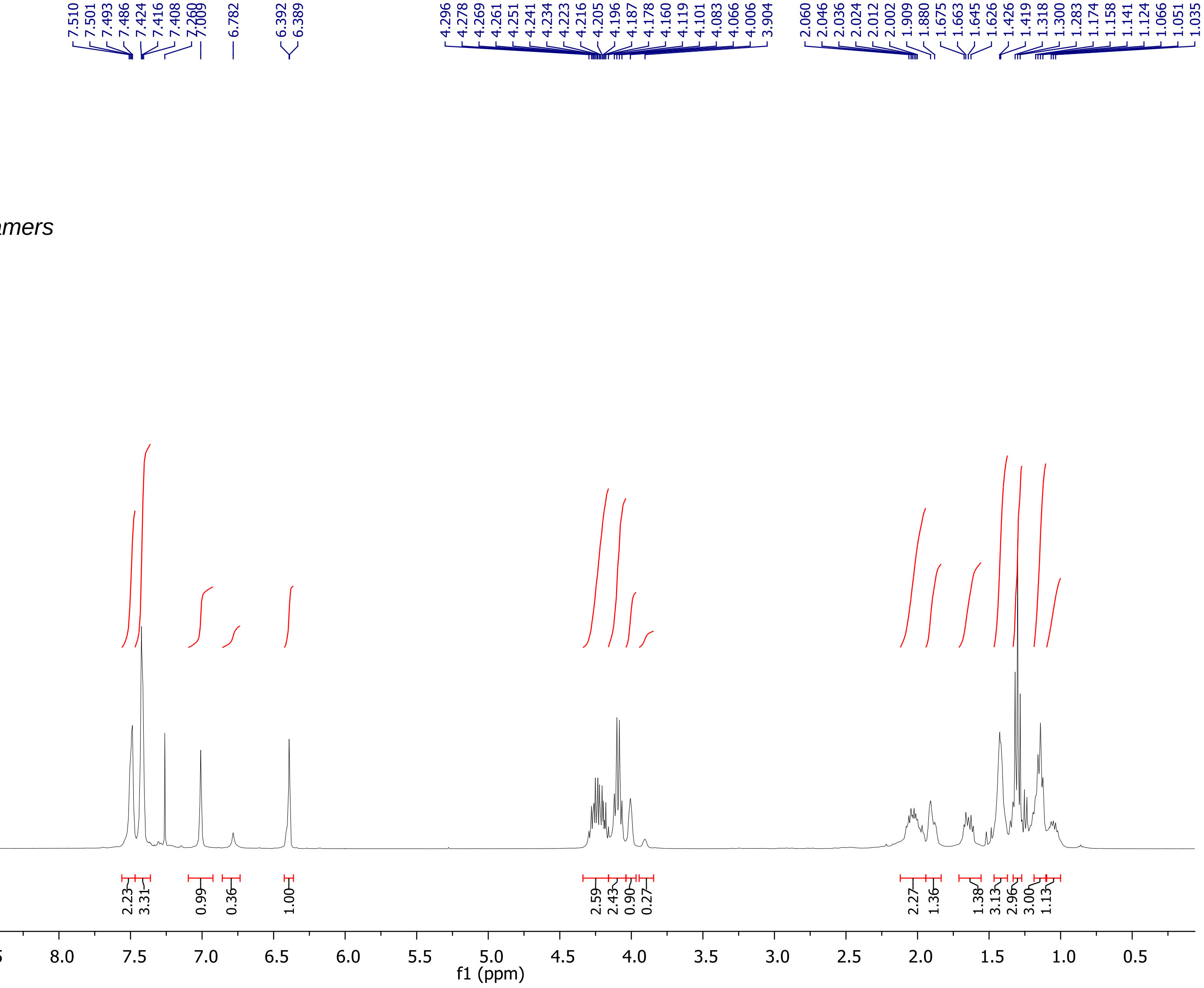

^{1}H NMR ($CDCl_3$, 400 MHz) of compound **34**

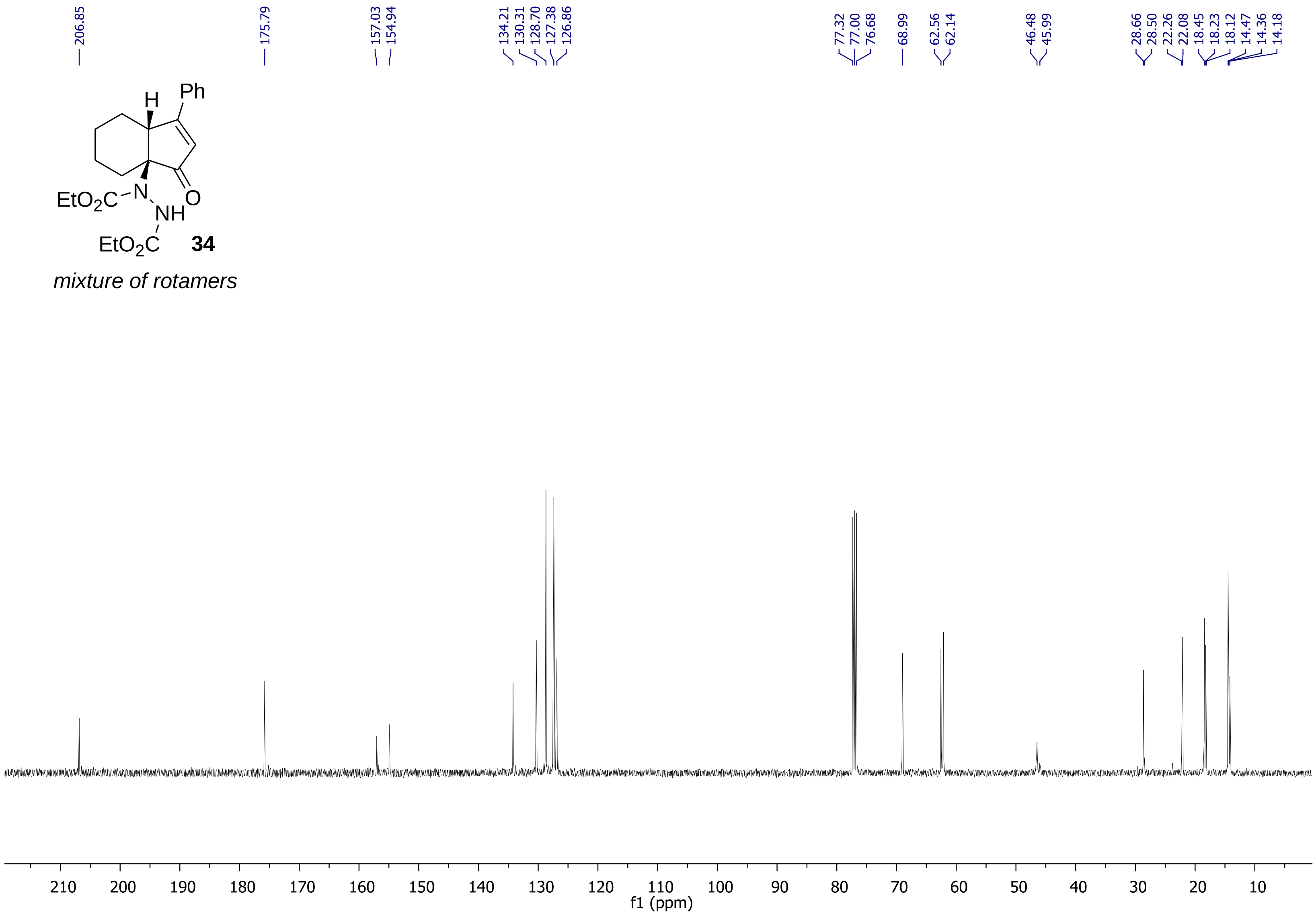

$^{13}C\{^{1}H\}$ NMR ($CDCl_3$, 100.4 MHz) of compound **34**

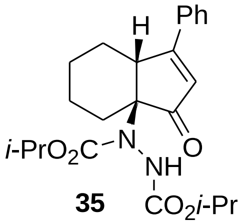

35

4.5 : 1 mixture of rotamers

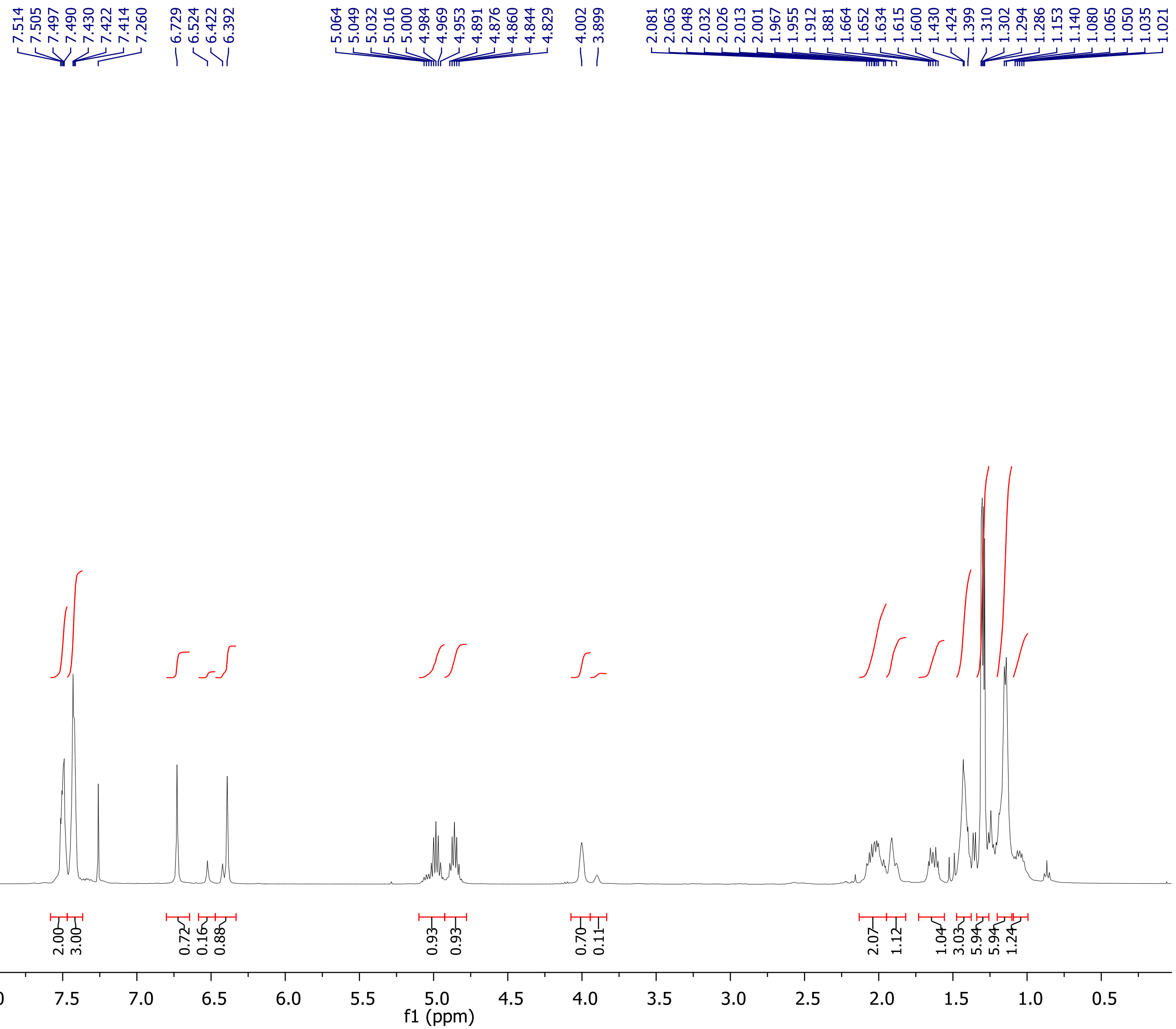

^{1}H NMR ($CDCl_3$, 400 MHz) of compound **35**

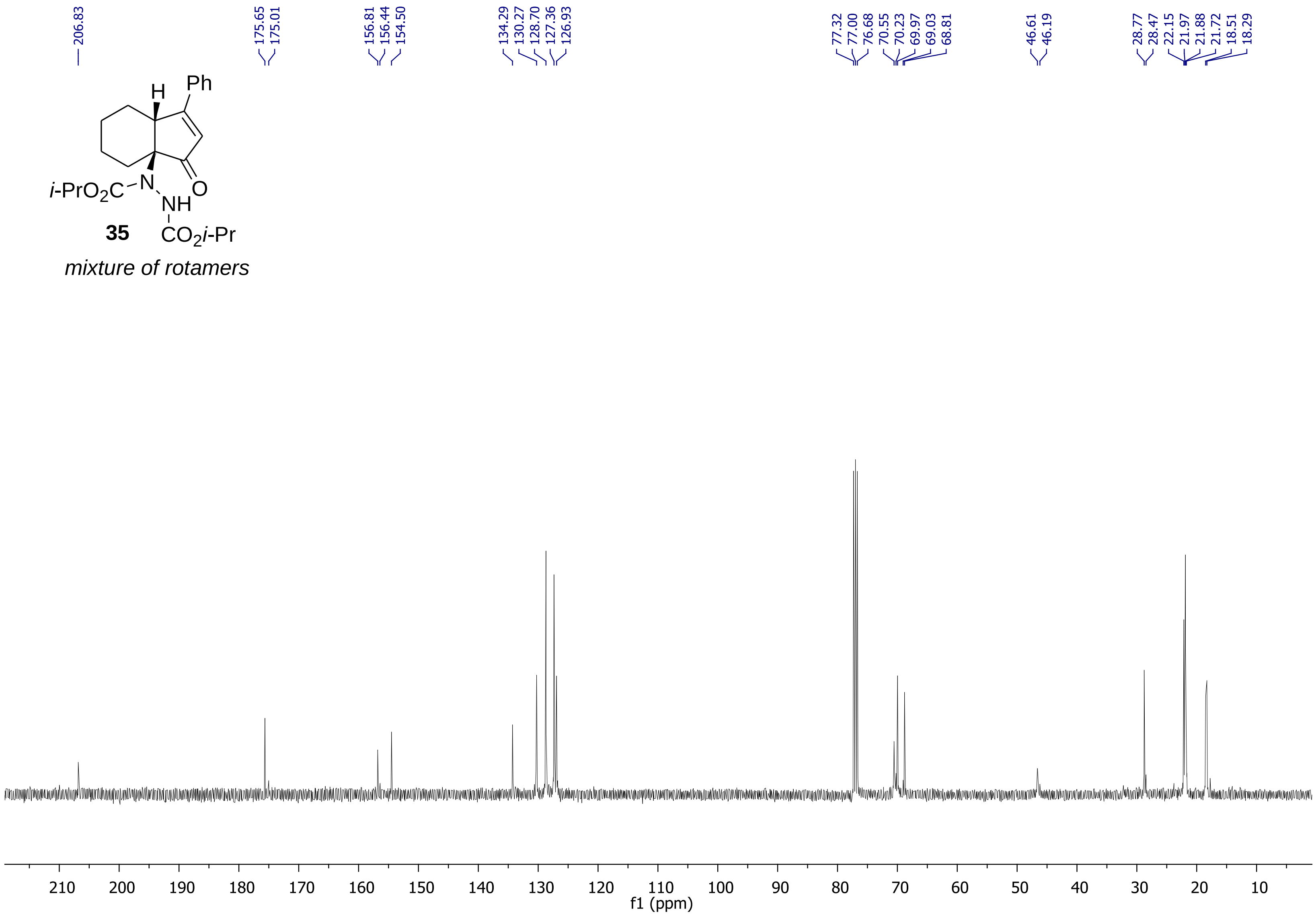

$^{13}C\{^{1}H\}$ NMR ($CDCl_3$, 100.4 MHz) of compound **35**

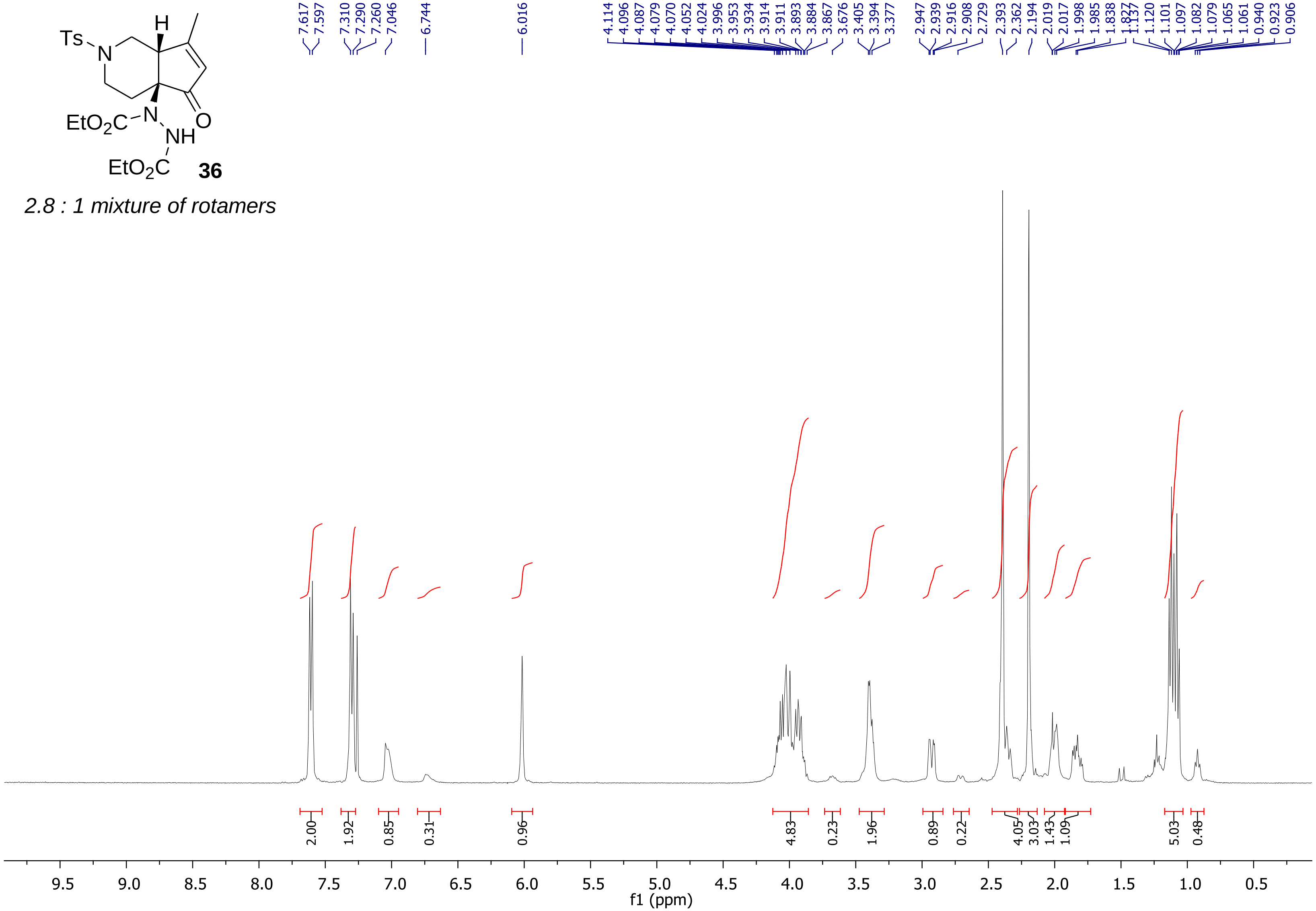

^{1}H NMR ($CDCl_3$, 400 MHz) of compound **36**

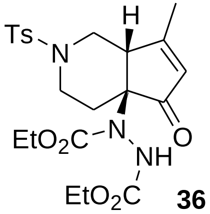

36

mixture of rotamers

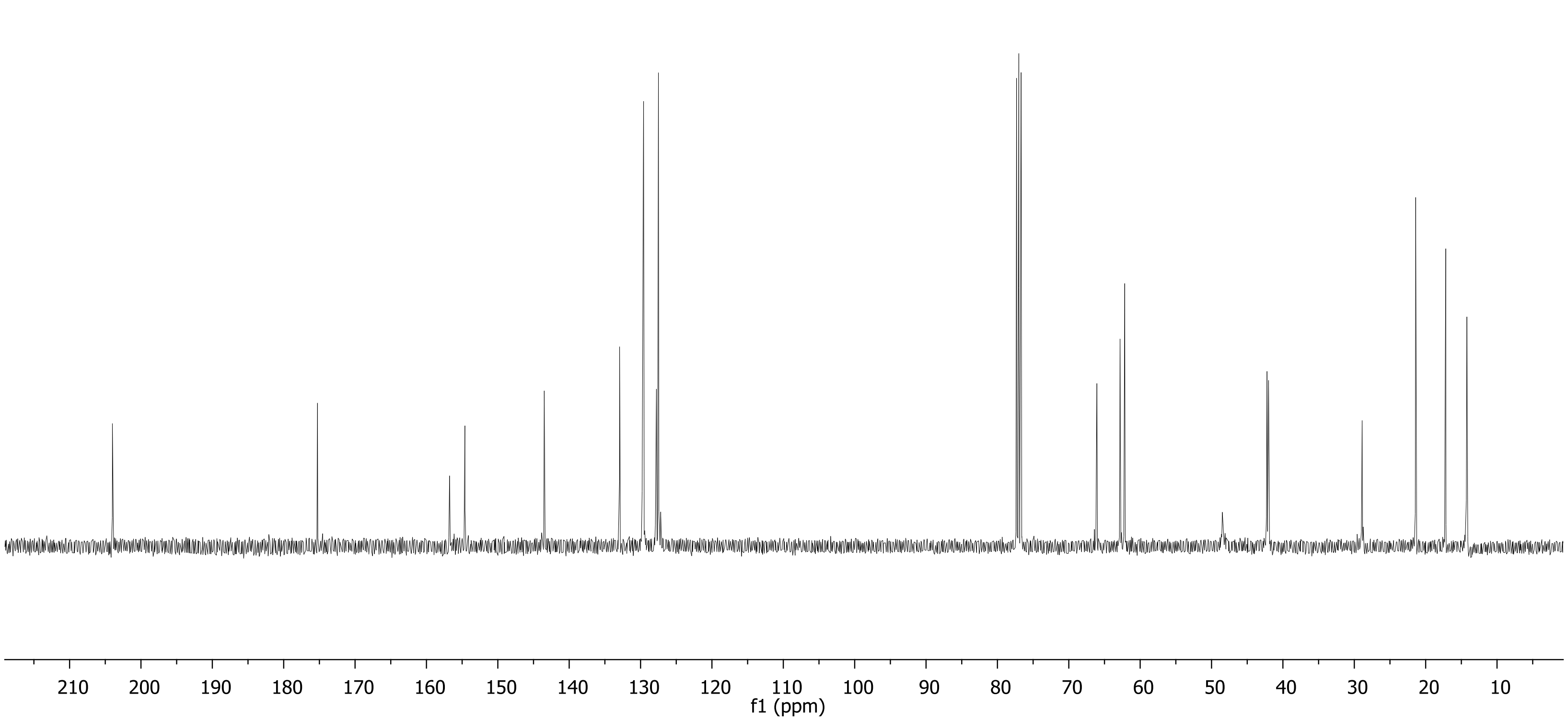

$^{13}C\{^{1}H\}$ NMR ($CDCl_3$, 100.4 MHz) of compound **36**

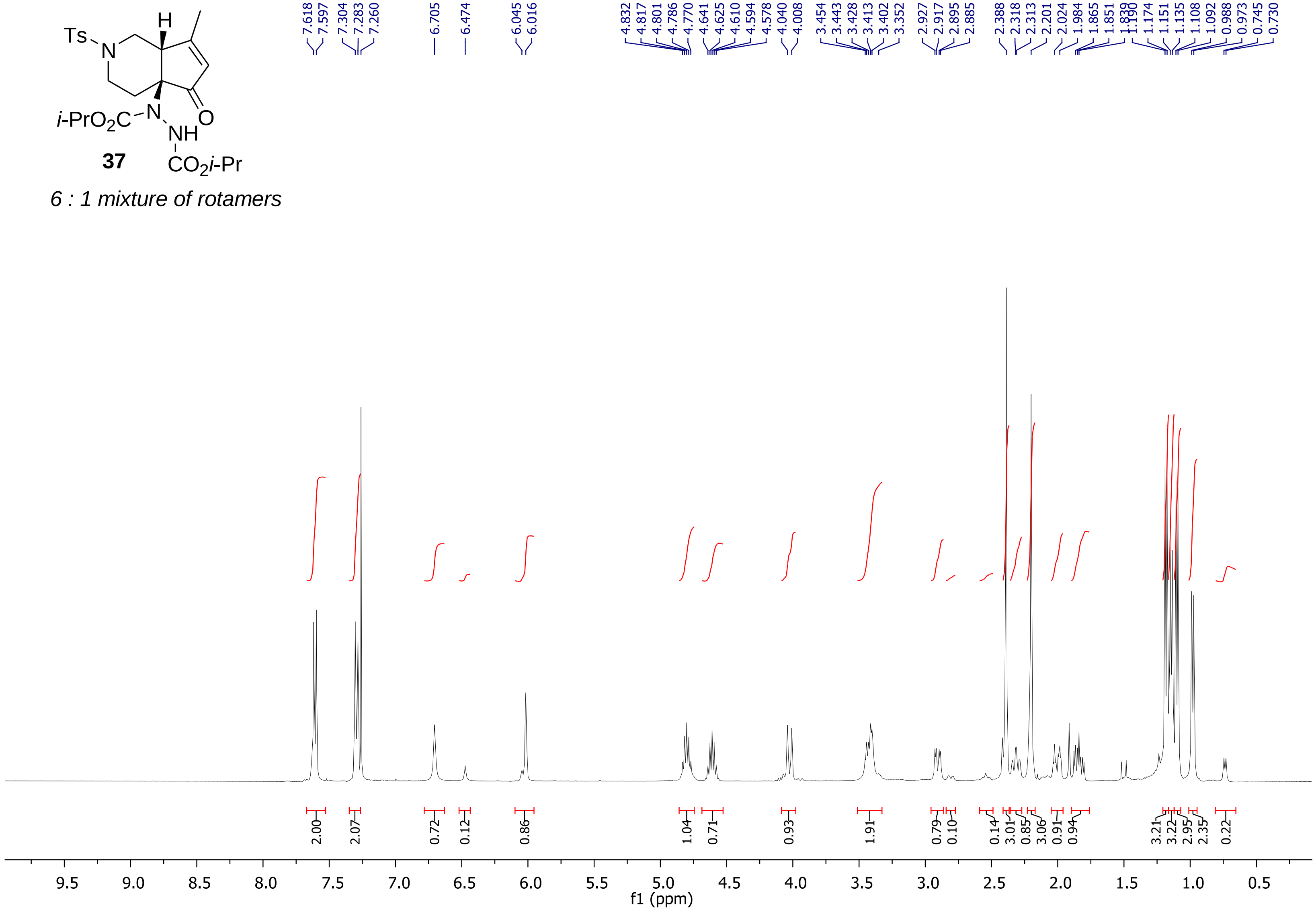

^{1}H NMR ($CDCl_3$, 400 MHz) of compound **37**

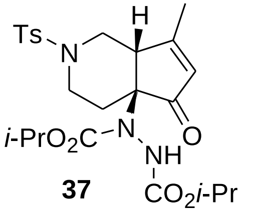

37

mixture of rotamers

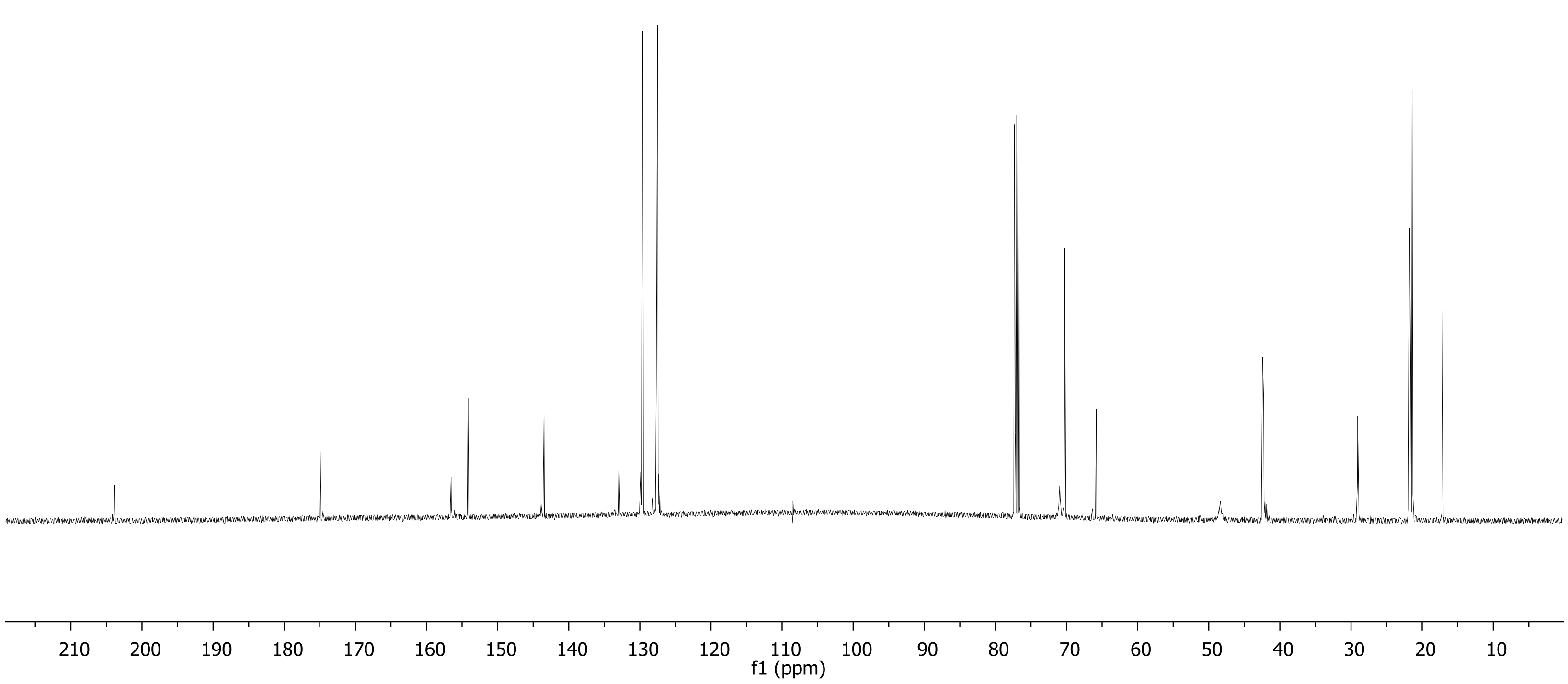

$^{13}C\{^1H\}$ NMR ($CDCl_3$, 100.4 MHz) of compound **37**

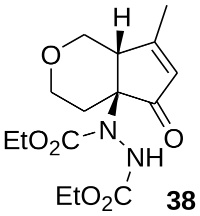

38

5.8 : 1 mixture of rotamers

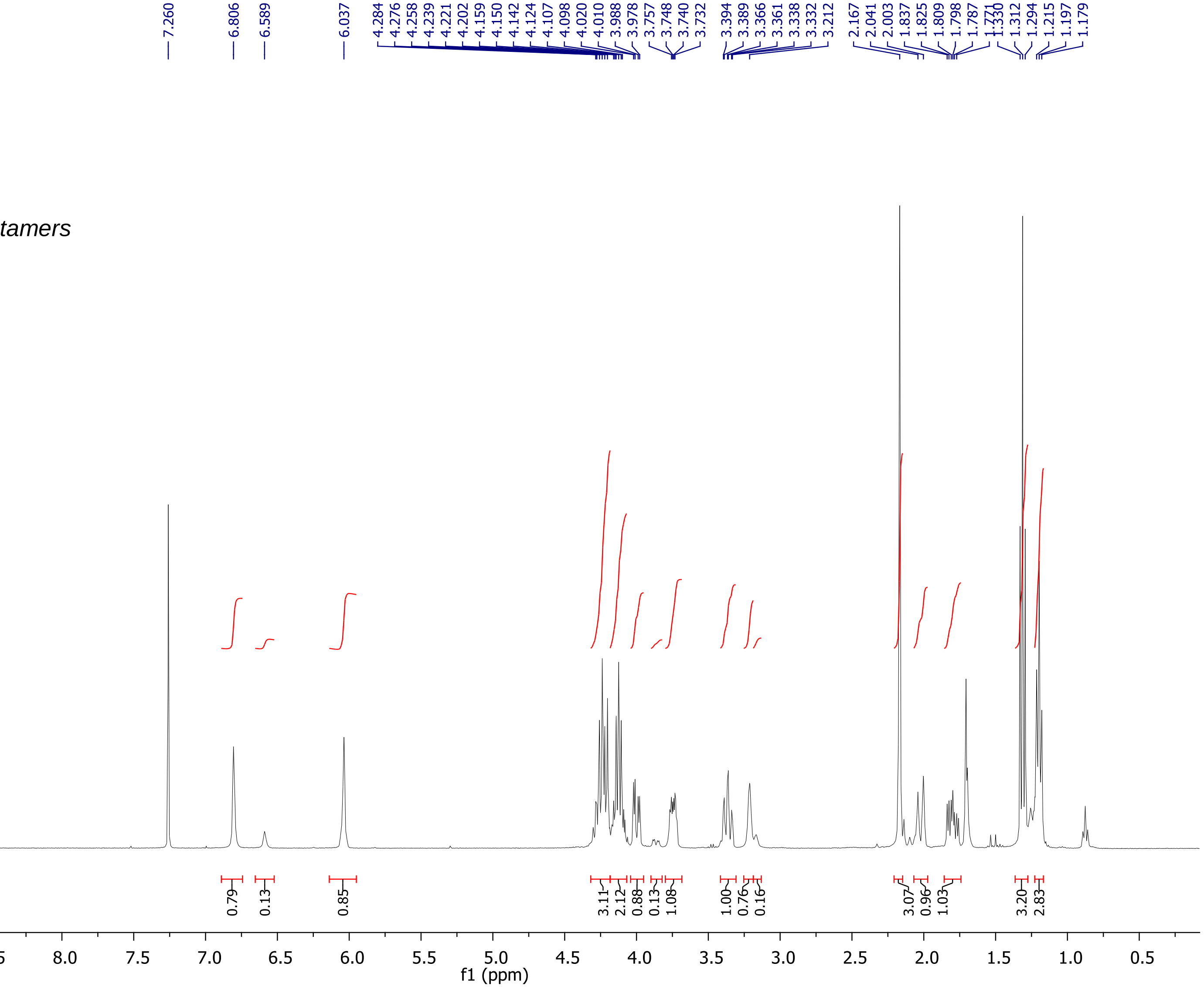

^{1}H NMR ($CDCl_3$, 400 MHz) of compound **38**

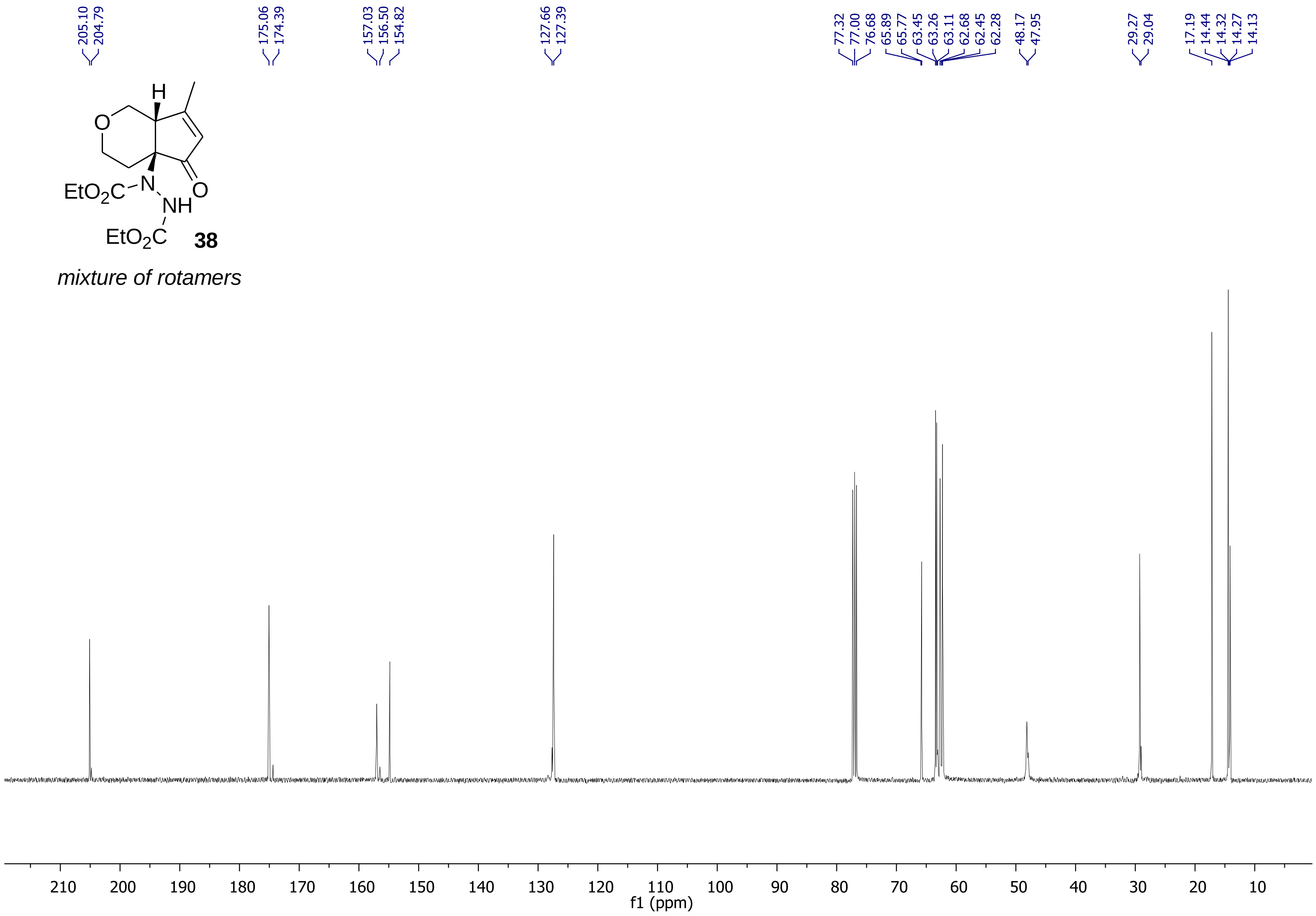

$^{13}C\{^{1}H\}$ NMR ($CDCl_3$, 100.4 MHz) of compound **38**

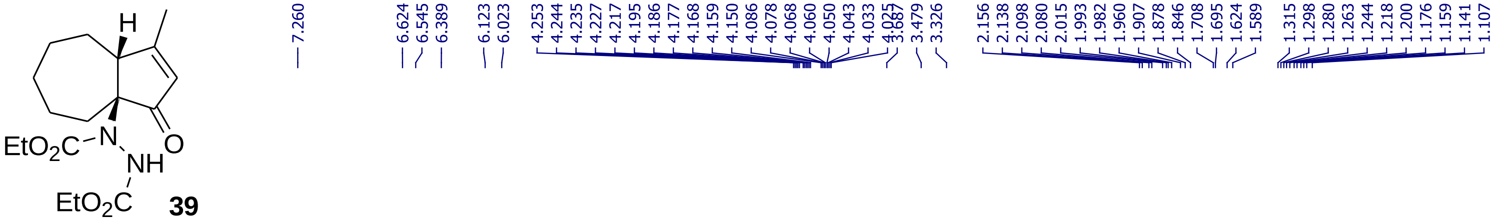

39

9 : 1 mixture of diastereoisomers
major diastereoisomer as 4.3 : 1 mixture of rotamers

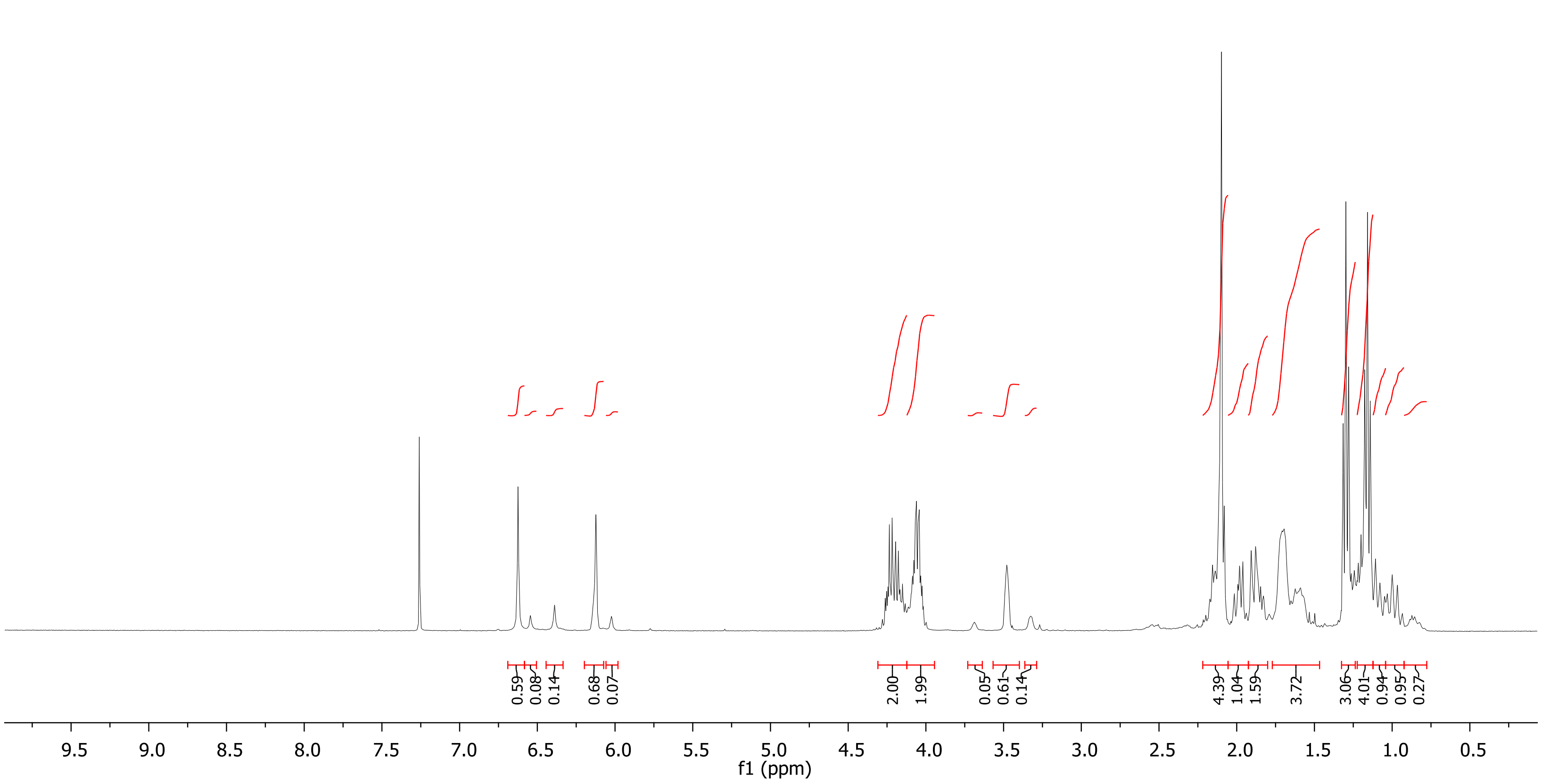

^{1}H NMR ($CDCl_3$, 400 MHz) of compound **39**

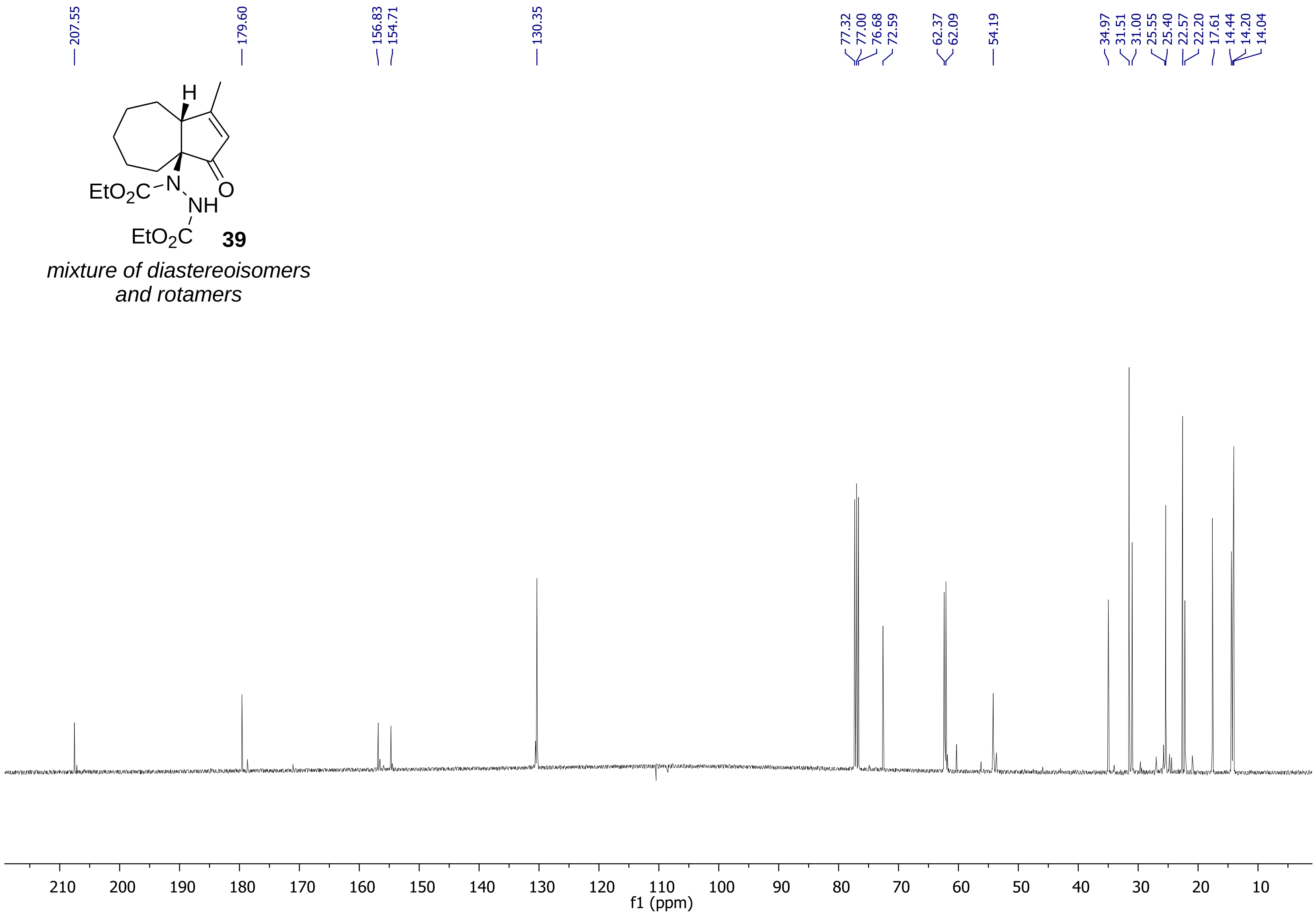

$^{13}C\{^{1}H\}$ NMR ($CDCl_3$, 100.4 MHz) of compound **39**

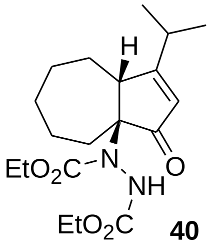

4.5 : 1 mixture of diastereoisomers
major diastereoisomer as 3 : 1 mixture of rotamers

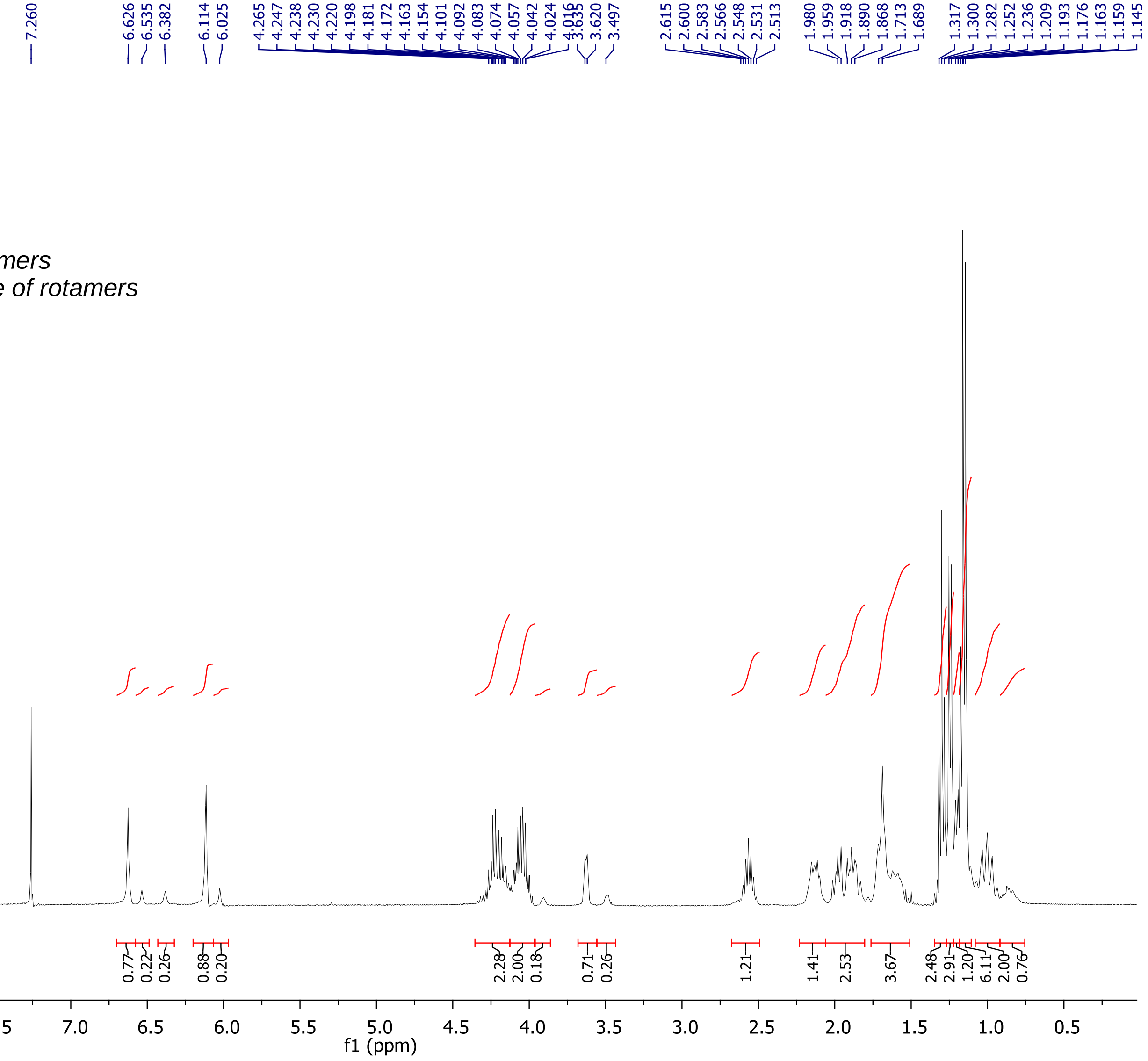

^{1}H NMR ($CDCl_3$, 400 MHz) of compound **40**

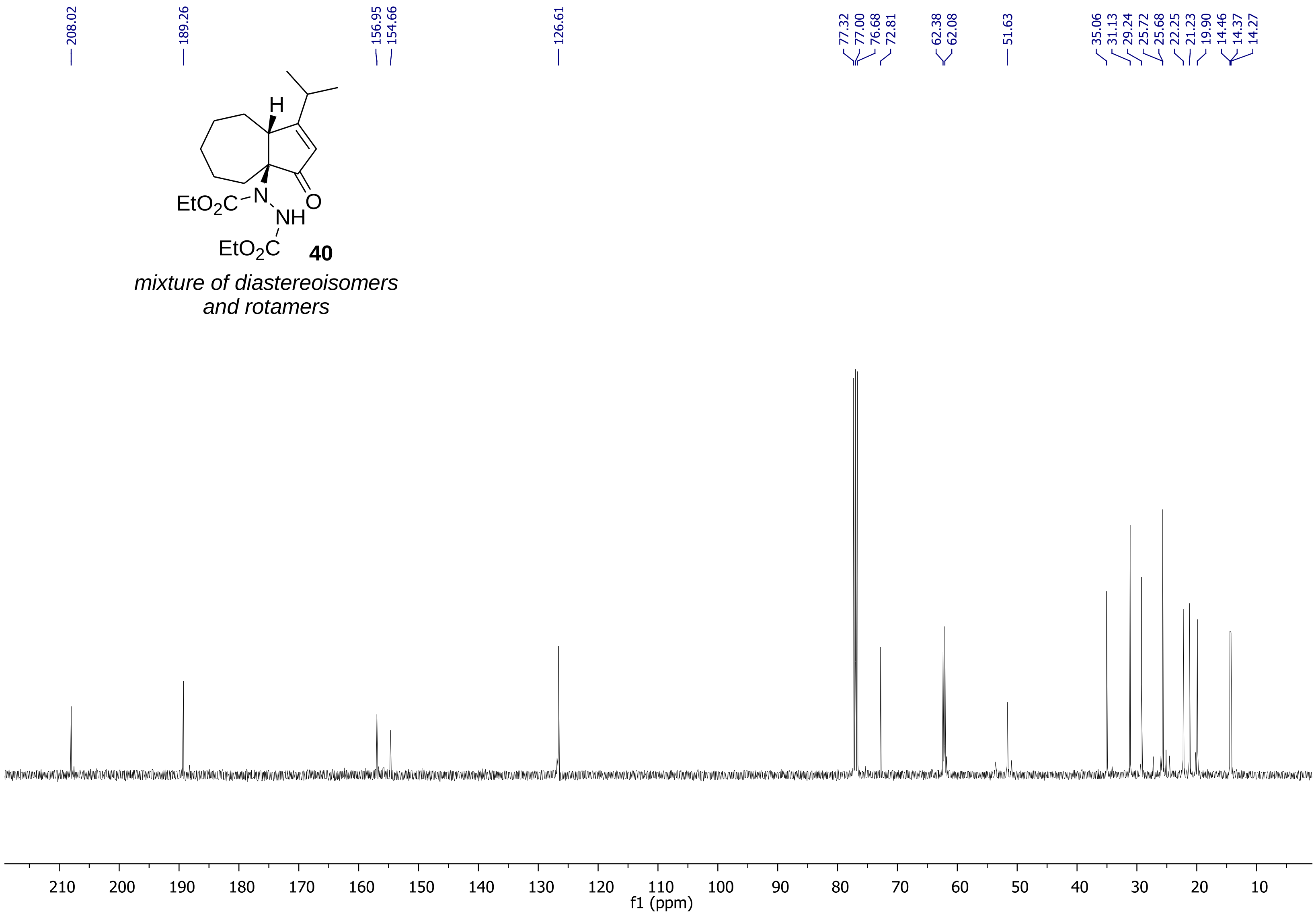

$^{13}C\{^{1}H\}$ NMR ($CDCl_3$, 100.4 MHz) of compound **40**

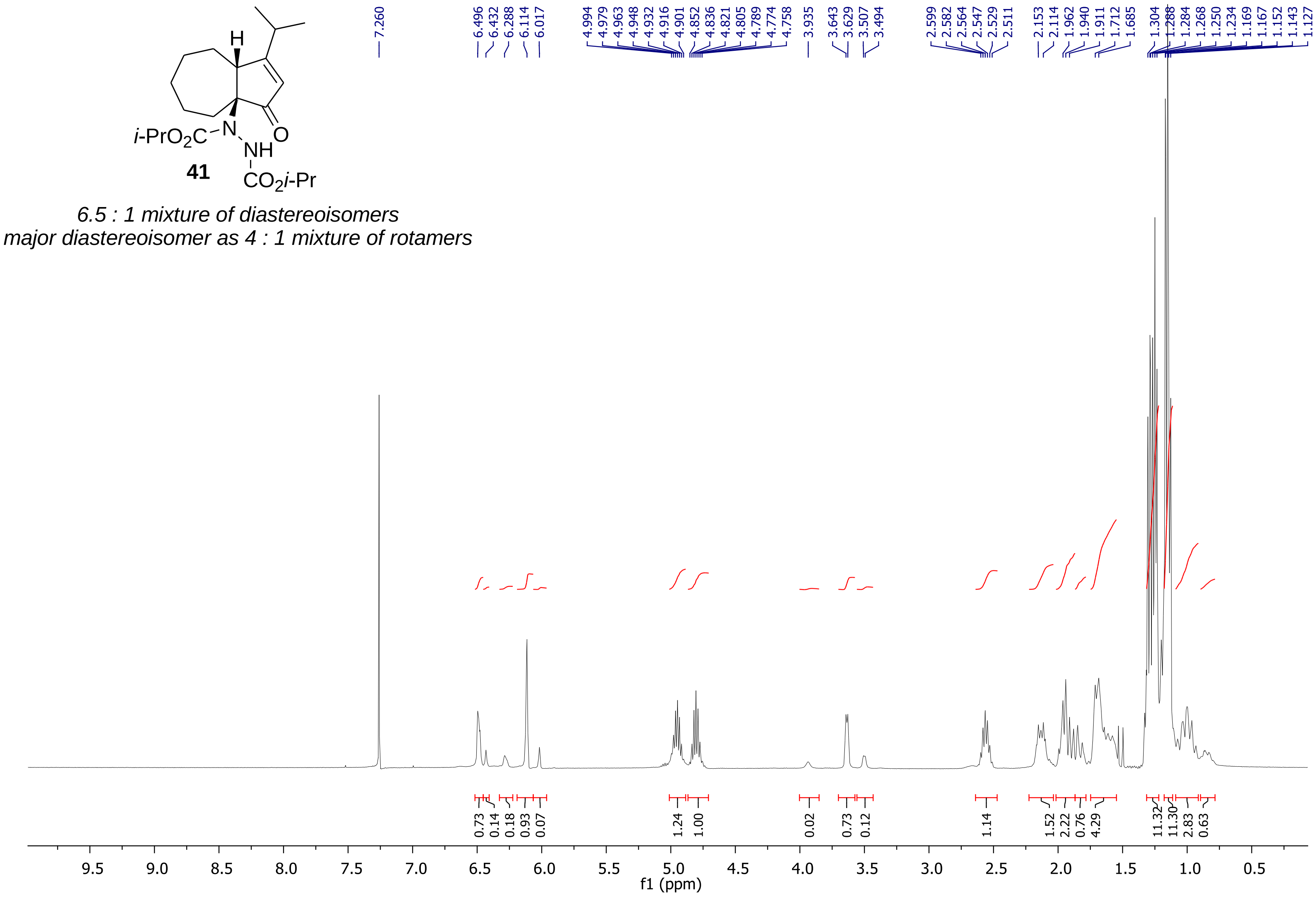

^{1}H NMR ($CDCl_3$, 400 MHz) of compound **41**

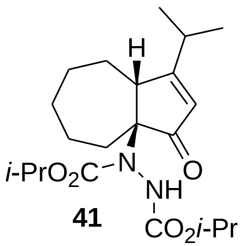

41

mixture of diastereoisomers and rotamers

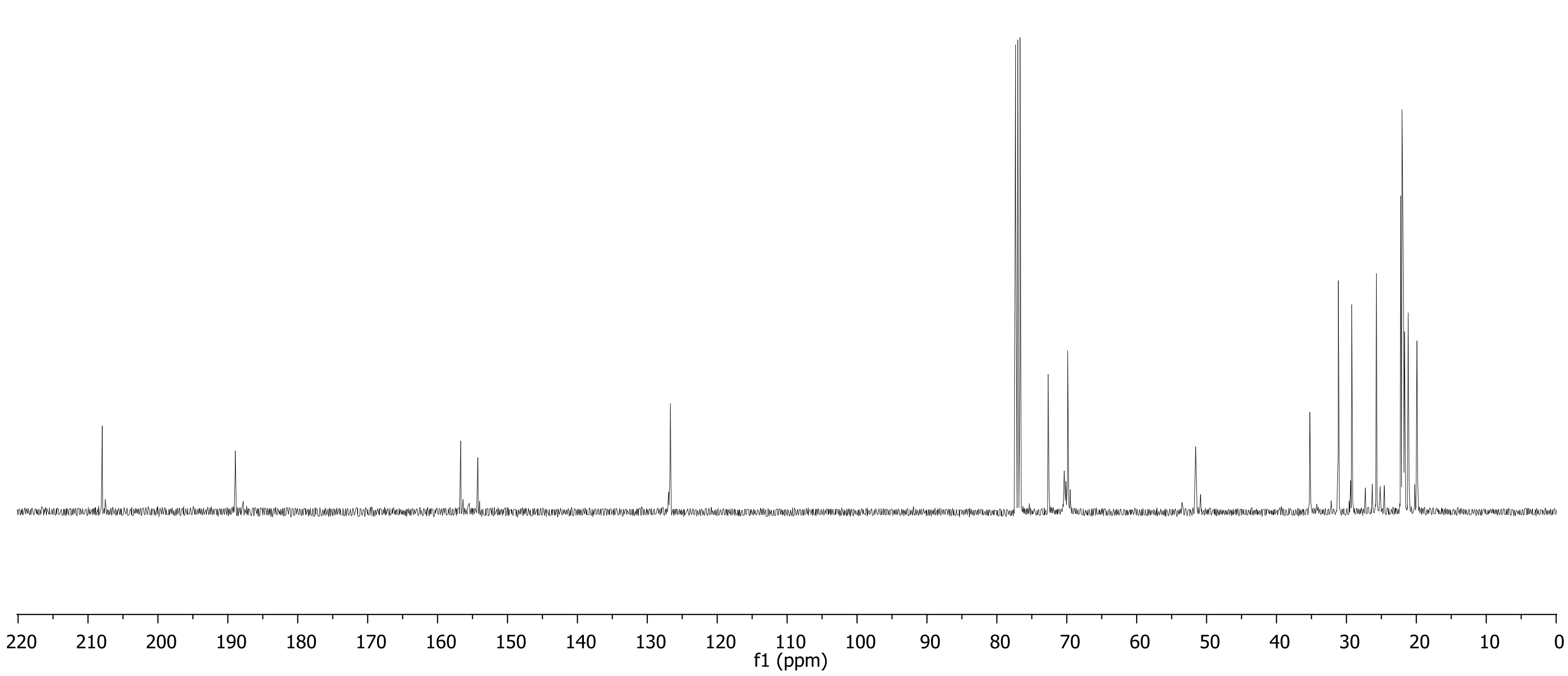

$^{13}C\{^1H\}$ NMR ($CDCl_3$, 100.4 MHz) of compound **41**

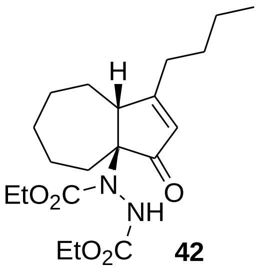

42

4.7 : 1 mixture of diastereoisomers
major diastereoisomer as 3.2 : 1 mixture of rotamers

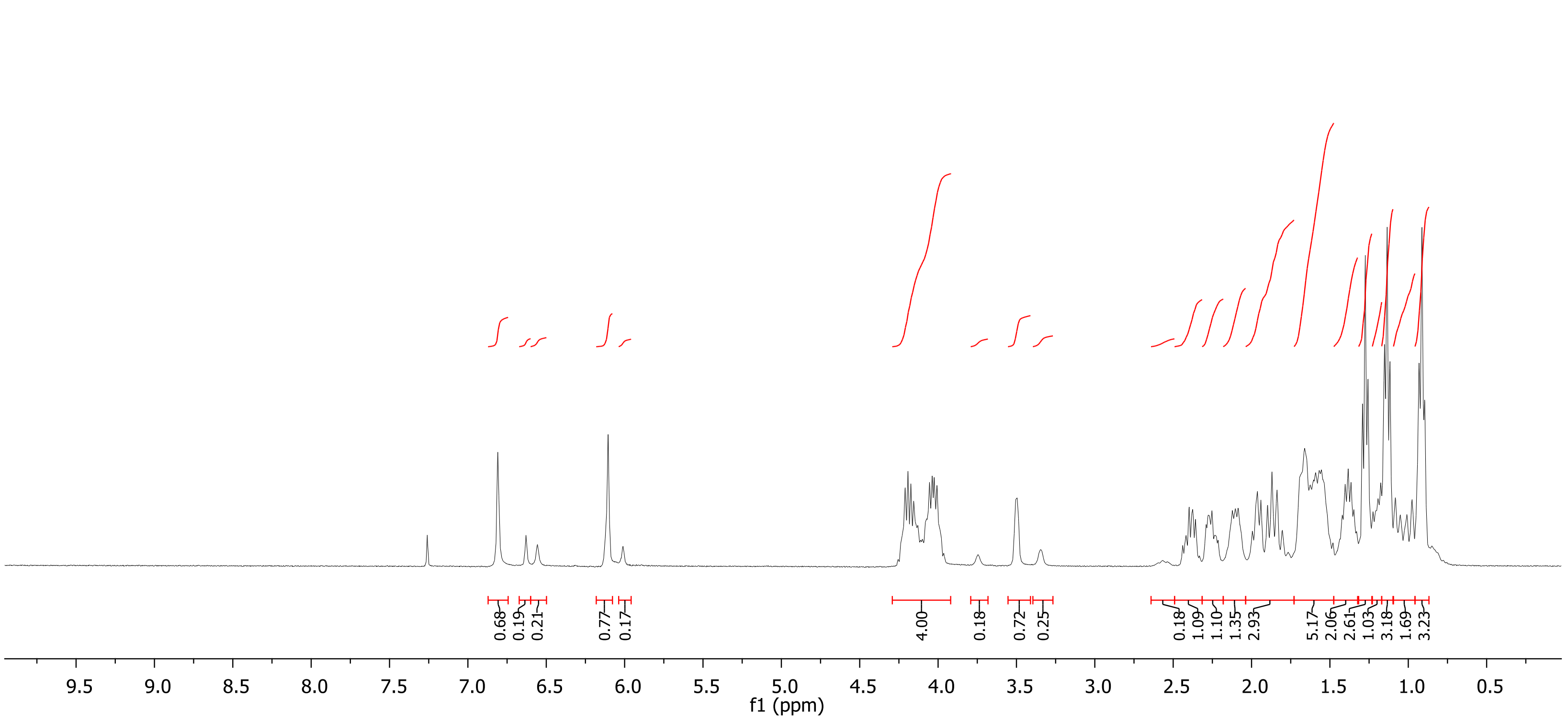

^{1}H NMR ($CDCl_3$, 400 MHz) of compound **42**

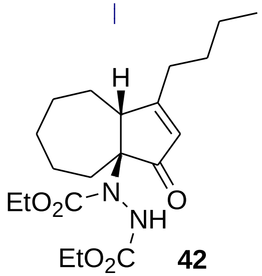

42

mixture of diastereoisomers and rotamers

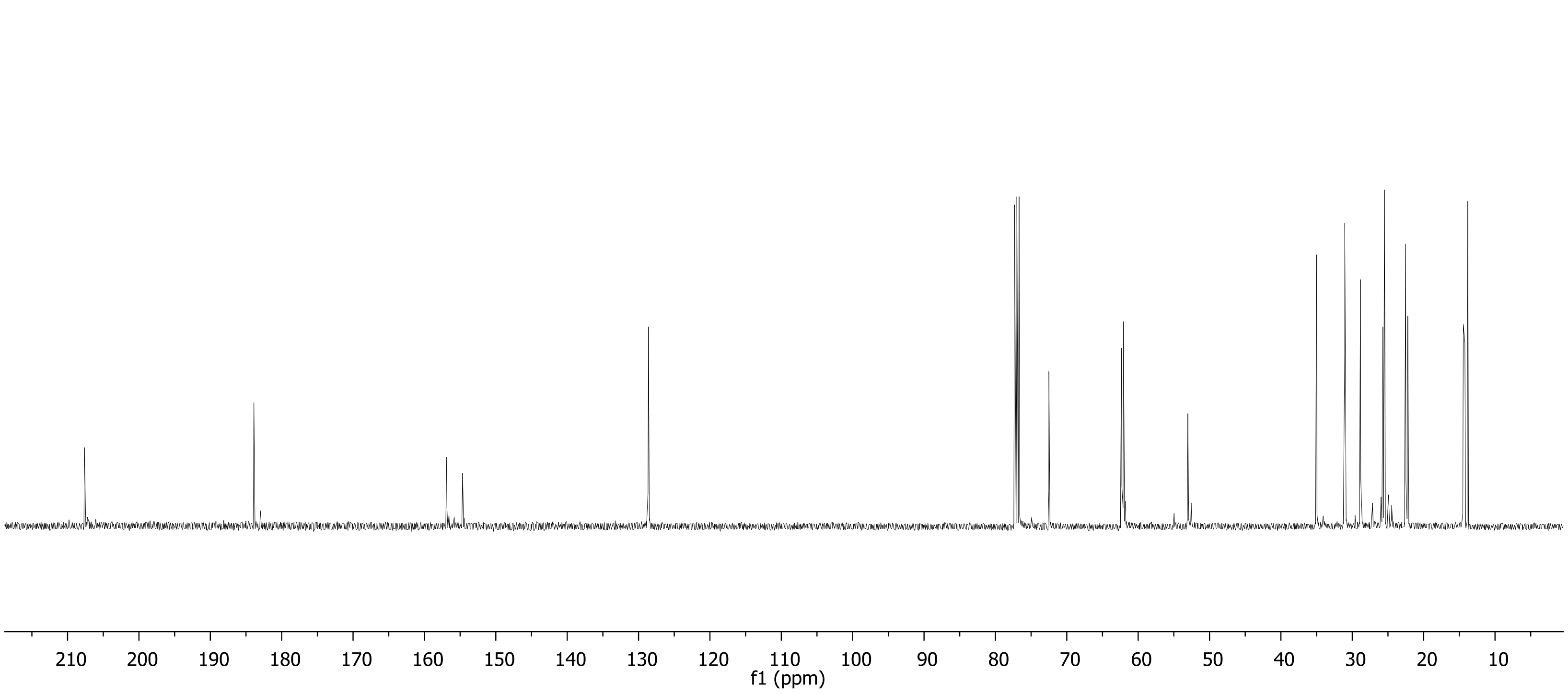

$^{13}C\{^{1}H\}$ NMR ($CDCl_3$, 100.4 MHz) of compound **42**

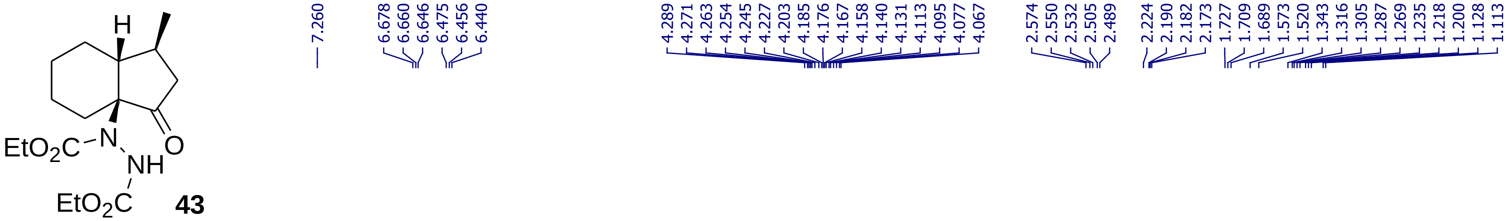

43

4.1 : 1 mixture of rotamers

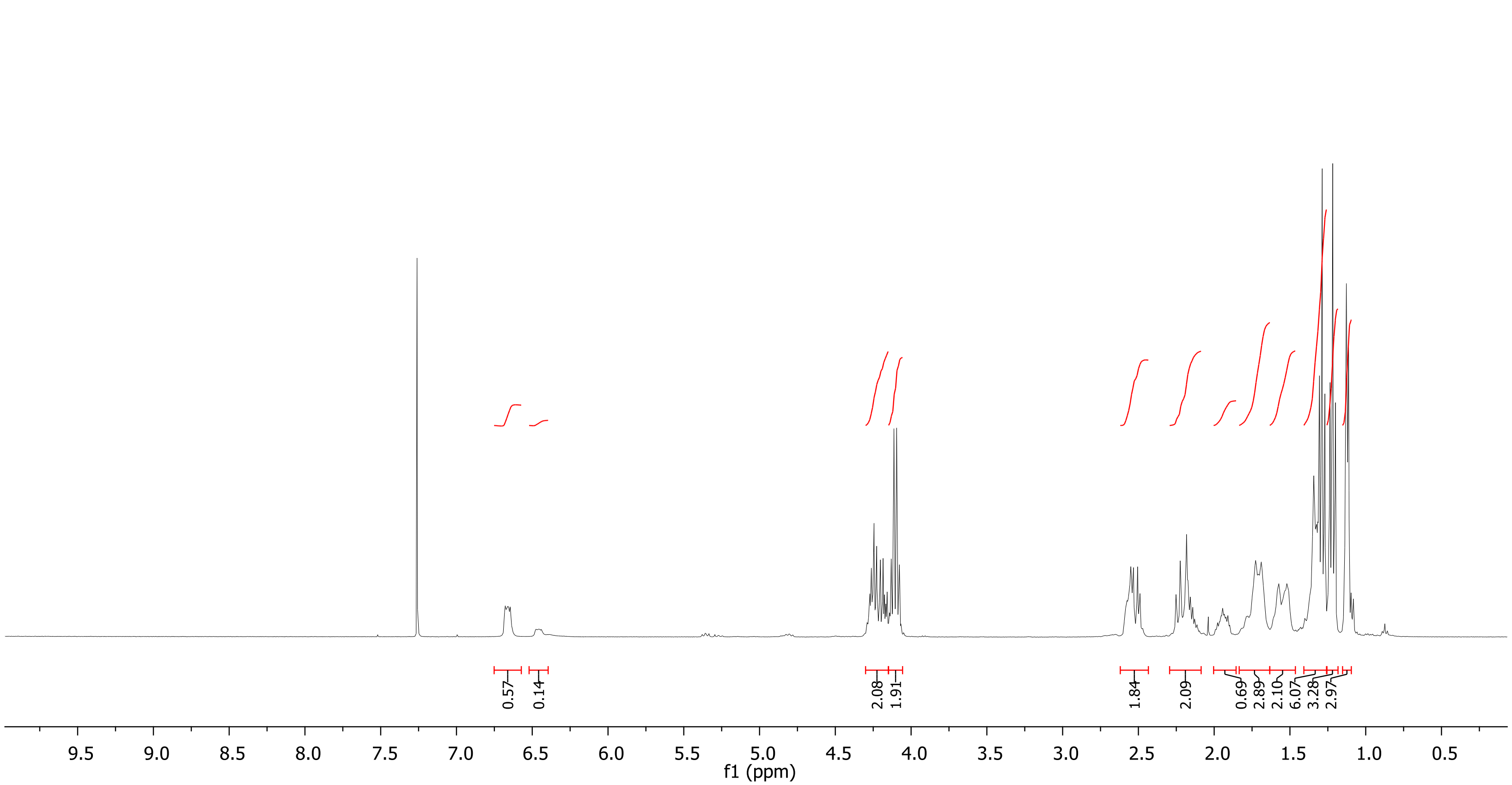

^{1}H NMR ($CDCl_3$, 400 MHz) of compound **43**

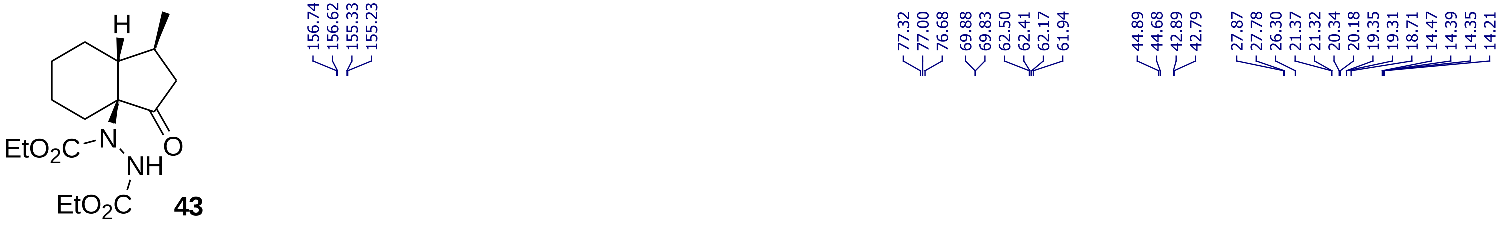

43

mixture of rotamers

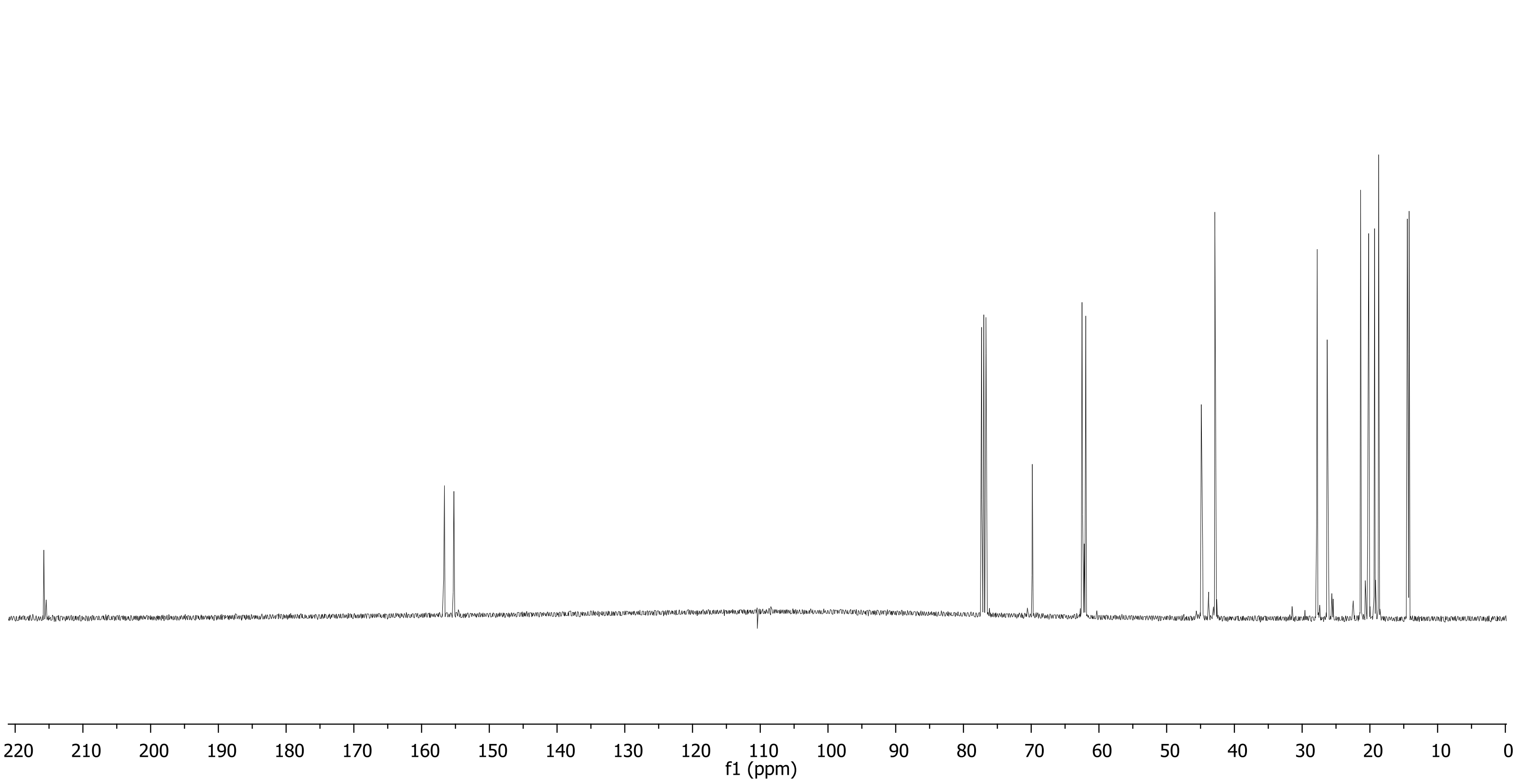

$^{13}C\{^{1}H\}$ NMR ($CDCl_3$, 100.4 MHz) of compound **43**

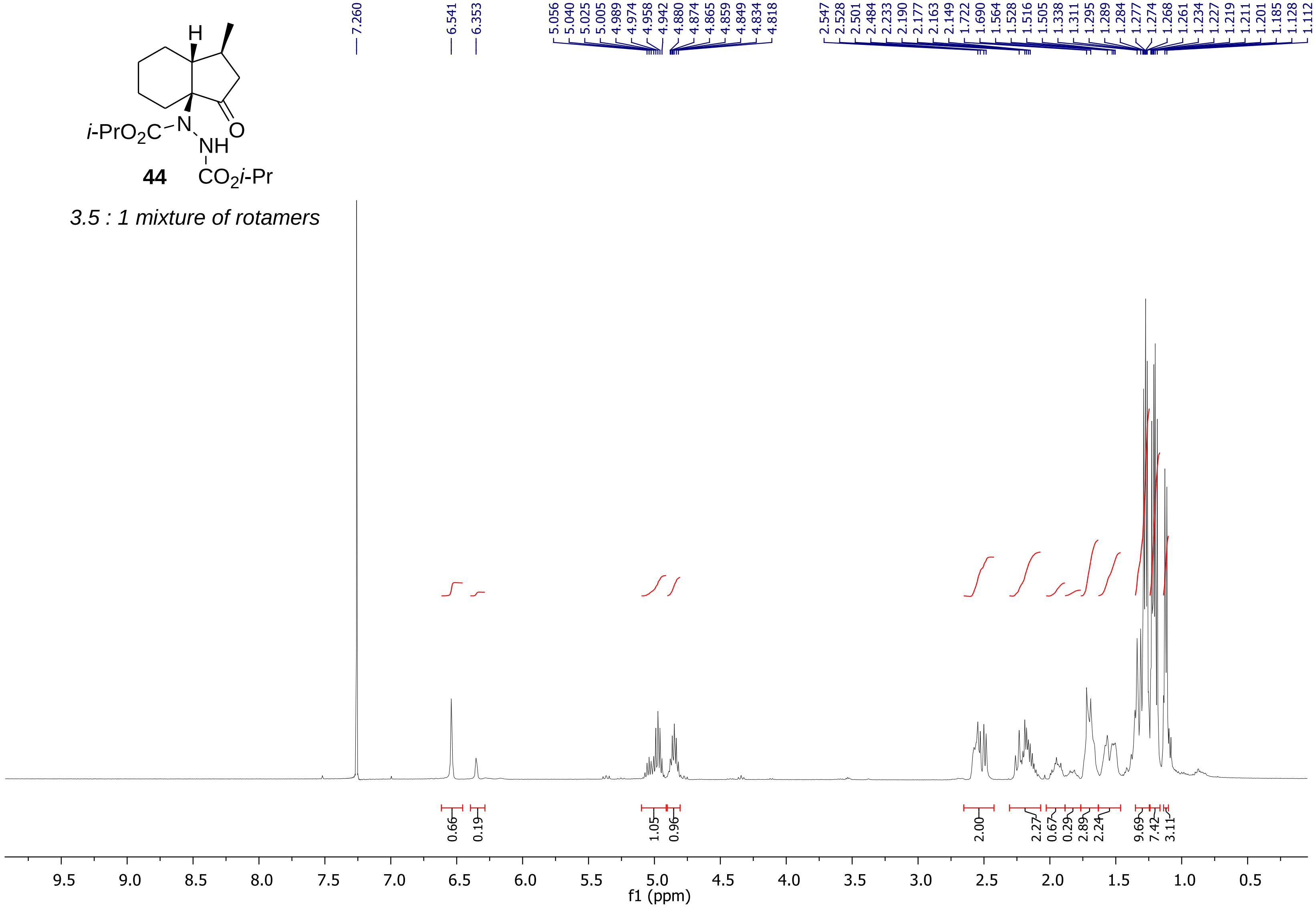

^{1}H NMR ($CDCl_3$, 400 MHz) of compound **44**

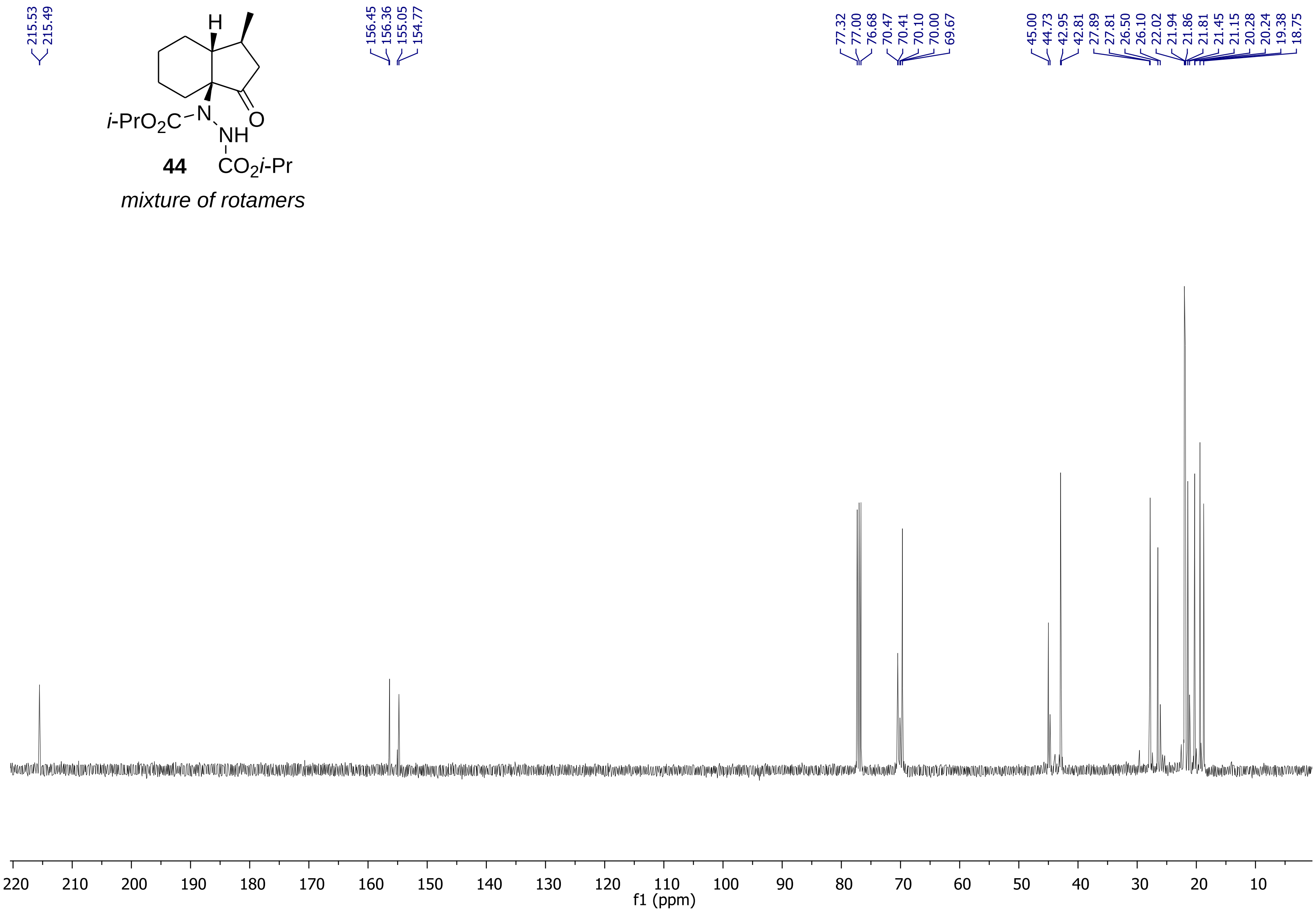

$^{13}C\{^1H\}$ NMR ($CDCl_3$, 100.4 MHz) of compound **44**

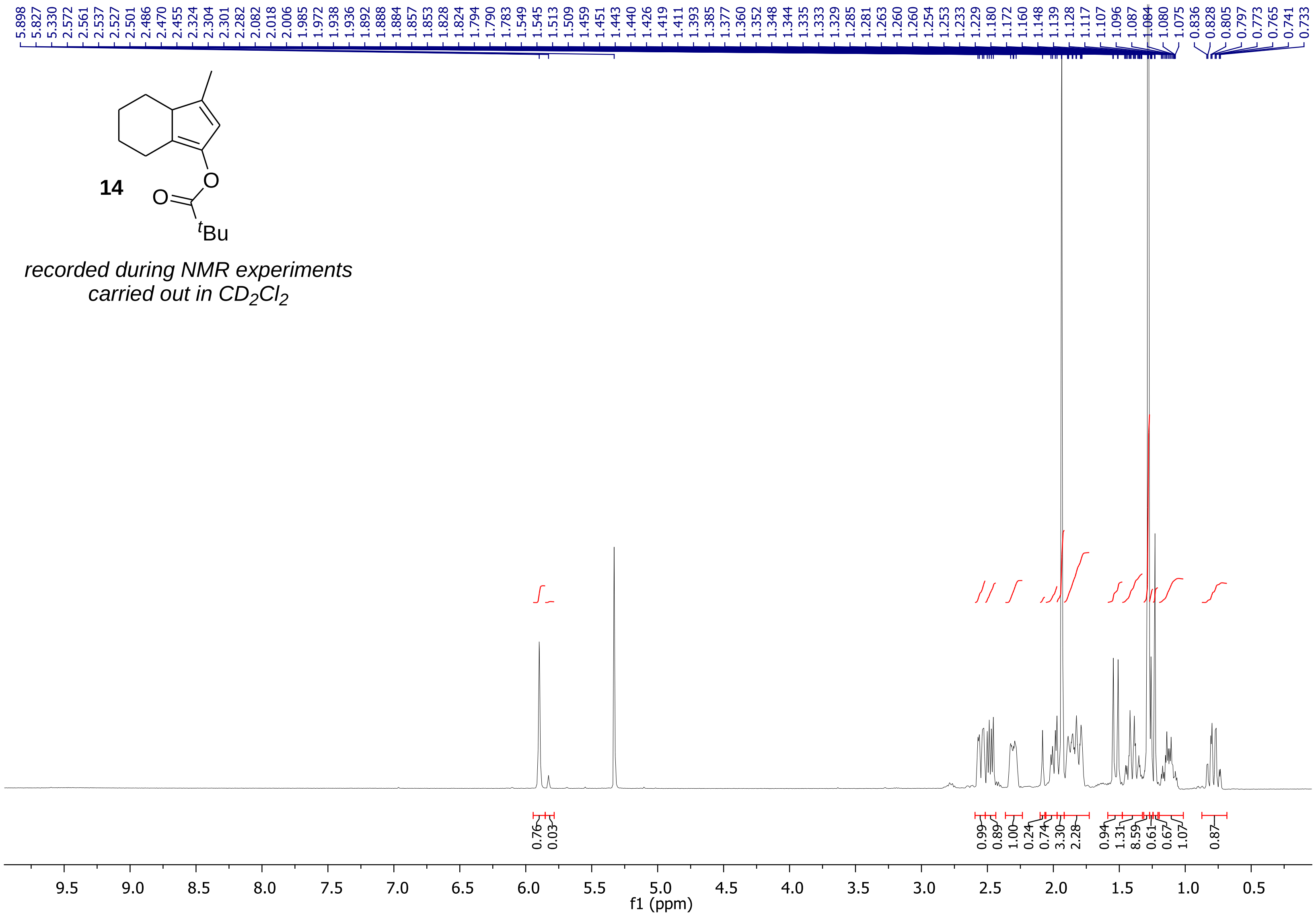

^{1}H NMR (CD_2Cl_2, 400 MHz) of compound **14** (recorded during NMR experiments)

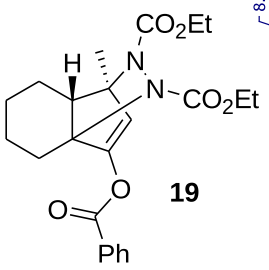

19

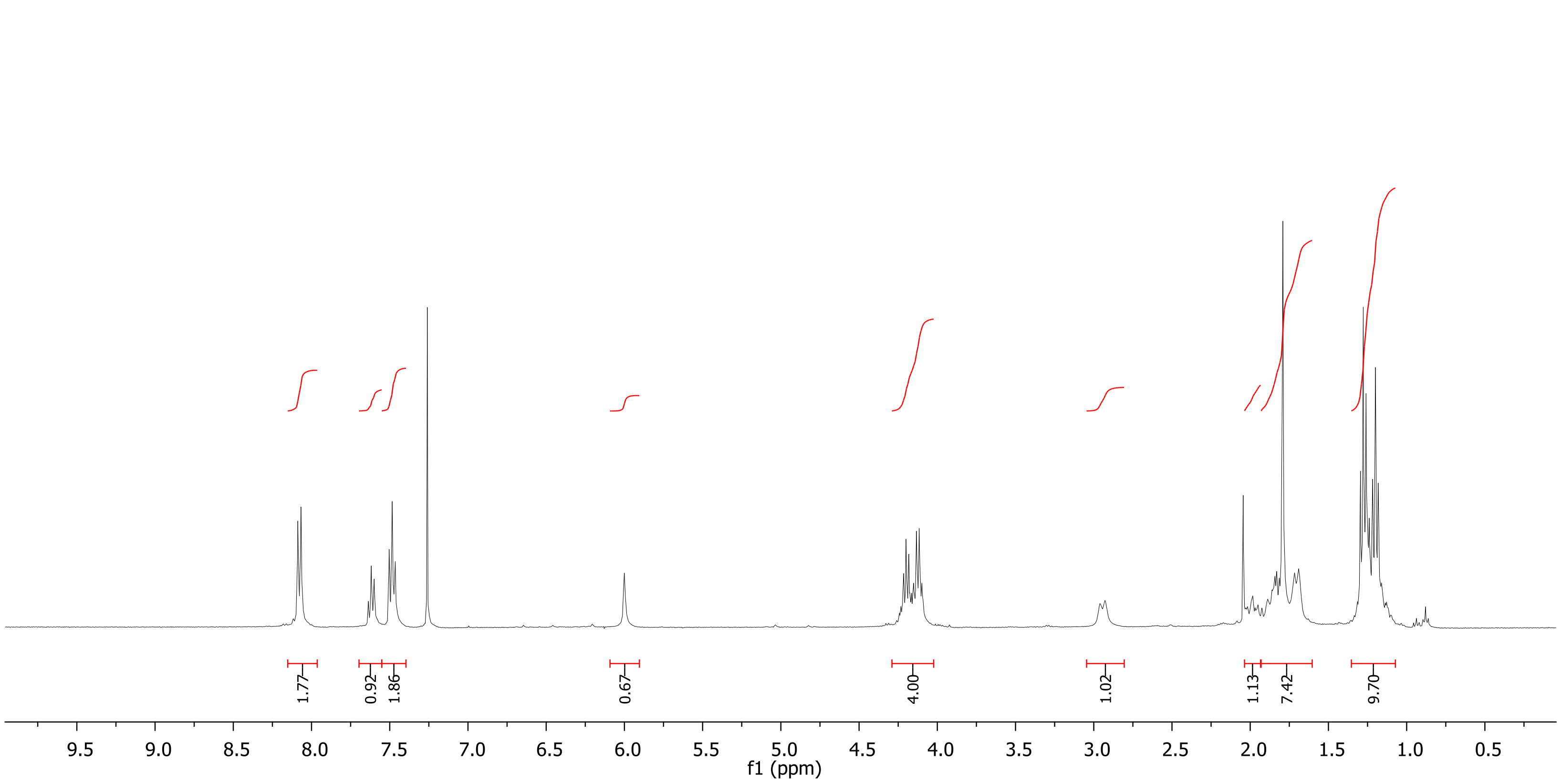

^{1}H NMR ($CDCl_3$, 400 MHz) of compound **19**

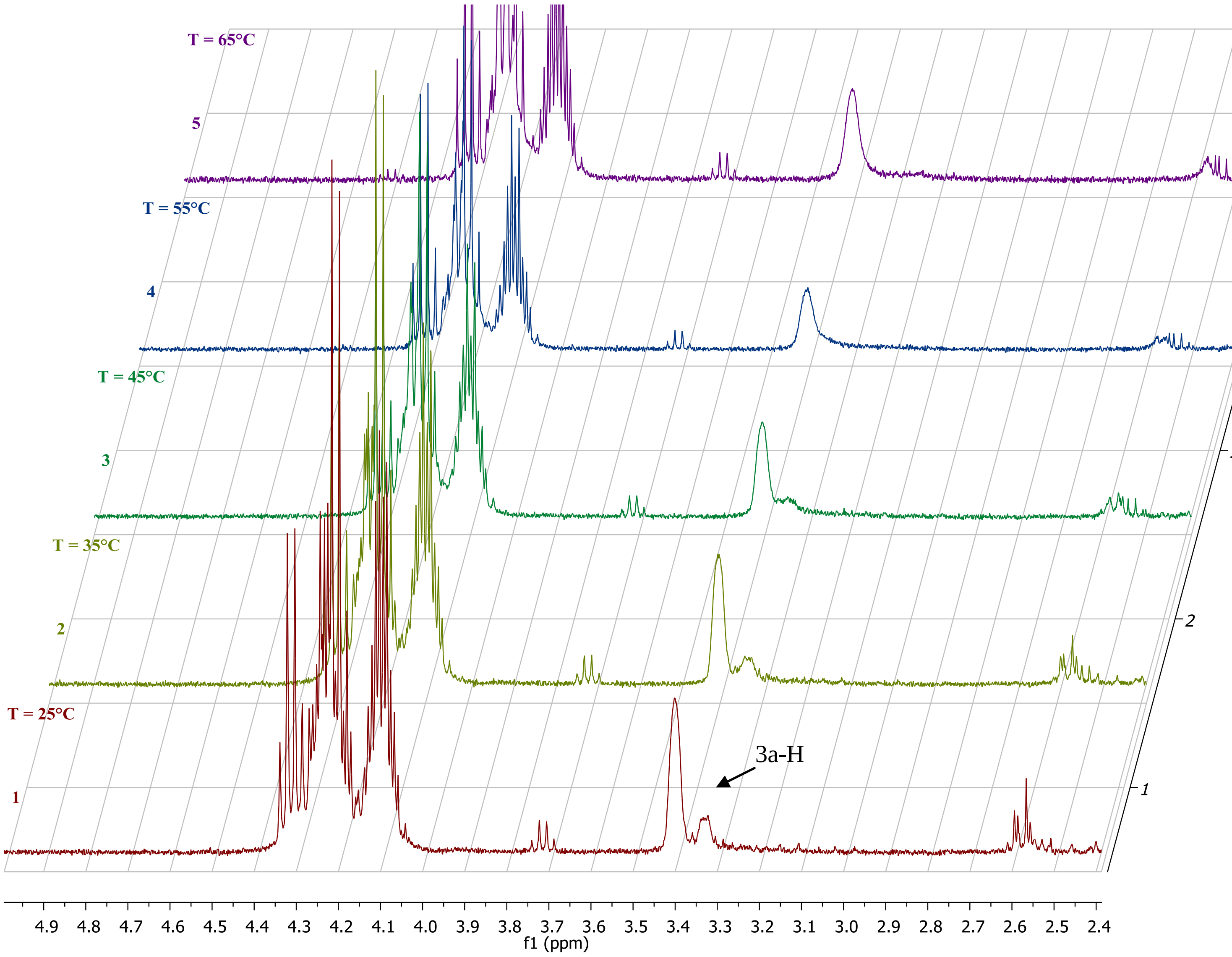

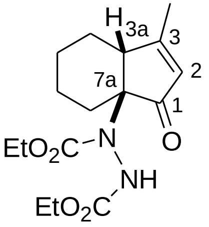

12

Enlarged view of ^{1}H NMR ($CDCl_3$, 400 MHz) of compound **12** recorded at variable T, ranging from 25 to 65 °C (10°C increments)
